# Heavy Metals Environmental Fate in Metallurgical Solid Wastes: Occurrence, Leaching, and Ecological Risk Assessment

**DOI:** 10.3390/jox15060211

**Published:** 2025-12-15

**Authors:** Shuqin Li, Guohua Ni

**Affiliations:** 1 Science Island Branch, Graduate School of USTC, Hefei 230026, China; ghni@ipp.ac.cn; 2 Hefei Institutes of Physical Science, Chinese Academy of Sciences, Hefei 230031, China; 3 Sinosteel Maanshan General Institute of Mining Research Co., Ltd., Maanshan 243071, China

**Keywords:** metallurgical solid waste, heavy metals, occurrence, leaching characteristics, environmental risk assessment

## Abstract

The metallurgical industry generates substantial amounts of heavy metal-containing solid waste, posing significant environmental and health risks. This study systematically evaluates the environmental behavior and ecological risks of heavy metals in four typical metallurgical wastes: jarosite slag (SW1), electric arc furnace ash (SW2), chromium-containing sludge (SW3), and acid-base sludge (SW4). We demonstrate that particle size fundamentally governs heavy metal mobility, with fine-structured SW1 and SW2 (D_50_ = 4.76 µm and 1.34 µm) exhibiting enhanced metal mobility and bioavailability. In contrast, coarser SW3 and SW4 particles (D_50_ = 268.83 µm and 133.94 µm) retain heavy metals in more stable forms. Among all metals analyzed, cadmium (Cd) presents the most severe ecological threat, with acid-extractable fractions reaching 52% in SW2 and 45% in SW3—indicating high release potential under changing pH conditions. Risk assessment confirms high to very high ecological risks for Cd in both SW2 and SW3. Moreover, under acidic leaching conditions, SW1 and SW2 show significantly higher cumulative toxicity than SW3 and SW4. These findings highlight the critical role of waste-specific properties in controlling heavy metal fate and provide a scientific basis for targeted risk management and sustainable remediation strategies.

## 1. Introduction

The metallurgy industry, a critical sector of the national economy, plays an indispensable role in driving both global economic growth and societal progress [1,2]. In 2020, China’s crude steel production reached 1.065 billion tons, accounting for 56.7% of the global total [3]. Moreover, China holds a significant share in the global production of non-ferrous metal alloys, including iron, copper, and nickel, contributing approximately half of the global output [4,5]. However, this large-scale production generates a substantial amount of metallurgical solid waste, which is complex in composition and presents significant environmental and health risks [6,7]. In the absence of comprehensive pre-assessment and appropriate treatment, such waste not only results in resource loss but also poses serious threats to the ecosystem and public health [8].

Strengthening systematic research on metallurgical solid waste, including the identification of its composition, pollution characteristics, and potential hazards, has become essential for the scientific management and resource utilization of such waste [9,10]. This will enhance waste management practices while also providing a critical foundation for the green transformation and sustainable development of the metallurgy industry. Recent research has focused on the occurrence forms, leaching characteristics, and health risk assessments of heavy metals in metallurgical solid waste. For instance, Pang et al. [11]., employed both experimental and modeling approaches to analyze the distribution and environmental risks of heavy metals in electrolytic manganese residue (EMR), lead–zinc slag (LZS) and electric-furnace ferronickel slag (EFS). They found that manganese in EMR is readily leached under neutral conditions, whereas heavy metals in LZS and EFS are leached under acidic conditions, with leaching subsequently controlled by complexation and precipitation in neutral or alkaline environments. Anna and her team [12] observed that the leaching of heavy metals in metallurgical solid waste is significantly affected by pH, with most metals exhibiting lower concentrations at higher pH values. However, lead leaching increases under alkaline conditions, and copper leaching notably rises at a pH of 10.5. Li et al. [13]., emphasized that although certain metals in metallurgical solid waste may have high total concentrations, their leachability and environmental risks depend primarily on their speciation and mobility. Therefore, sequential extraction tests are necessary to assess the release potential and environmental impact of pollutants, ensuring safe reuse or proper disposal.

Jarosite slag, electric arc furnace ash, chromium-containing sludge, and acid-base sludge are typical solid wastes in the metallurgy industry [14]. These wastes attract significant attention due to their complex origins, diverse compositions, and significant environmental risks. Jarosite slag primarily result from the use of the iron salt process in hydrometallurgical extraction of zinc, copper, and nickel, with the highest production occurring during zinc smelting [15]. Current disposal methods for jarosite slag include landfilling, hazardous waste treatment, rotary kiln, and plasma furnace processes [16]. However, due to technological and economic constraints, large quantities of jarosite slag continue to be temporarily stored, posing considerable environmental pollution risks. Electric arc furnace ash, a byproduct of steelmaking in electric arc furnaces, contains up to 40–50% iron and includes harmful heavy metals such as lead, chromium, and arsenic, presenting significant environmental hazards [17]. Chromium-containing sludge, primarily produced during steel plate coating processes, contains chromium concentrations ranging from 3% to 12% [18]. During storage, trivalent chromium may oxidize to the more toxic hexavalent chromium, threatening water and soil safety [19]. Acid-base sludge, formed by mixing acid and alkaline residues from cold-rolling mills, is complex in composition, containing iron oxide, residual acids, and exhibiting both oiliness and alkaline corrosivity, thus posing significant environmental risks [20]. Ioana Monica Sur et al. [21]. conducted an ecological risk assessment of soils in the Baia Mare region, Romania. Their results revealed elevated levels of heavy metals in the topsoil (Cd:3.5–14.4 mg/kg; Cu:9.4–361.5 mg/kg; Pb:29.7–1973 mg/kg), with concentrations exceeding the limits set by Romanian legislation. Gianina Elena Damian et al. [22]. examined the effect of pH in humic acid washing solutions on removing lead and copper from a polymetallic contaminated soil. Using an in situ soil washing technique on soil from near the “Lăcădețu” mine in Zlatna, Romania, they assessed solutions at pH 3.0, 7.0, and 9.6 (the natural pH of the solution). The results demonstrated optimal removal efficiency under alkaline conditions, with 60.3% of copper and 48.08% of lead removed. The complex composition and high pollution risks of metallurgical solid waste necessitate urgent scientific analysis and assessment of its composition and potential hazards.

This study focuses on four representative types of metallurgical solid waste—jarosite slag (SW1), electric arc furnace dust (SW2), chromium-containing sludge (SW3) and acid-base sludge (SW4)—with the primary objectives of determining the speciation, leaching behavior, and potential health risks of heavy metals present in these wastes. By systematically evaluating their environmental release potential and toxic effects, this research aims to provide a scientific basis for the precise management and environmentally sound treatment of metallurgical solid wastes. An integrated analytical approach was employed. (1) X-ray fluorescence (XRF), X-ray diffraction (XRD), X-ray photoelectron spectroscopy (XPS), scanning electron microscopy (SEM), thermogravimetric analysis (TGA), and transmission electron microscopy (TEM). These techniques were used to comprehensively characterize the chemical composition, crystalline phases, and microstructural properties of the wastes, establishing a physicochemical foundation for interpreting their environmental behavior. (2) the BCR sequential extraction procedure was applied to assess the mobility and release characteristics of heavy metals. (3) The Risk Assessment Code (RAC) and the Overall Pollution Toxicity Index (OPTI) were further utilized to quantitatively evaluate potential threats to ecosystems and human health. This study is expected to deliver critical data for assessing the environmental impact and health risks associated with metallurgical solid wastes. The findings will support the optimization of waste disposal strategies and contribute to promoting green and sustainable development within the metallurgical industry.

## 2. Materials and Methods

### 2.1. Experimental Materials

Jarosite slag (SW1) is produced during the removal of iron from zinc, copper, nickel, and other non-ferrous metals in the wet smelting process using the iron alum method at the Chizhou Jiuhua smelter. Electric arc furnace ash (SW2), chromium-containing sludge (SW3), and acid-base sludge (SW4) are all byproducts of the metallurgical processes in the iron and steel industry, sourced from the Maanshan Masteel Group. Immediately after collection, the samples were placed in sealed polyethylene bags and refrigerated at 4 °C. For sample representativeness, a composite sampling approach was employed: within 24 h, 10 subsamples (approximately 1 kg each) were randomly collected at different time points from the discharge stream of each waste. After all subsamples were thoroughly mixed, they were reduced to approximately 2 kg using the quartering method for subsequent experimental analysis. Prior to analysis, all samples were dried to a constant weight in an oven at 60 °C, ground, and sieved through a 100-mesh nylon sieve to ensure sample homogeneity.

### 2.2. Characterization Methods

The particle size distribution of the samples was measured using a Mastersizer 2000 (Malvern Instruments Ltd. Malvern, Worcestershire, UK). The chemical composition of the samples was analyzed by X-ray fluorescence spectroscopy (XRF, EAGLE III model, EDAX Corporation, Warrendale, PA, USA). SEM (Hitachi SU8020, Tokyo, Japan) images were acquired on a Sirion 200 (FEI, Hillsboro, OR, USA) operated at an accelerating voltage of 15 kV and a working distance of ≈10 mm. Secondary-electron (SE) and backscattered-electron (BSE) detectors were used as appropriate. EDS spectra were collected with an Oxford INCA system (live time 60 s) and quantified using ZAF correction. Representative micrographs are taken within the magnification range of ×500 to ×10,000. XRD (DX-2700,Dandong Haoyuan Instrument Co., Ltd., Dandong, China) analysis was performed using a PANalytical BV X’Pert Pro diffractometer (Cu Kα, λ = 1.5406 Å) at 40 kV and 40 mA, scanned over 10–80° 2θ with a rate of 5°/min. Phase identification was conducted using HighScore Plus 4.9 and the ICDD PDF-4 database. XPS data were acquired with an ESCALAB 250Xi (Thermo Fisher, Waltham, MA, USA) using Al Kα radiation and processed with CasaXPS 2.3.25 after Shirley background subtraction. XRF data were analyzed using EDAX EAGLE III equipped with GENESIS software (GENESIS V7.0). ICP-OES (Agilent 5110, Santa Clara, CA, USA) and ICP-MS (PerkinElmer Nexion 1000, Waltham, MA, USA) were operated under standard plasma conditions, with quantification based on certified reference materials (GBW07405, GBW07406, Guangzhou, China). Thermal behavior was investigated using a STA 449 F3 Netzsch Thermogravimetric Analyzer (TGA, Selb, Germany) with a heating rate of 15 °C/min in air or nitrogen (N2). The total elemental composition of the acid-dissolved samples was determined using a Nexion 1000 Inductively Coupled Plasma Mass Spectrometer (ICP-MS, PerkinElmer, MA, USA), as well as an Agilent 5110 Inductively Coupled Plasma Optical Emission Spectrometer (ICP-OES, Agilent, CA, USA) and an AFS-933 Atomic Fluorescence Spectrometer (Jitian Instrument Co., Ltd. Beijing, China). The recovery rates of all elements ranged from 90% to 110%, with relative standard deviations (RSDs) less than 5%. Finally, the treated sample mixtures were analyzed using the sulfuric-nitric acid digestion method to assess metal leachability, followed by leaching tests.

### 2.3. Experimental Methods

The moisture content was determined by drying the samples at 105 °C for over 12 h and recording the mass before and after drying. The leaching potential of heavy metals in solid waste was evaluated using the HJ/T 300-2007 [23] method, as proposed by the Ministry of Ecology and Environment of the People’s Republic of China [24]. The four solid waste sample (SW1, SW2, SW3, SW4) is prepared to pass through a 5 mm sieve, and its moisture content is determined. A defined mass of the sample is placed in an extraction vessel and mixed with an extraction agent, a dilute acid solution of sulfuric and nitric acids with an initial pH of 3.20, at a liquid-to-solid ratio of 10:1. The sealed vessel is agitated for 18 h at 30 rpm using a rotary oscillator at room temperature. Following agitation, the mixture is pressure-filtered, and the resulting leachate is collected, preserved as necessary, and analyzed for target contaminant concentrations to assess leaching toxicity. The chemical speciation of heavy metals was analyzed using the standardized four-step BCR sequential extraction procedure [25]. The fractions were defined as follows: F1 (acid-soluble/exchangeable), extracted using 0.11 mol/L CH_3_COOH for 16 h; F2 (reducible), extracted with 0.5 mol/L NH_2_OH·HCl for 16 h; F3 (oxidizable), treated with 8.8 mol/L H_2_O_2_ at 85 °C followed by 1 mol/L NH_4_OAc for 16 h; and F4 (residual), determined after complete digestion using HNO_3_–HF–HClO_4_. Each extraction step was followed by centrifugation at 4000 rpm for 20 min and filtration through a 0.45 μm membrane prior to ICP-OES analysis. Chloride ion concentration was determined using a chloride ion-selective electrode. Standard solutions of varying concentrations were prepared, and their current values were measured to generate a standard curve.

### 2.4. Environmental Risk Assessment Method

To quantitatively assess the environmental risks posed by heavy metals in four types of metallurgical solid waste, this study employed two computational models: the Risk Assessment Code (RAC) and the Overall Pollution and Toxicity Index (OPTI). These methodologies enable quantitative analysis of heavy metals’ bioavailability and comprehensive toxicity, based on their chemical forms and leaching characteristics, thereby providing a scientific basis for risk classification. The RAC index enables the quantification of environmental risk by calculating the ratio between the bioavailability score of heavy metals and their total concentration. According to previous studies [26], the bioavailability score is defined as the *F*_1_ fraction obtained from the BCR sequential extraction procedure. The equations are as follows:

(1) $${RAC}_{BCR}=\frac{F_{1}}{F_{1}+{F_{2}+F_{3}+F}_{4}}$$

where *F*_1_: weak acid extractable state; *F*_2_: residual state; *F*_3_: reducible state; *F*_4_: oxidizable state.

To assess the overall environmental risk of multiple heavy metals in four samples under specific leaching conditions, the Overall Pollution Toxicity Index (OPTI) was also employed. This index considers the types of heavy metals, their toxicity, and stability, as well as their impact on environmental risk.

(2) $$OPTI=\sum_{1}^{M}T_{K}C_{K}^{I}L_{K}$$

where *M*: the number of relevant heavy metals; *T_K_*: the toxicity response factor of the heavy metals; and *K*: the corresponding heavy metal; $C_{K}^{I}=C_{K}-C_{K}^{M}$ (where $C_{K}$ is the total content of heavy metals, mg/kg; $C_{K}^{M}$ denotes the background concentration of heavy metals in Chinese surface soils, mg/kg); $L_{K}$ and is the ratio of leachable content to total heavy metal concentration [27].

## 3. Results and Discussion

### 3.1. Chemical Component Analysis

As illustrated in Figure S1, the iron oxide (Fe_2_O_3_) content in SW1 reached 51.9%, followed by sulfur trioxide (SO_3_), which accounted for approximately 31.7%. Additionally, trace amounts of silicon dioxide (SiO_2_, 7.5%), sodium oxide (Na_2_O, 4.4%), and zinc oxide (ZnO, 2.2%) were also present. In comparison, the Fe_2_O_3_ content in SW2 is slightly lower than in SW1, at approximately 51.4%. However, SW2 contained a significant proportion of ZnO, which represented 41.1% of the total mass. The calcium oxide (CaO) content in SW3 reached 23.6%, followed by chromium trioxide (Cr_2_O_3_), Fe_2_O_3_, and magnesium oxide (MgO), which comprised 19.8%, 18.2%, and 13.9%, respectively. Figure S1 demonstrates that SW4 was predominantly composed of Fe_2_O_3_ (45.5%) and CaO (30.3%), with smaller quantities of MgO (10.0%), SiO_2_ (5.0%), and aluminum oxide (Al_2_O_3_ 4.3%).

The results indicate that SW1, SW2, and SW4 contain high levels of Fe. However, due to technical and economic constraints, iron slag is temporarily disposed of by stockpiling [28], which presents high presents a significant challenge for effective solid waste management. Furthermore, the presence of chromium in SW3 and the potential formation of toxic Cr^6+^ through the oxidation of Cr^3+^ pose a substantial risk to water bodies and soil quality [19].

### 3.2. Phase Composition Analysis

**Figure 1:**
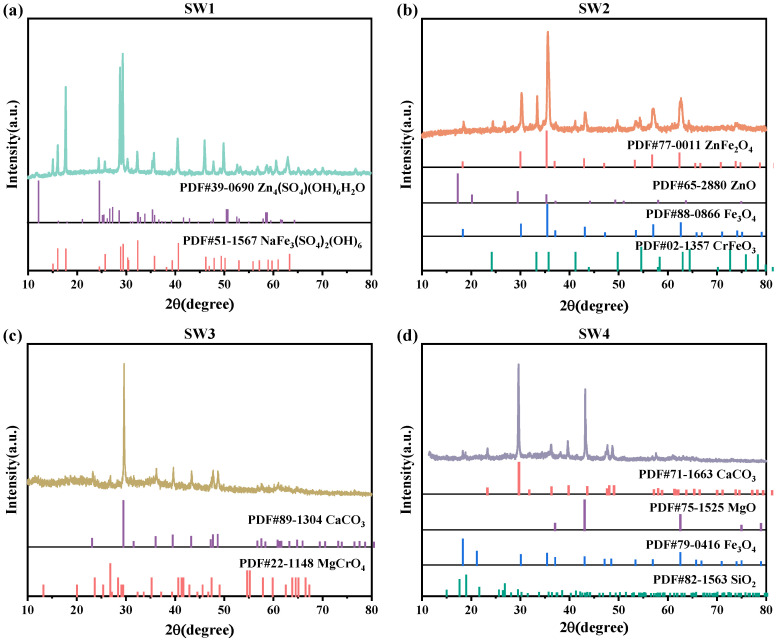
(**a**)The XRD pattern of jarosite slag (SW1), (**b**) The XRD pattern of electric arc furnace dust (SW2), (**c**) The XRD pattern of chromium-containing sludge (SW3) and (**d**) The XRD pattern of acid-base sludge (SW4).

The XRD spectra were baseline-corrected and smoothed using HighScore Plus 4.9, and the ICDD PDF-4 database. The characteristic diffraction peaks (2θ, °) of the dominant crystalline phases are summarized as follows: Zn_4_SO_4_(OH)_6_H_2_O (18.7°, 32.4°, 36.8°), NaFe_3_(SO_4_)_2_(OH)_6_ (15.1°, 30.3°, 37.6°) in SW1; Fe_3_O_4_ (30.1°, 35.5°, 57.0°), ZnFe_2_O_4_ (33.0°, 36.1°, 62.5°), and ZnO (31.8°, 34.4°, 47.6°) in SW2; CaCO_3_ (29.4°, 36.0°, 43.1°) and MgCrO_4_ (21.1°, 35.3°, 42.8°) in SW3; and Fe_3_O_4_ (30.1°, 35.5°, 57.0°), MgO (42.9°, 62.3°), and SiO_2_ (26.6°, 50.1°) in SW4. The XRD pattern of SW1 revealed that the slag was primarily composed of Zn_4_SO_4_(OH)_6_H_2_O and NaFe_3_(SO_4_)_2_(OH)_6_ phases (Figure 1a). Among these, Zn_4_SO_4_(OH)_6_H_2_O is a loosely bound hydroxy zinc sulfate hydrate that forms on the surface of Zn. As shown in Figure 1b, the physical phase of SW2 was predominantly characterized by Fe_3_O_4_, ZnO, and ZnFe_2_O_4_. Figure 1c shows that SW3 contained calcium carbonate (CaCO_3_) and magnesium chromate (MgCrO_4_). In contrast, the XRD pattern of SW4 (Figure 1d) identified the presence of Fe_3_O_4_, MgO, and SiO_2_ phases.

The high metal content in both SW1, SW2 and SW4 complicates their disposal, as improper handling could lead to environmental contamination. Therefore, resource recovery emerges as an effective strategy, not only reducing environmental risks but also maximizing economic benefits by extracting valuable metals [17,18]. Additionally, the presence of toxic Cr^6+^ in the MgCrO_4_ phase of SW3 highlights the need for stringent disposal measures [19]. Given the potential threat of chromium to aquatic ecosystems, careful consideration must be given to neutralizing or safely containing this compound to minimize its ecological impact.

### 3.3. Particle Size Distribution

**Figure 2:**
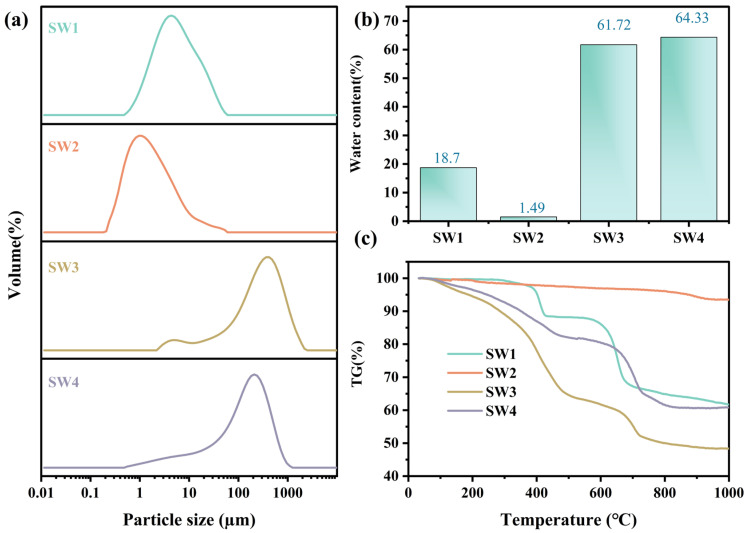
The particle size distribution (**a**), moisture content (**b**), and thermogravimetric of four solid wastes (**c**).

The particle size distribution for the four solid wastes (SW1, SW2, SW3, and SW4) are shown in Figure 2a and Table S1. The particle size distribution was analyzed using a Mastersizer 2000 (Malvern Instruments, Malvern, WorcestershireUK) equipped with a He–Ne laser (λ = 633 nm). The D_10_, D_50_, and D_90_ values were obtained using Mastersizer 2000 software. Notable variations in particle size and distribution patterns were observed. SW1 and SW2 exhibited finer particles, with D_50_ values of 4.76 µm and 1.34 µm, respectively, classifying them as fine particulate matter [29]. In contrast, SW3 and SW4 displayed coarser particles, with D_50_ values of 268.83 µm and 133.94 µm, respectively. At the same time, SW2 exhibited the broadest distribution (4.40), indicating a wider range of particle sizes, while SW3 and SW4 showed narrower distributions (Table S1).

Fine particulates (SW1 and SW2) have a higher tendency to adsorb heavy metals such as lead (Pb), cadmium (Cd), and copper (Cu), thereby enhancing the mobility and bioavailability of these metals in the environment [30]. In contrast, SW3 and SW4 are primarily composed of coarse particles, in which heavy metals are typically present in crystalline or mineral-bound forms with lower bioavailability [31]. However, upon deposition, these metals may still be released through weathering and acidification processes. Consequently, fine particulates such as SW1 and SW2 require greater attention in health risk assessments. For SW3 and SW4, the focus should shift to the long-term release of heavy metals in soil and water and their cumulative environmental impact.

### 3.4. Moisture Content and Thermogravimetric

As shown in Figure 2b, SW1 exhibited moderate moisture content (18.7%), which mitigates transportation challenges but increases the risk of heavy metal leaching during storage. SW2, with the lowest moisture content (1.49%), minimizes handling complexity. However, its fine particulate nature heightens the risk of airborne dispersion, necessitating solidification or stabilization [32,33]. In contrast, SW3 (61.72%) and SW4 (64.33%) exhibited high moisture contents, which not only elevated treatment costs but also exacerbated leachate risks. These characteristics underscore the need for advanced dehydration, stabilization, and resource recovery technologies to minimize environmental impacts and support sustainable waste management practices [34,35].

The thermogravimetric curves (Figure 2c) showed three distinct stages of mass loss. For SW1, 3.8% weight loss occurred below 150 °C due to moisture evaporation, followed by 7.2% between 400~600 °C attributed to decomposition of Zn_4_SO_4_(OH)_6_·H_2_O. SW2 exhibited negligible mass change (<1%) up to 800 °C, indicating high thermal stability. SW3 lost 12.5% mass below 200 °C (bound water) and 18.3% between 200~500 °C (decomposition of carbonates and hydroxides). SW4 exhibited a total loss of 16.8%, mainly between 100~600 °C due to dehydration and CaCO_3_ decomposition.

The weight loss of SW1 primarily occurred between 400 °C and 600 °C, with a substantial increase, indicating that its heavy metals may be present in relatively stable mineral forms. In contrast, SW2 exhibited negligible weight loss and demonstrated the highest thermal stability, which effectively immobilizes heavy metals and minimizes the risks associated with thermal treatment [36]. SW3, however, showed significant weight loss within the range of 100 °C to 500 °C, suggesting the presence of considerable amounts of bound water and thermally unstable compounds [37]. Similarly, the weight loss of SW4 predominantly occurred between 100 °C and 600 °C, although to a lesser extent than SW3. Nevertheless, its calcium-based compounds and adsorbed heavy metals may still be released at high temperatures, necessitating attention to their potential dissolution. Therefore, SW1 and SW2 exhibit superior thermal stability and a lower risk of heavy metal release, whereas SW3 and SW4 demonstrate reduced thermal stability, with SW3 requiring particular attention due to its pronounced risk of heavy metal release under high-temperature conditions. These findings provide critical insights and recommendations for the high-temperature treatment of metallurgical solid wastes.

In conclusion, the moisture content of metallurgical solid waste significantly affects the speciation and leaching characteristics of heavy metals. Fine particulates with low moisture content pose health risks through airborne dispersion, while coarse particulates with high moisture content are more likely to cause waterborne contamination. Therefore, in health risk assessments and solid waste management, strict pollution control measures should be implemented based on the moisture content and particle characteristics to effectively mitigate the environmental and health hazards associated with heavy metals.

### 3.5. Morphology Analysis

**Figure 3:**
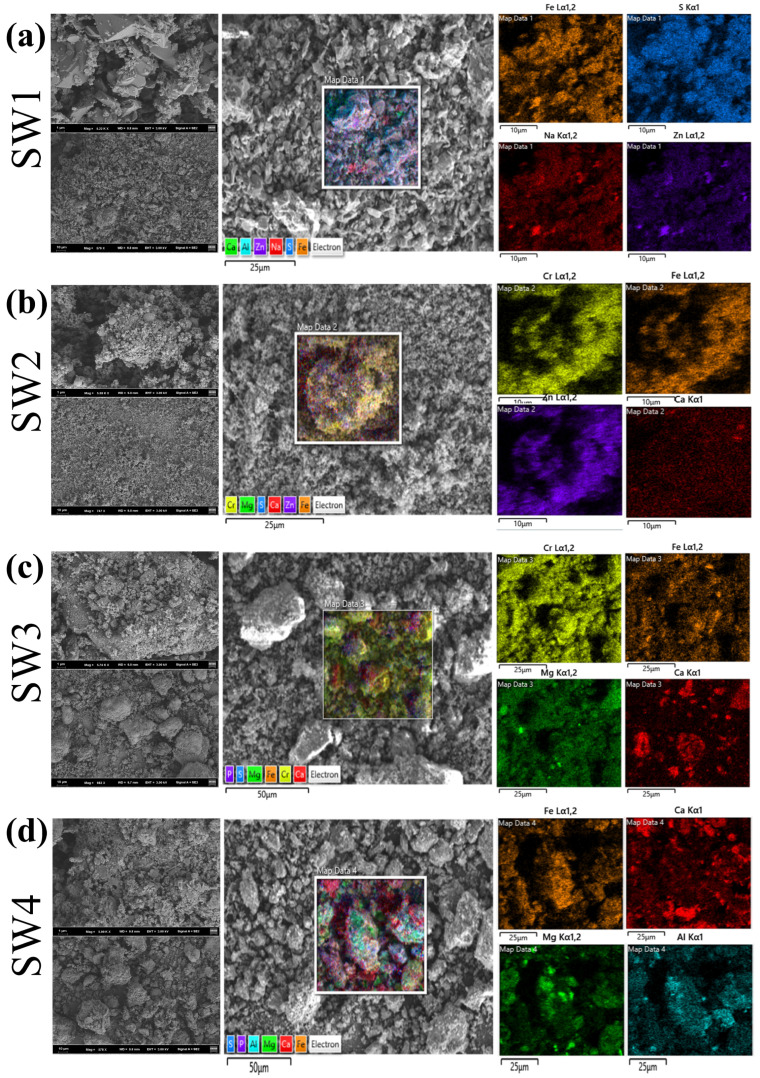
(**a**)The SEM of jarosite slag (SW1), (**b**) The SEM of electric arc furnace dust (SW2), (**c**) The SEM of chromium-containing sludge (SW3) and (**d**) The SEM of acid-base sludge (SW4).

As shown in Figure 3 and Figure S2, all SEM images were obtained at 15 kV with scale bars of 2 μm (SW1 and SW2) and 10 μm (SW3 and SW4). Particle size distributions measured by laser diffraction (Mastersizer 2000) are reported in Section 3.3; the D50 values were SW1 = 4.76 μm, SW2 = 1.34 μm, SW3 = 268.83 μm and SW4 = 133.94 μm. SEM observations qualitatively concur with these results: SW1 and SW2 show fine, flaky/porous particles while SW3 and SW4 exhibit coarser aggregated particles. SW1 primarily exhibited sheet-like and porous structures with rough surfaces, and EDS results indicated that Fe and S were the dominant elements (Tables S2 and S3), which consistent with the above XRD results showing that the main components are hydrated iron sulfate and hydrated zinc sulfate. This structure provides a high specific surface area, which may facilitate the enrichment of heavy metals within micropores [38]. However, under acidic or oxidizing conditions, heavy metals are prone to leaching [39]. SW2 displayed a particle-like and glassy structure, with Zn and Fe as the main elements (Tables S2 and S3). The glassy characteristics contribute to the stabilization of heavy metals, but cracks may serve as pathways for Zn release, particularly under acidic conditions, where Zn mobility poses a significant risk. SW3 was predominantly composed of flocculent aggregates that gradually formed clustered structures, with Cr and Fe as the main elements (Tables S2 and S3). The loose structure of this sample renders its heavy metal species highly soluble. Notably, the significant Cr content poses a high environmental and health risk due to the potential release of toxic Cr^6+^ under acidic or oxidizing conditions. SW4 exhibited a dense blocky structure primarily composed of Fe and Ca (Tables S2 and S3). XRD results shown that its main component is calcium carbonate. Alkaline conditions may enhance the passivation of heavy metals [40,41], but cracks and localized loose areas could lead to the release of heavy metals, especially under acidic conditions, where potential risks remain.

In summary, SW1 and SW3, due to their porous or loose structures, may exhibit higher risks of heavy metal release under specific conditions. Although SW2 contains relatively stable glassy materials, the effects of cracks should be closely monitored. The dense structure of SW4 reduces heavy metal release to some extent, but its long-term stability warrants further evaluation. These findings provide a scientific basis for the development of technologies for the harmless treatment of metallurgical solid wastes.

### 3.6. Element Valence Analysis

In the Fe2p spectrum, six peaks were identified: 711.6 and 725.3 eV for Fe^2+^, 713.6 and 727.2 eV for Fe^3+^ and 720.1 and 733.8 eV for satellite peaks, respectively. The S2p spectra showed peaks at 168.7 eV and 169.8 eV, corresponding to SO_4_^2−^2p_3/2_, and SO_4_^2−^ 2p_1/2_, respectively. For Zn, peaks at 1022.3 eV, 1039.7 eV, and 1045.3 eV were assigned to Zn^2+^ 2p_3/2_, loss feature, and Zn^2+^ 2p_1/2_, respectively. In the Mg1s spectrum, the peak at 1303.6 eV corresponded to Mg^2+^. Additionally, in presence of Mg, Mg KL_4_ region overlaps with the Ca2p region, resulting in the Ca2p spectrum being split into three components: Ca^2+^ 2p_3/2_ (347.4 eV), Ca^2+^ 2p_1/2_ (350.1 eV), and Mg KL_4_ (351.7 eV). The Cr2p spectra consisted of four peaks, attributed to Cr^3+^ 2p_3/2_ (575.3 eV), Cr^3+^ 2p_1/2_ (585.3 eV), Cr6^+^ 2p_3/2_ (578.1 eV), and Cr^6+^ 2p_1/2_ (587.1 eV). The XPS peak assignments were confirmed based on the NIST database. For example, Fe^2+^ (711.6 eV) and Fe^3+^ (713.6 eV) were identified in Fe 2p spectra, Zn^2+^ (1022.3 eV) in Zn 2p, and Cr^6+^ (578.1 eV) and Cr^3+^ (575.3 eV) in Cr 2p, consistent with corresponding XRD-identified phases such as Fe_3_O_4_, ZnFe_2_O_4_, and MgCrO_4_.

**Figure 4:**
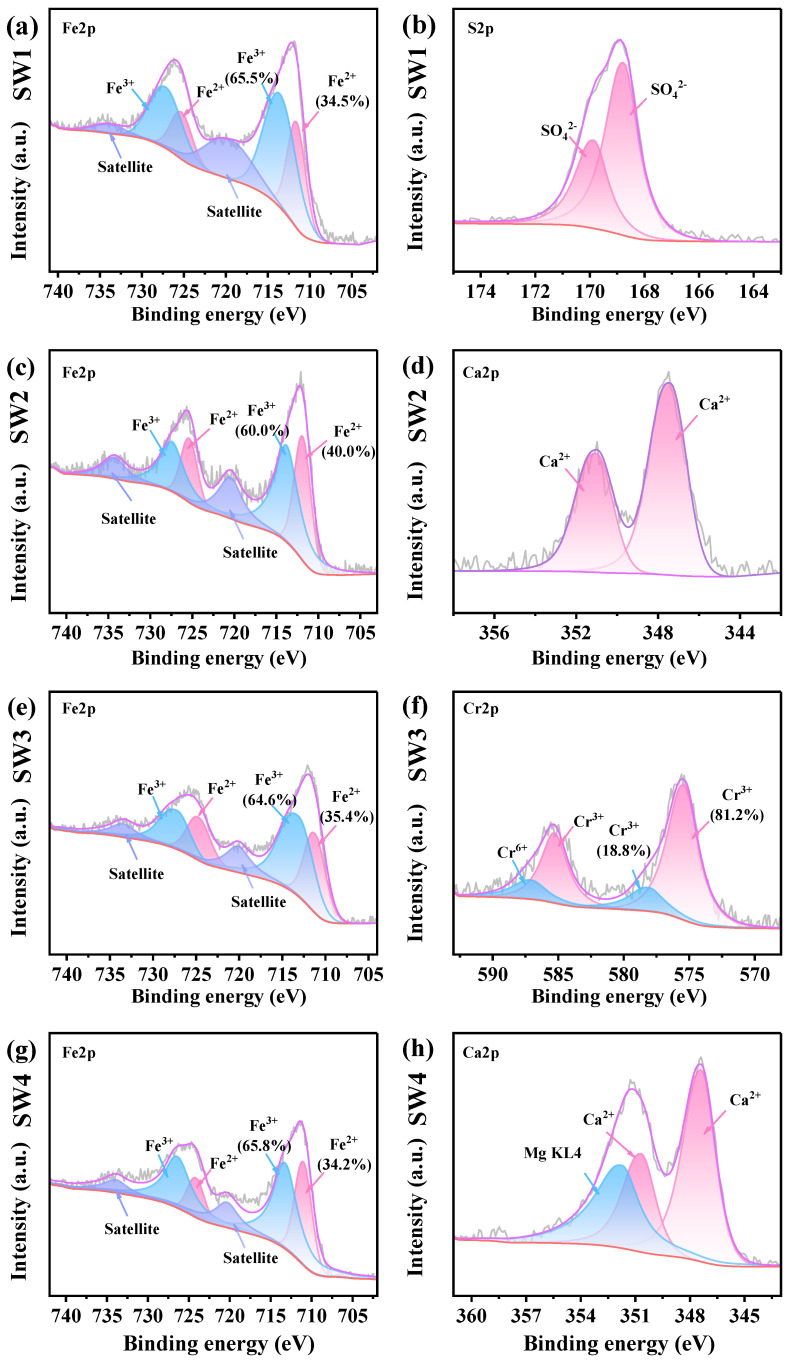
(**a**,**b**) The XPS spectrum ofjarosite slag (SW1), (**c**,**d**) The XPS spectrum of electric arc furnace dust (SW2), (**e**,**f**) The XPS spectrum of chromium-containing sludge (SW3) and (**g**,**h**) The XPS spectrum of acid-base sludge (SW4).

As illustrated in Figure 4b, the S2P spectra revealed the presence of SO_4_^2−^ in the SW1, which is consistent with the XRD and XRF results. The Zn2p spectra indicated Zn to be the form of Zn^2+^ in the SW1 and SW2 (Figure S3b,d). Figure 4c,d demonstrates that Mg is identified as Mg^2+^ in SW3 and SW4 (Figure S3f,h). This is consistent with the XRD results as well. Additionally, the content of Fe^3+^ in the four solid wastes is higher than that of Fe^2+^. In the management of solid waste, proper management and disposal methods must be implemented to prevent the indiscriminate dumping of these solid wastes [9]. Moreover, Ca^2+^ was detected in both SW2 and SW4 (Figure 4d,h), and based on XRD and XRF analyses, the source of Ca^2+^ was identified as CaCO_3_. Notably, the Cr2p spectra indicated that 18.8% of the Cr was in the Cr^6+^ state (Figure 4h). According to the XRF analysis, the high toxicity of Cr^6+^ underscores the need for preventive measures to mitigate potential health risks associated with SW3 [42].

### 3.7. Mass Fraction Analysis of Heavy Metals and Cl, S in Solid Waste

Table S4 demonstrates the mass fractions of heavy metals and Cl and S in solid waste. The SW1 contained a high concentration of S (111,000 mg·kg^−1^), followed by Pb (2977 mg·kg^−1^), As (2136 mg·kg^−1^), and Cu (1808 mg·kg^−1^). This is because SW1 is primarily composed of sulfates, which is consistent with the XRD results. In contrast, the Cl content in SW2 reached 20,448.4 mg·kg^−1^, which can be reduced through acid-base washing and high-temperature calcination. The results indicated that both SW1 and SW2 had high levels of Pb, As, Cu, and Cd. Based on health risk assessment and solid waste disposal technologies, it is recommended to recycle heavy metals or stabilize them to reduce their bioavailability and decrease their mobility in the environment [43]. Furthermore, both Cl and S concentrations were relatively high in SW3 and SW4. The high content S may lead to the formation of acidic leachate when the S comes into contact with moisture, which can result in soil and water acidification, thereby negatively affecting plant growth and aquatic organisms [44]. Cl may seep into groundwater, contaminating water sources. Under sunlight and high-temperature conditions, it can form toxic chlorinated organic compounds, posing risks to ecosystems and human health [45]. In comparison to SW1 and SW2, the heavy metal concentrations in SW3 and SW4 are relatively lower. However, the potential impact on water environments and soils during solid waste disposal processes should still not be overlooked.

### 3.8. Heavy Metal Toxicity Leaching Test

The leaching behavior of four samples was investigated through acid leaching experiments. The results are shown in Table 1, except for Zn in SW1, all other heavy metal elements were below the thresholds specified in GB 5085.3-2007 [46] for hazardous waste identification. Therefore, the thresholds outlined in the Chinese Surface Water Quality Standard (GB 3838-2002) [47] (Table S6) and the Groundwater Quality Standard (GB/T14848-2017) [48] (Table S7) were used as the baseline to assess the concentration of hazardous substances in solid waste leachate [49]. The leaching amounts of Cu (11.81 mg·L^−1^) and Cd (1.66 mg·L^−1^) in SW1 and Cd (0.159 mg·L^−1^) in SW2 exceeded the surface water and groundwater quality standards. All other metals were below these standards. Additionally, the heavy metal concentrations in SW3 and SW4 were also lower than the surface water and groundwater quality standards, indicating that SW3 and SW4 pose a lower safety risk, while SW1 and SW2 present a higher safety risk. The elevated leaching of Zn in SW1 may be associated with the chemical form of Zn, which is consistent with the results of XRD analysis showing the presence and form of Zn in SW1. Zelin Xu et al. [50]. reported that the extractant specified in HJ/T 299-2007 [51] for simulating acid rain lowered the surface pH of solid matrices, which enhanced the release of metals/metalloids. Alicja Kicin’ska et al. [52]. demonstrated that Cd and Zn posed a greater leaching hazard than other metallic elements when evaluated using alternative leaching methods. This suggests that the greater leachability of Cd and Zn increases their potential environmental risk.

### 3.9. Occurrence State Analysis of Metal Elements

The chemical form and distribution of heavy metals in four samples were investigated (Figure S4 and Table S5). During the extraction process, the residual state is identified as the most stable, with minimal environmental risk. However, the weak acid extractable state is more easily mobilized, suggesting that these metals may be released upon changes in environmental pH [53]. Cd, Cu, Ni, Pb, and Ce in SW1 were predominantly in the residual state (>65%), indicating a low environmental risk. However, the weak acid extractable fraction of SW1 (ranging from 4% to 24%) should not be overlooked, as it might pose a potential risk of environmental contamination, warranting further attention and treatment. Unfortunately, SW2 exhibited the highest percentage of Cd in the weak acid extractable state (52%), significantly surpassing the other three samples, which indicated that the environmental risk of Cd in SW2 was the highest. However, Ni and Cu in SW2 were predominantly present in the residual state, resulting in a relatively low leaching risk. The weak acid extractable fraction of Cd in SW3 (45%) was only surpassed by SW2. Notably, the weak acid extractable fraction of Ni in SW4 (14%) was higher than that of the other metals in SW4. Meanwhile, the reducible and oxidizable states of heavy metals in SW3 and SW4, as well as those of Pb and Ce in SW2, were found to be high. However, metals in the reducible and oxidizable fractions possess potential for mobility and bioavailability. These metals may be released in response to changes in environmental conditions [54]. In summary, the environmental risks associated with the four samples are ranked as follows: SW2>SW3>SW1>SW4.

### 3.10. Environmental Risk Assessment

**Figure 5:**
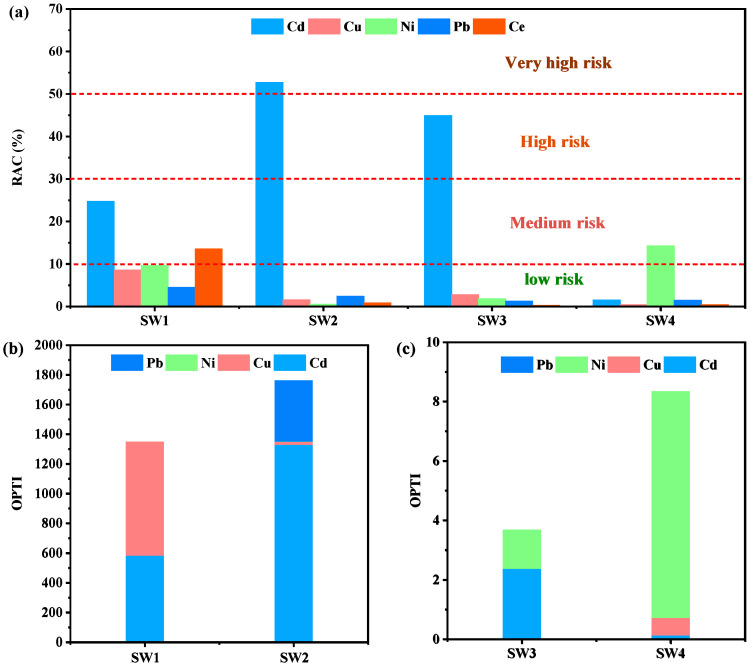
RAC of four solid wastes (**a**), OPTI of four solid wastes (**b**,**c**).

The environmental risk of heavy metals in the four samples can be assessed using the Risk Assessment Code (RAC). RAC classifies risks into five levels: values below 1% are considered safe for the environment, 1–10% indicates low risk, 11–30% represents moderate risk, 31–50% corresponds to high risk, and values exceeding 50% signify very high risk [55]. Based on the RAC calculation using the BCR method, Cd constitutes the primary environmental risk, reaching high-risk levels in SW2 and SW3, with RAC values ranging from 44.94% to 52.74%. Additionally, Ce in SW1 and Ni in SW4 exhibit moderate risk levels, while all other metals fall within the low-risk category (Figure 5a).

To evaluate the overall toxicity of heavy metals in the four samples, the Overall Pollution Toxicity Index (OPTI) was employed. Generally, SW3 and SW4 showed relatively low cumulative toxicity under acidic leaching conditions, whereas SW1 and SW2 exhibited higher toxicity (Figure 5b,c). Cd was the major contributor to the OPTI values in both SW1 and SW2, likely due to its cationic leaching behavior [56].

## 4. Conclusions

This study conducted a comprehensive investigation of four typical metallurgical solid wastes, yielding several significant findings:

Chemical composition analysis revealed that SW1, SW2, and SW4 contained high levels of Fe_2_O_3_ (45.5–51.9%), while SW3 showed significant amounts of CaO (23.6%) and Cr_2_O_3_ (19.8%). XRD and XPS analyses confirmed the presence of various metal-containing phases and different valence states of metals, particularly the concerning presence of Cr^6+^ (18.8%) in SW3. Physical characterization demonstrated distinct differences in particle size distribution and moisture content among the samples. SW1 and SW2 were characterized by fine particles (D_50_ < 5 μm) with relatively low moisture content, while SW3 and SW4 consisted of coarse particles (D_50_ > 100 μm) with high moisture content (>60%). These characteristics significantly influence their environmental behavior and treatment requirements.

The leaching toxicity tests revealed that SW1 and SW2 posed higher environmental risks, with several heavy metals exceeding standard limits. The BCR sequential extraction showed that Cd exhibited the highest mobility, particularly in SW2 and SW3, with weak acid extractable fractions of 52% and 45%, respectively. Environmental risk assessment using RAC and OPTI indicated that: Cd constituted the primary environmental risk, reaching high-risk levels in SW2 and SW3. The overall environmental risks ranked as: SW2>SW3>SW1>SW4. SW1 and SW2 demonstrated higher cumulative toxicity under acidic leaching conditions

These findings have important implications for the management and treatment of metallurgical solid wastes: Fine particulate wastes (SW1 and SW2) require careful handling and dust control measures due to their potential for airborne dispersion and higher heavy metal mobility. The high moisture content in SW3 and SW4 necessitates effective dewatering processes before further treatment or disposal. Special attention should be paid to Cd contamination, particularly in SW2 and SW3, where stabilization treatments may be necessary before disposal. The presence of Cr^6+^ in SW3 demands specific treatment strategies to prevent its release into the environment.

This research provides a scientific basis for optimizing waste management strategies and developing targeted treatment technologies for different types of metallurgical solid wastes, contributing to the sustainable development of the metallurgical industry.

## 5. Implications for Waste Management and Resource Recovery

The resource recovery potential of these wastes is explicitly discussed: Specifically, SW2, with its extremely high contents of ZnO (41.1%) and Fe_2_O_3_ (51.4%), is identified as a highly valuable secondary resource for zinc and iron recovery. Meanwhile, the challenge in its recycling is also noted, namely its relatively high chlorine content (20,448.4 mg/kg). Pretreatment processes such as washing or roasting may be required to remove chlorine prior to pyrometallurgical processing. For SW1 and SW4, their potential as iron recovery resources is also discussed, given their similarly high Fe_2_O_3_ contents (51.9% and 45.5%, respectively). Regarding SW3, the focus of its resource recovery is indicated to lie in the retrieval of chromium (Cr) and calcium (Ca), but the harmless treatment of highly toxic Cr(VI) must be prioritized.

## Figures and Tables

**Table 1:** Leaching toxicity of four solid wastes (ρ mg/L).

	SW1	SW2	SW3	SW4
Total Cr	0.12	0.1	0.04	<0.01
Cu	11.81	0.098	<0.01	0.07
Pb	<0.07	<0.07	<0.07	<0.07
Cd	1.66	0.159	<0.003	<0.003
Ni	0.02	<0.01	0.01	0.03
Ba	0.11	0.16	<0.002	0.008
Ag	<0.01	<0.01	<0.01	0.03
Cr(VI)	<0.004	<0.004	0.004	<0.004
As	0.03	<0.0001	<0.0001	<0.0001
Hg	0.00025	0.00431	<0.00002	<0.00002
Se	<0.0001	<0.0001	<0.0001	0.0002
Be	0.0226	<0.0007	<0.0007	<0.0007
F^−^	0.52	11.5	<0.1	0.32

Note: Red indicates that the concentration of the metal ion exceeds the standard value.

## Data Availability

The original contributions presented in this study are included in the article/Supplementary Material. Further inquiries can be directed to the corresponding author.

## Supplementary Materials

The following supporting information can be downloaded at: https://www.mdpi.com/article/10.3390/jox15060211/s1, Table S1: Particle size of four solid wastes. Table S2: The SEM EDS results of four solid wastes. Table S3: The TEM EDS results of four solid wastes. Table S4: Mass fraction of heavy metals and Cl, S in four solid wastes (ω mg·kg^−1^). Table S5: The results of the BCR sequential extraction procedure (mg/kg). Table S6: The Chinese National Standard for Surface Water Quality (GB 3838-2002) regarding selected heavy metals. Table S7: the Chinese National Standard for Groundwater Quality (GB/T14848-2017) regarding specific heavy metals. Figure S1: The chemical composition of four solid wastes. Figure S2: The TEM of four solid wastes. Figure S3: The XPS spectrum of four solid wastes. Figure S4: The occurrence state of metal elements in four solid wastes. File S1: Original images of Figure 3 and Figure S2.
