# Supplementary material for: Heavy Metals Environmental Fate in Metallurgical Solid Wastes: Occurrence, Leaching, and Ecological Risk Assessment
Source: J Xenobiot. 2025 Dec 15;15(6):211. doi: 10.3390/jox15060211 (PMC12733436; doi:10.3390/jox15060211)
Supplement: Supplementary file 1 [file jox-15-00211-s001.zip › FileS1-Original images of Figures 3 and S2/Figure3/Figure3a SW1/1-3 EDS.pdf]

Electron Image 3

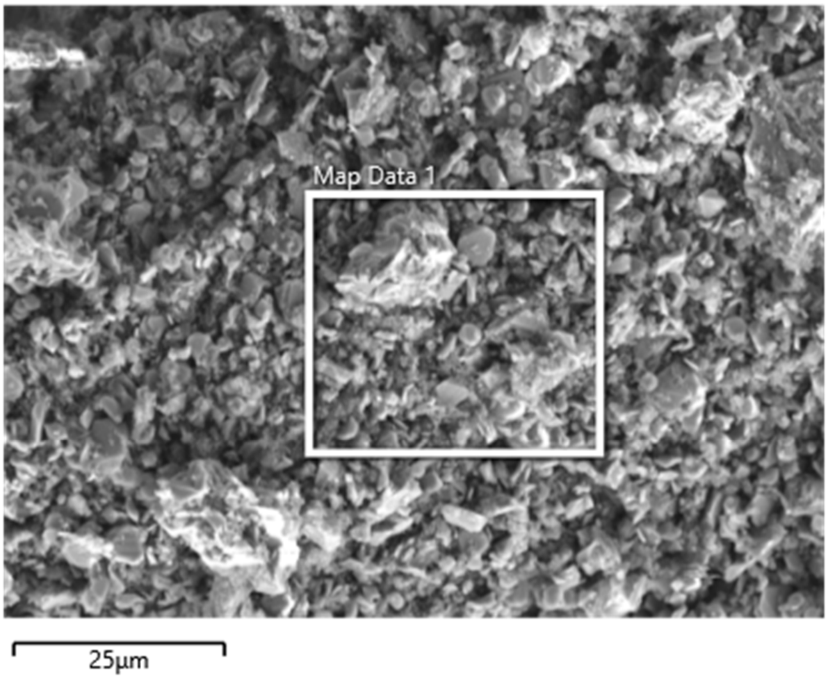

EDS Layered Image 1

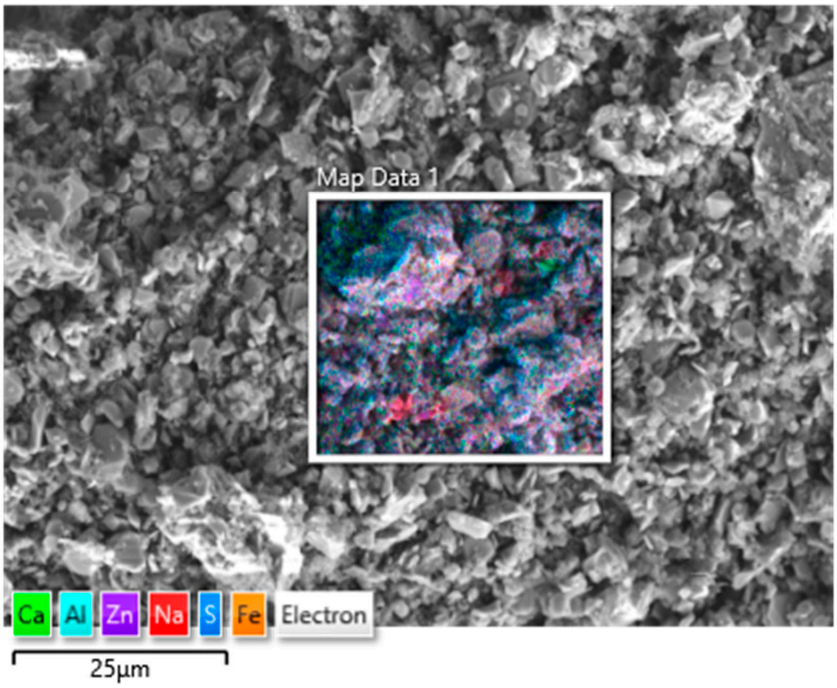

OXFORD INSTRUMENTS
The Business of Science®

S Kα1

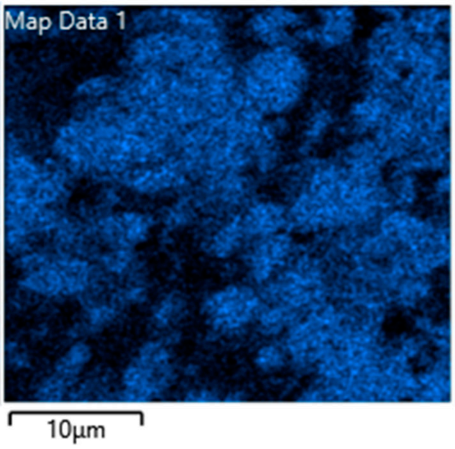

Fe Lα1,2

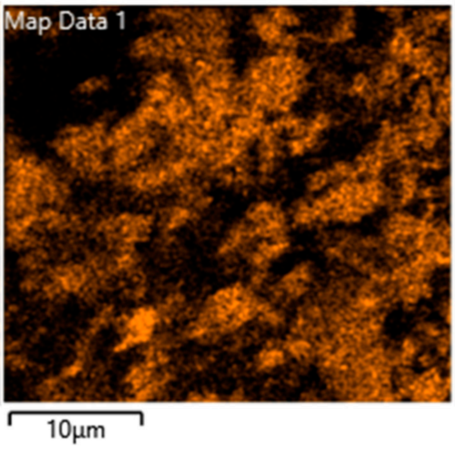

Na Kα1,2

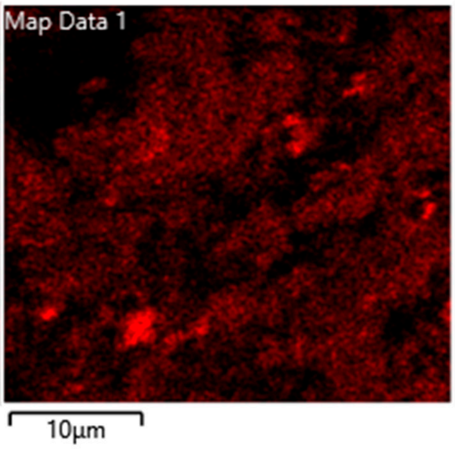

Zn Lα1,2

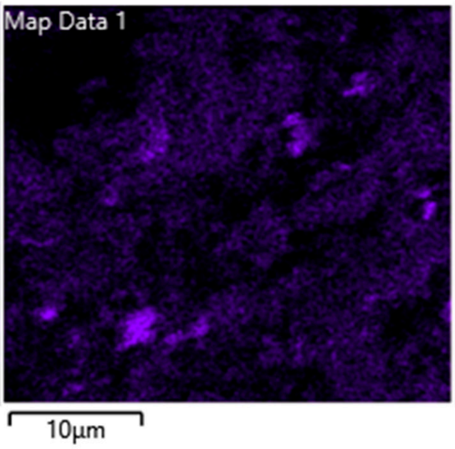

Al Kα1

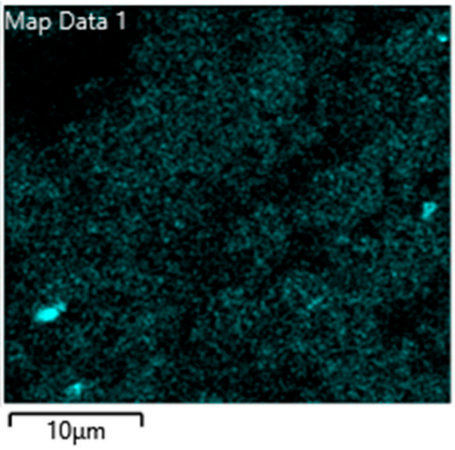

Ca Kα1

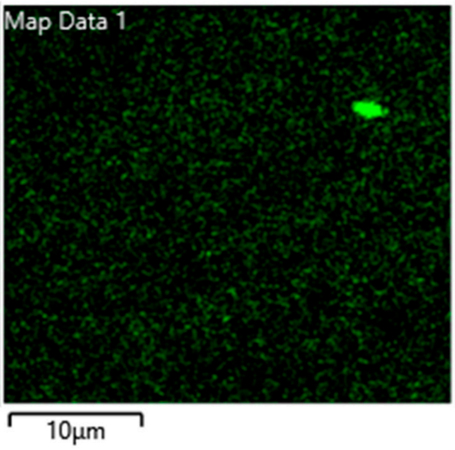

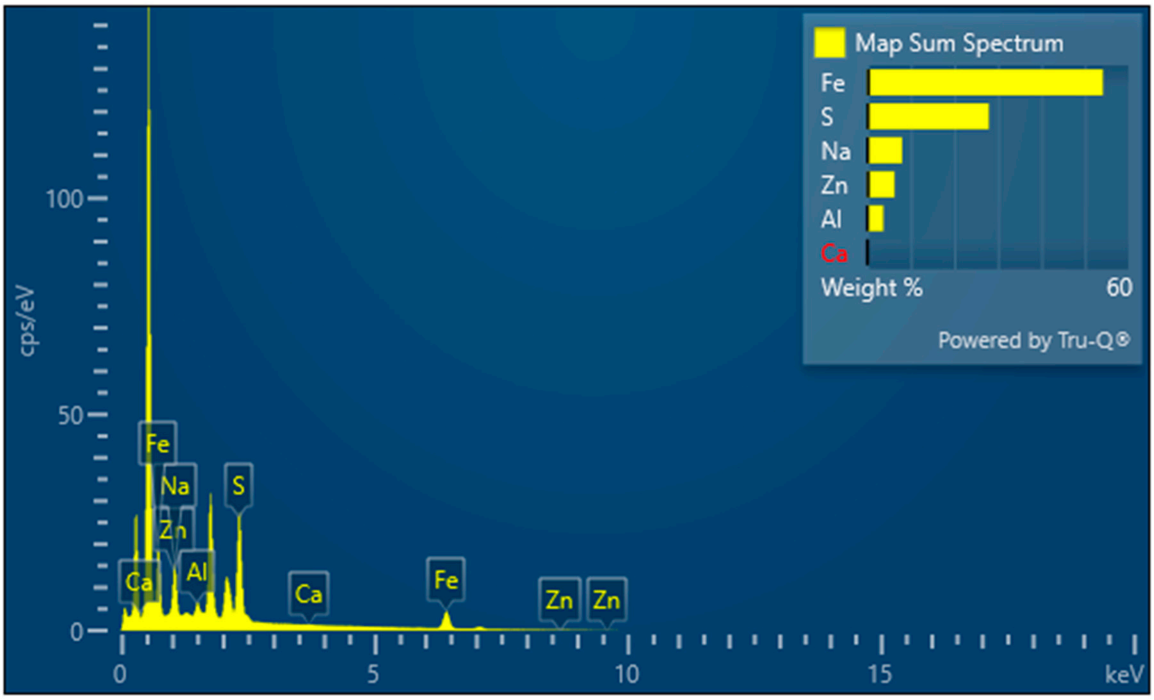

Map Sum Spectrum		
Element	Wt%	Wt% Sigma
Na	8.00	0.15
Al	3.69	0.07
S	27.94	0.20
Ca	0.00	0.09
Fe	54.10	0.30
Zn	6.27	0.26
Total:	100.00	

Map Sum Spectrum	Line Type	Apparent Concentration	k Ratio	Wt%	Wt% Sigma	Atomic %	Standard Label	Factory Standard	Standard Calibration Date
Na	K series	8.17	0.03448	8.00	0.15	14.37	Albite	Yes	
Al	K series	3.13	0.02247	3.69	0.07	5.66	Al2O3	Yes	
S	K series	25.42	0.21900	27.94	0.20	35.99	FeS2	Yes	
Ca	K series	0.00	0.00000	0.00	0.09	0.00	Wollastonite	Yes	
Fe	L serie	37.93	0.37930	54.10	0.30	40.02	Fe	Yes	

OXFORD INSTRUMENTS
The Business of Science®

	s							
Zn	L series	2.56	0.02559	6.27	0.26	3.96	Zn	Yes
Total				100.00		100.00		
