# Supplementary material for: Heavy Metals Environmental Fate in Metallurgical Solid Wastes: Occurrence, Leaching, and Ecological Risk Assessment
Source: J Xenobiot. 2025 Dec 15;15(6):211. doi: 10.3390/jox15060211 (PMC12733436; doi:10.3390/jox15060211)
Supplement: Supplementary file 1 [file jox-15-00211-s001.zip › FileS1-Original images of Figures 3 and S2/Figure3/Figure3b SW2/2-3 EDS.pdf]

Electron Image 4

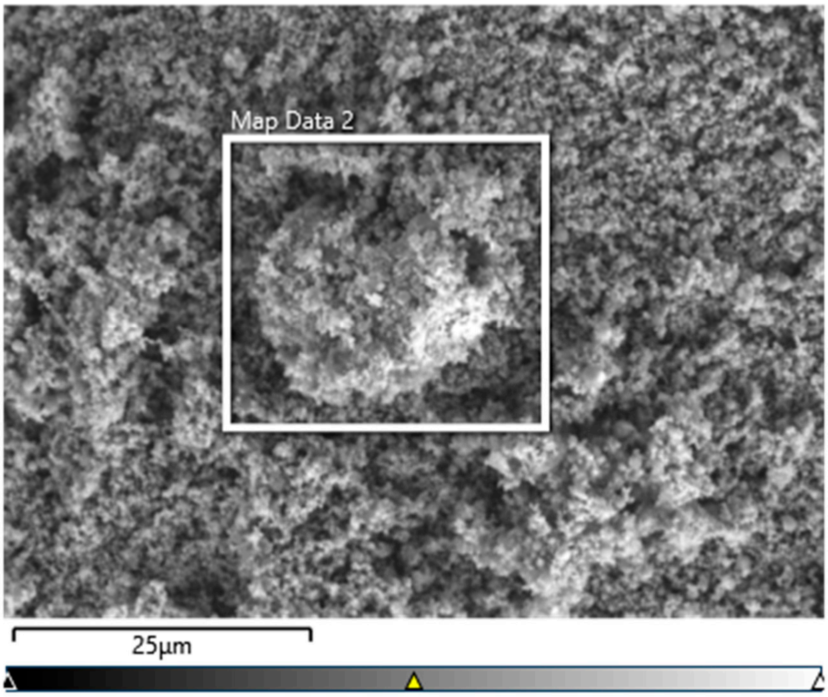

EDS Layered Image 2

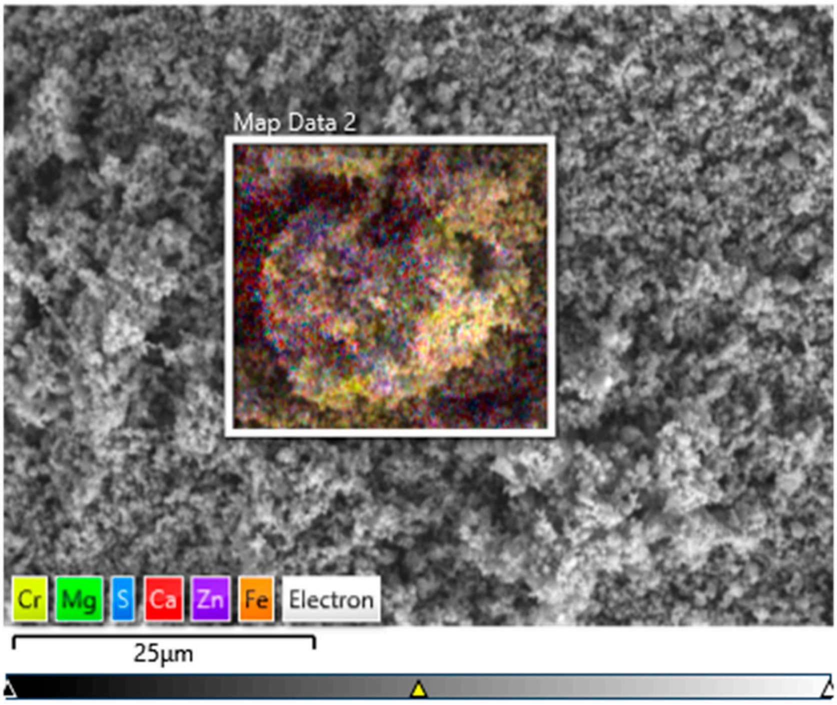

Zn Lα1,2

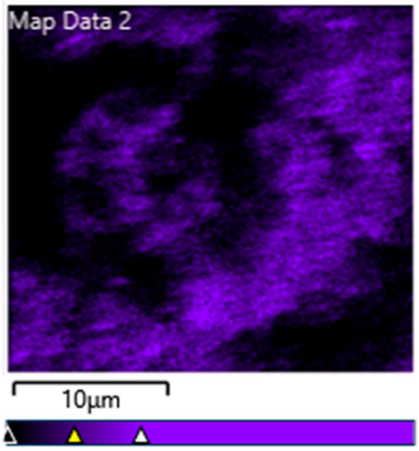

Cr Lα1,2

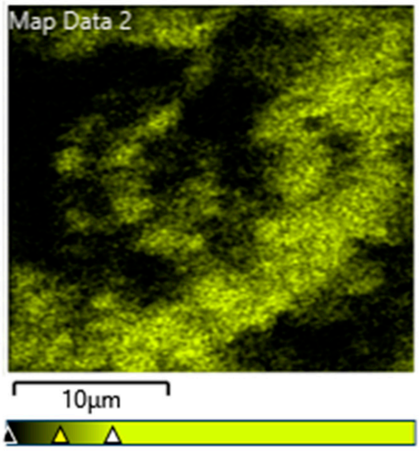

Fe Lα1,2

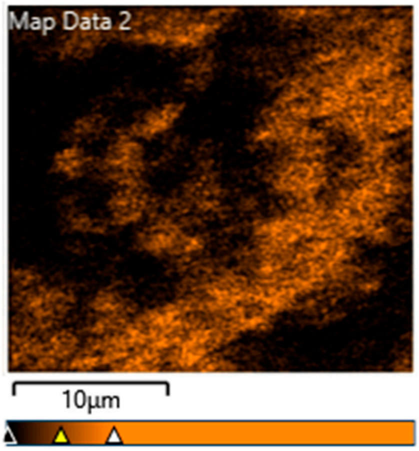

S Kα1

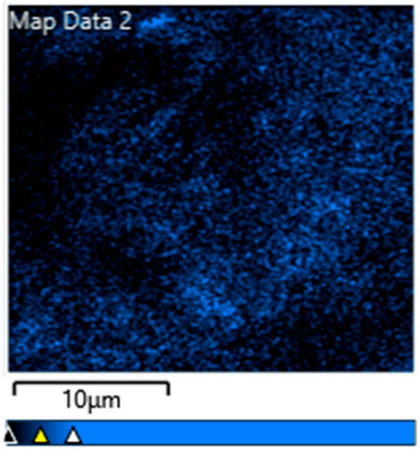

Ca Kα1

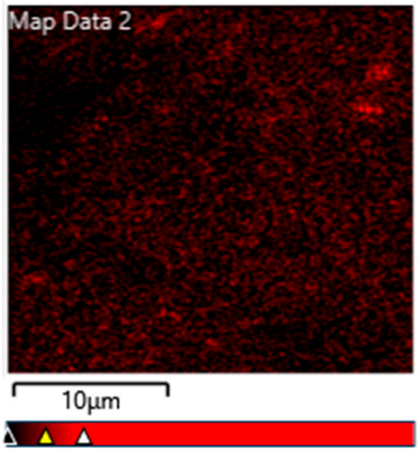

Mg Kα1,2

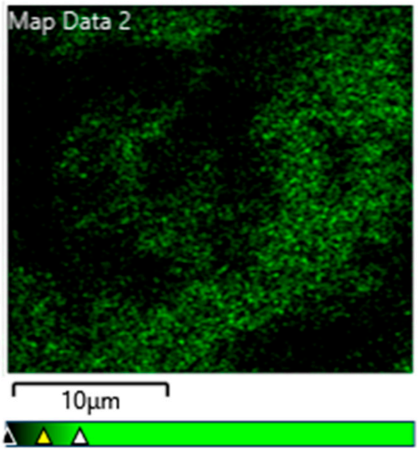

OXFORD INSTRUMENTS
The Business of Science®

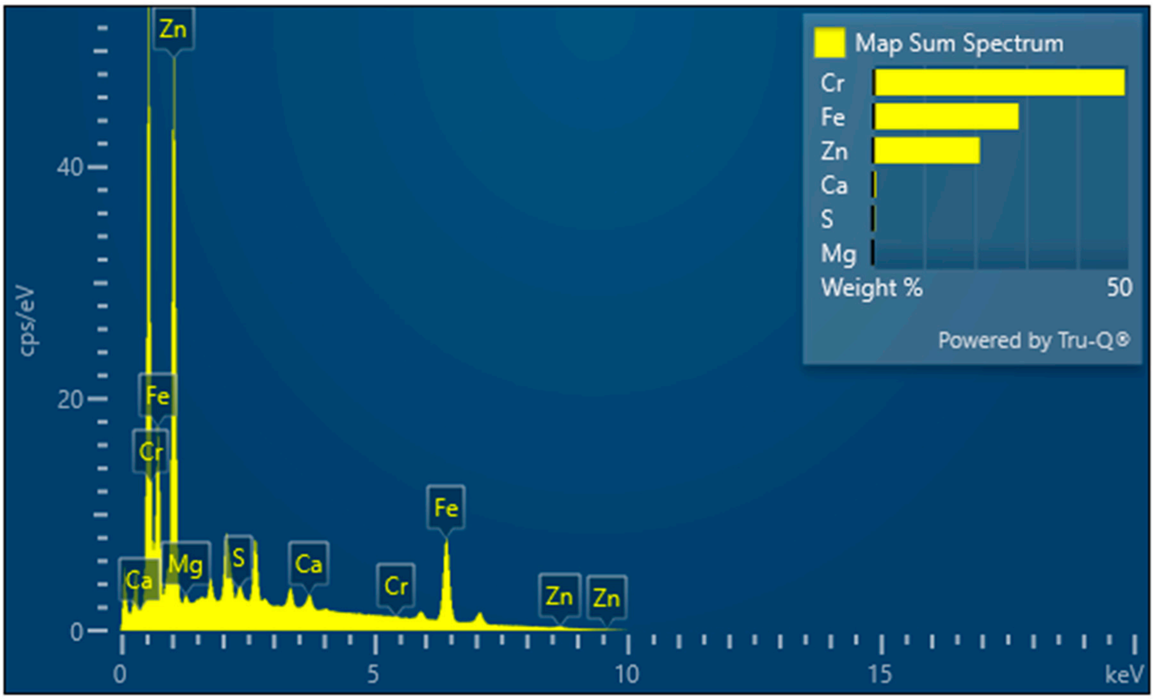

Map Sum Spectrum		
Element	Wt%	Wt% Sigma
Mg	0.25	0.02
S	0.41	0.02
Ca	0.66	0.03
Cr	49.27	0.23
Fe	28.54	0.21
Zn	20.87	0.12
Total:	100.00	

Map Sum Spectrum	Line Type	Apparent Concentration	k Ratio	Wt%	Wt% Sigma	Atomic %	Standard Label	Factory Standard	Standard Calibration Date
Mg	K series	0.94	0.00625	0.25	0.02	0.57	MgO	Yes	
S	K series	1.97	0.01693	0.41	0.02	0.71	FeS2	Yes	
Ca	K series	3.29	0.02942	0.66	0.03	0.90	Wollastonite	Yes	
Cr	L series	187.12	1.87123	49.27	0.23	52.13	Cr	Yes	
Fe	L serie	54.50	0.54495	28.54	0.21	28.12	Fe	Yes	

OXFORD INSTRUMENTS
The Business of Science®

	s							
Zn	L series	41.94	0.41939	20.87	0.12	17.57	Zn	Yes
Total				100.00		100.00		
