# Supplementary material for: Heavy Metals Environmental Fate in Metallurgical Solid Wastes: Occurrence, Leaching, and Ecological Risk Assessment
Source: J Xenobiot. 2025 Dec 15;15(6):211. doi: 10.3390/jox15060211 (PMC12733436; doi:10.3390/jox15060211)
Supplement: Supplementary file 1 [file jox-15-00211-s001.zip › FileS1-Original images of Figures 3 and S2/Figure3/Figure3c SW3/3-3 EDS.pdf]

Electron Image 5

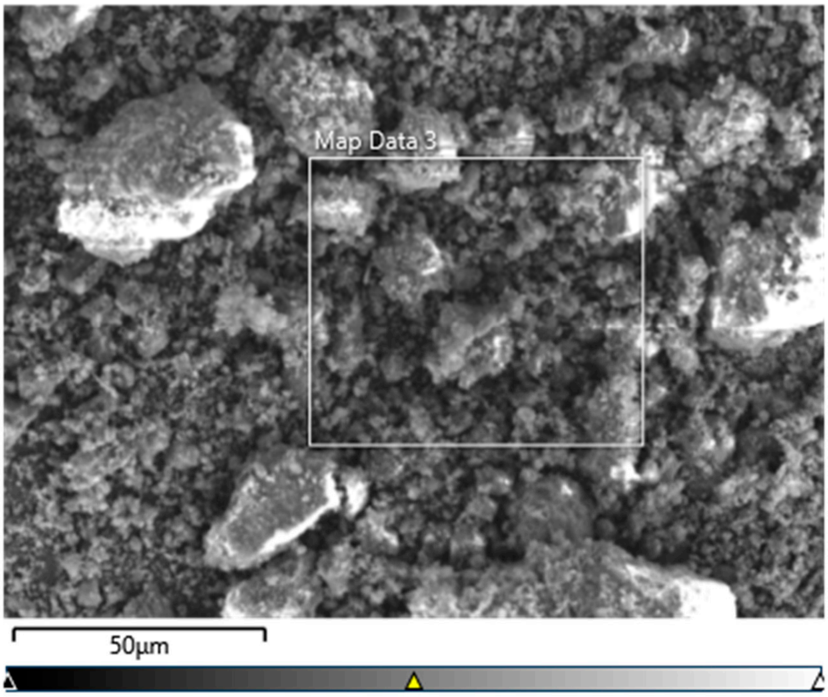

EDS Layered Image 3

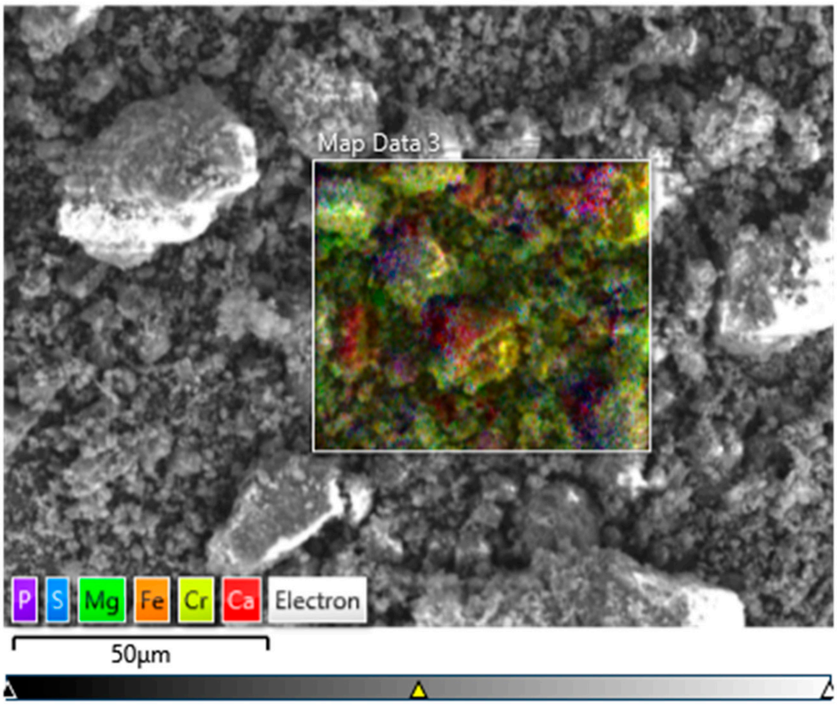

Cr Lα1,2

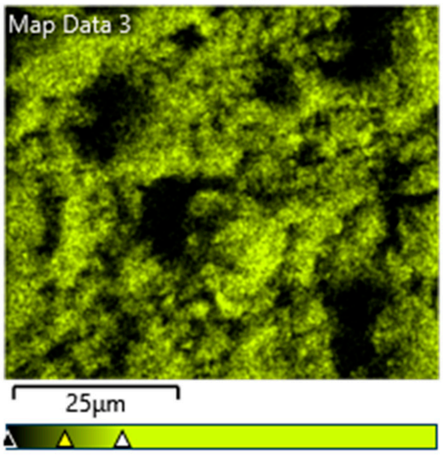

Mg Kα1,2

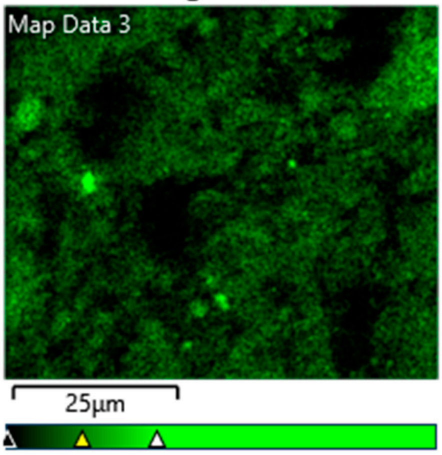

Ca Kα1

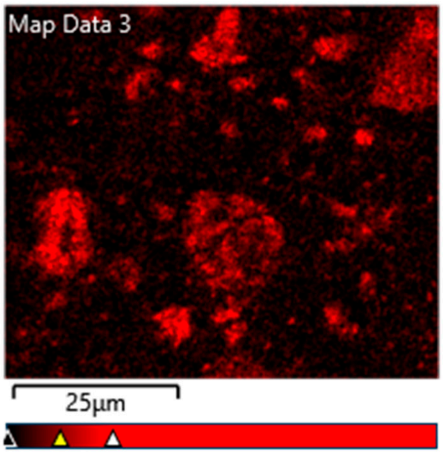

Fe Lα1,2

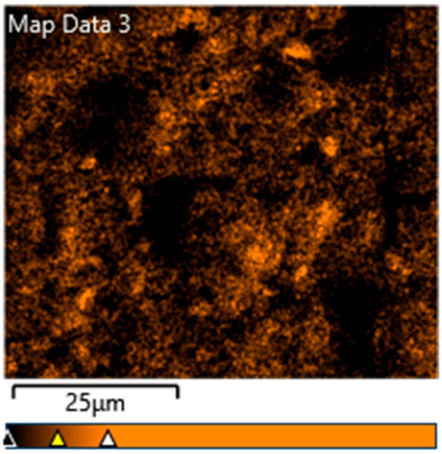

P Kα1

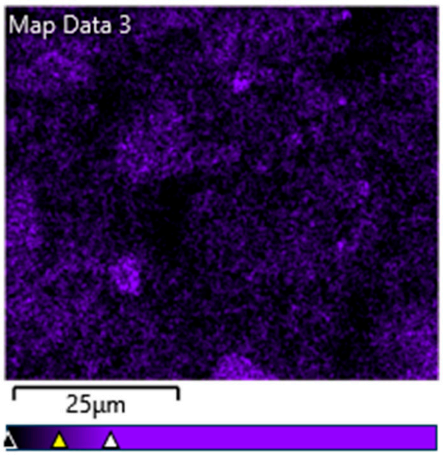

S Kα1

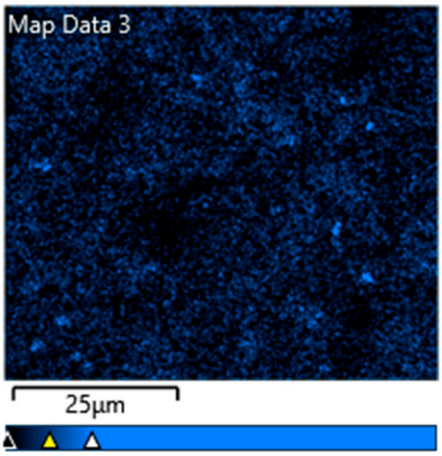

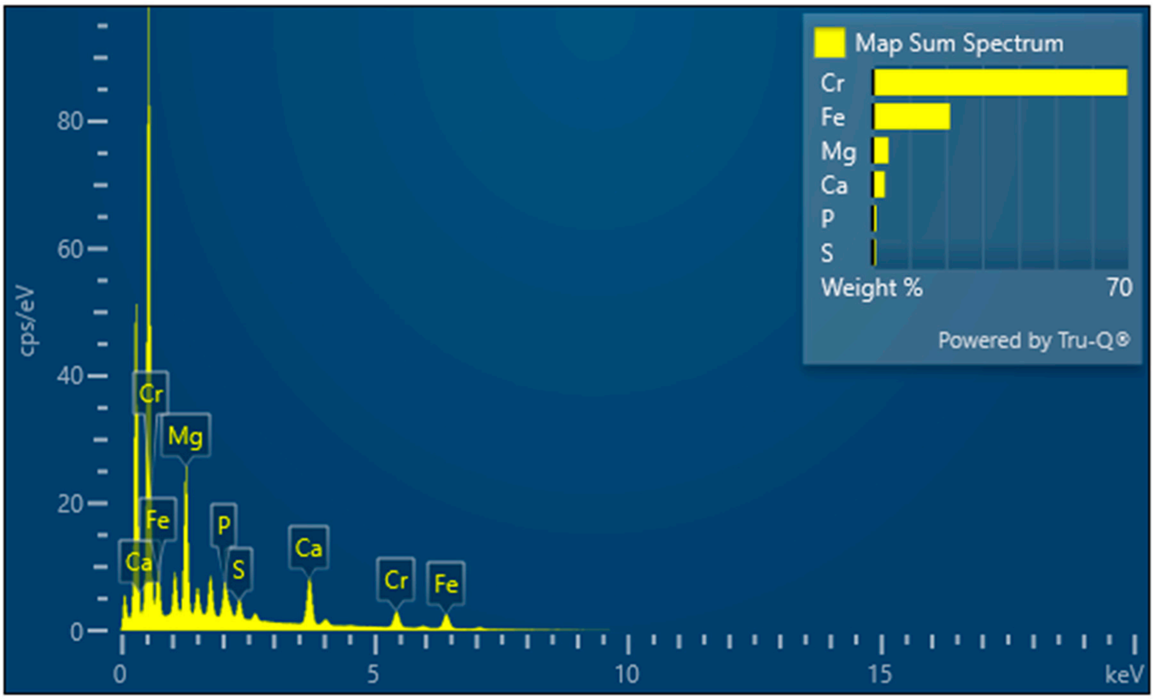

Map Sum Spectrum		
Element	Wt%	Wt% Sigma
Mg	4.33	0.03
P	0.94	0.02
S	0.73	0.01
Ca	3.30	0.03
Cr	69.61	0.17
Fe	21.08	0.17
Total:	100.00	

Map Sum Spectrum	Line Type	Apparent Concentration	k Ratio	Wt%	Wt% Sigma	Atomic %	Standard Label	Factory Standard	Standard Calibration Date
Mg	K series	21.55	0.14295	4.33	0.03	8.78	MgO	Yes	
P	K series	7.78	0.04353	0.94	0.02	1.49	GaP	Yes	
S	K series	4.25	0.03664	0.73	0.01	1.12	FeS2	Yes	
Ca	K series	19.74	0.17634	3.30	0.03	4.06	Wollastonite	Yes	
Cr	L serie	310.69	3.10687	69.61	0.17	65.95	Cr	Yes	

OXFORD INSTRUMENTS
The Business of Science®

	s							
Fe	L series	37.20	0.37204	21.08	0.17	18.59	Fe	Yes
Total				100.00		100.00		
