# Supplementary material for: Heavy Metals Environmental Fate in Metallurgical Solid Wastes: Occurrence, Leaching, and Ecological Risk Assessment
Source: J Xenobiot. 2025 Dec 15;15(6):211. doi: 10.3390/jox15060211 (PMC12733436; doi:10.3390/jox15060211)
Supplement: Supplementary file 1 [file jox-15-00211-s001.zip › FileS1-Original images of Figures 3 and S2/Figure3/Figure3d SW4/4-3 EDS.pdf]

Electron Image 7

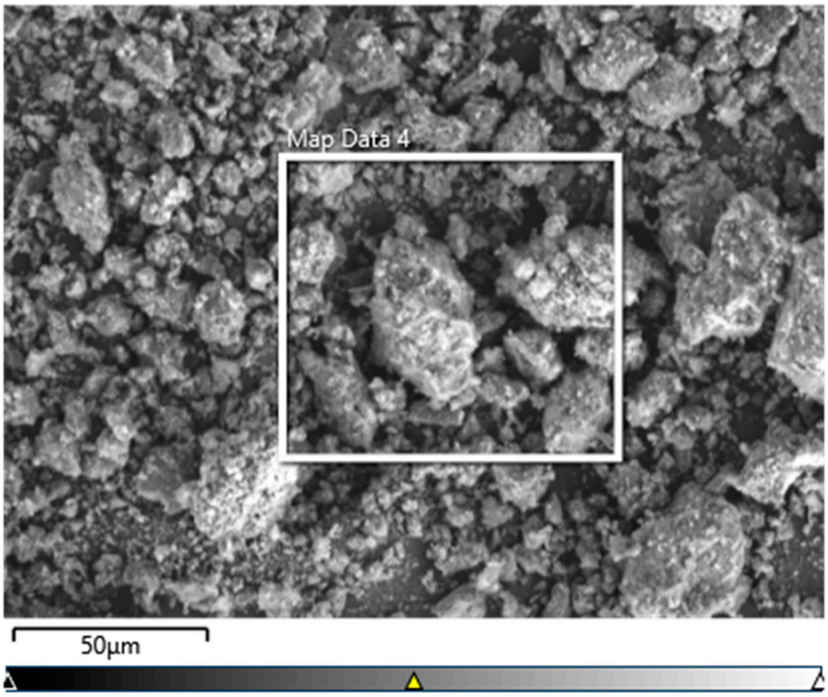

EDS Layered Image 4

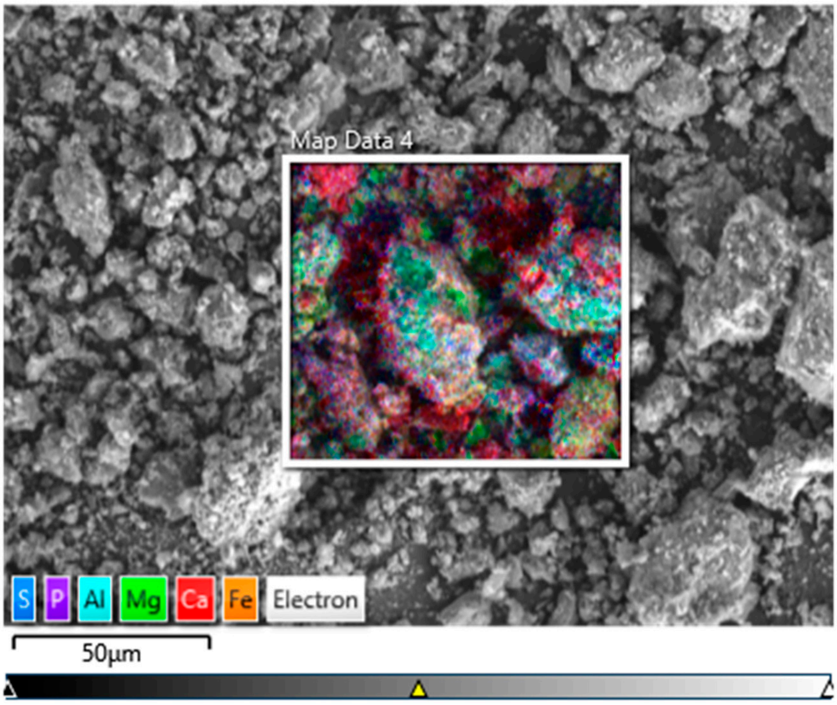

Mg Kα1,2

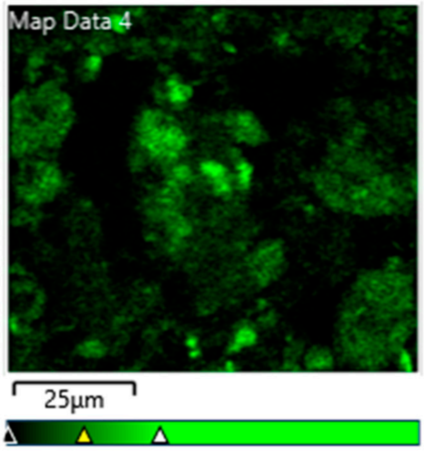

Ca Kα1

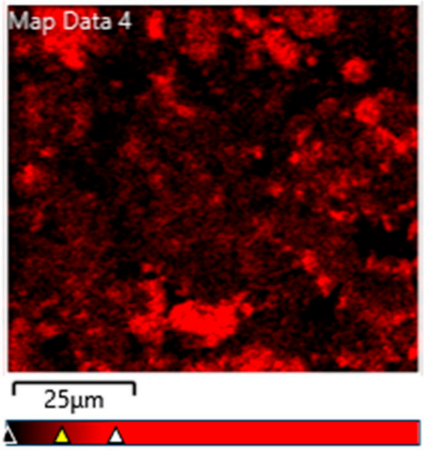

Fe Lα1,2

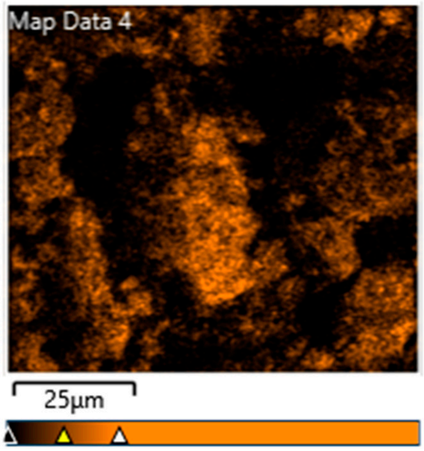

P Kα1

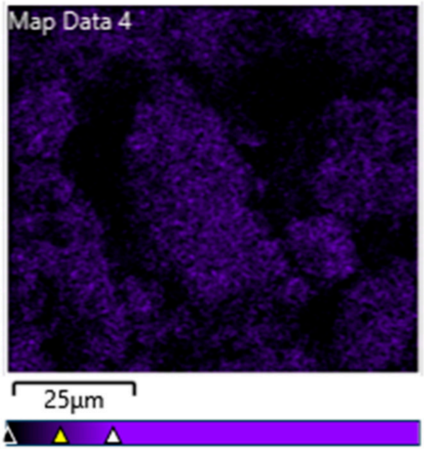

Al Kα1

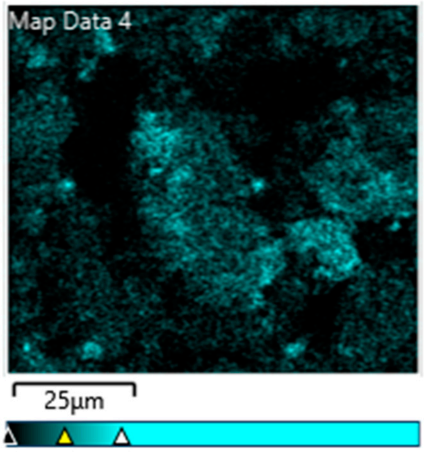

S Kα1

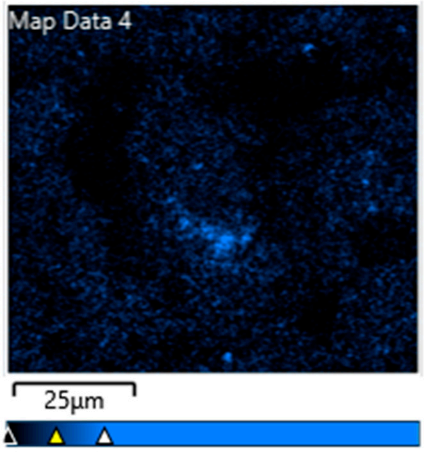

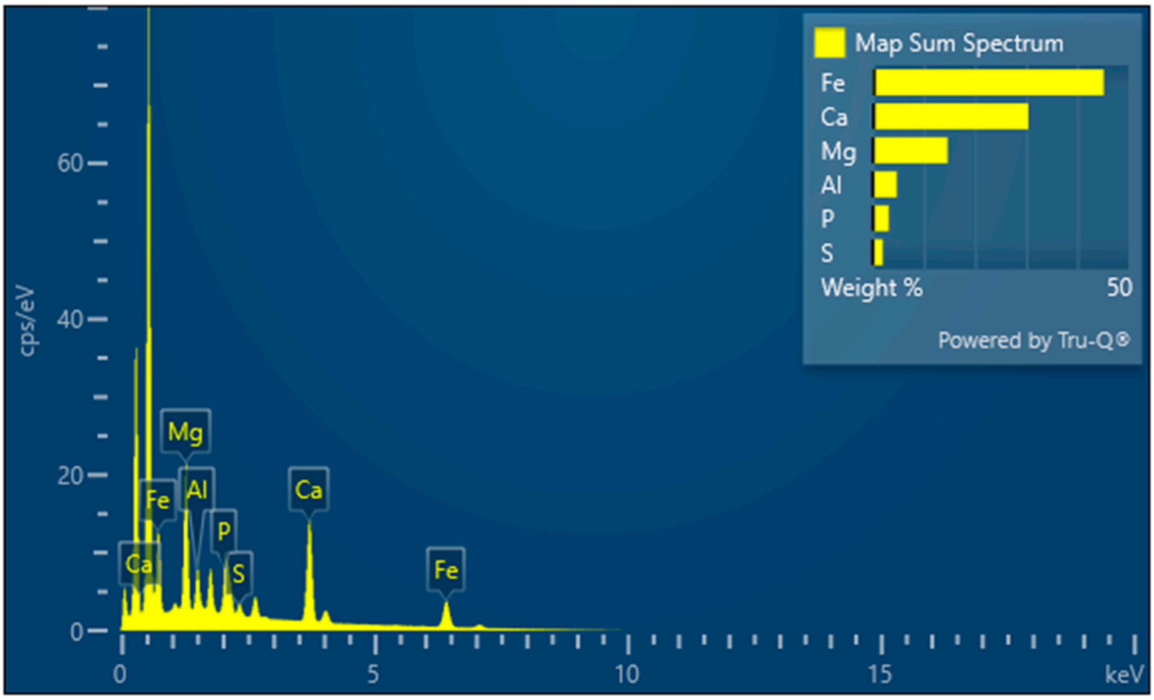

Map Sum Spectrum		
Element	Wt%	Wt% Sigma
Mg	14.63	0.11
Al	4.70	0.07
P	3.15	0.08
S	1.94	0.06
Ca	30.44	0.20
Fe	45.14	0.30
Total:	100.00	

Map Sum Spectrum	Line Type	Apparent Concentration	k Ratio	Wt%	Wt% Sigma	Atomic %	Standard Label	Factory Standard	Standard Calibration Date
Mg	K series	17.13	0.11360	14.63	0.11	24.01	MgO	Yes	
Al	K series	5.30	0.03805	4.70	0.07	6.95	Al2O3	Yes	
P	K series	5.45	0.03046	3.15	0.08	4.06	GaP	Yes	
S	K series	2.34	0.02013	1.94	0.06	2.41	FeS2	Yes	
Ca	K serie	37.59	0.33583	30.44	0.20	30.31	Wollastonite	Yes	

	s							
Fe	L series	33.86	0.33856	45.14	0.30	32.26	Fe	Yes
Total				100.00		100.00		

OXFORD INSTRUMENTS
The Business of Science®
