# Supplementary material for: Heavy Metals Environmental Fate in Metallurgical Solid Wastes: Occurrence, Leaching, and Ecological Risk Assessment
Source: J Xenobiot. 2025 Dec 15;15(6):211. doi: 10.3390/jox15060211 (PMC12733436; doi:10.3390/jox15060211)
Supplement: Supplementary file 1 [file jox-15-00211-s001.zip › jox-3898946-Supporting Information-proof-done.pdf]

Journal of
Xenobiotics

Supplementary Materials: Heavy Metals Environmental Fate in Metallurgical Solid Wastes: Occurrence, Leaching, and Ecological Risk Assessment

Shuqin Li and Guohua Ni

Tables:

Table S1 Particle size of four solid wastes.

Table S2 The SEM EDS results of four solid wastes.

Table S3 The TEM EDS results of four solid wastes.

Table S4 Mass fraction of heavy metals and Cl, S in four solid wastes (ω mg·kg^{-1}).

Table S5 The results of the BCR sequential extraction procedure (mg/kg).

Table S6 the Chinese National Standard for Surface Water Quality (GB 3838-2002) regarding selected heavy metals

Table S7 the Chinese National Standard for Groundwater Quality (GB/T14848-2017) regarding specific heavy metals

Figs:

Figure. S1. The chemical composition of four solid wastes.

Figure. S2. The TEM of four solid wastes.

Figure. S3. The XPS spectrum of four solid wastes.

Figure. S4. The occurrence state of metal elements in four solid wastes.

Table S1 Particle size of four solid wastes.

Solid waste	SW1	SW2	SW3	SW3
D_{10}	1.47	0.44	30.81	9.69
D_{50}	4.76	1.34	268.83	133.94
D_{90}	18.19	6.34	813.33	400.98
$(D_{90}-D_{10})/D_{50}$	3.51	4.40	2.91	2.92

Table S2 The SEM EDS results of four solid wastes.

Sample	Element	Wt%	Wt% Sigma
	Na	8	0.15
	Al	3.69	0.07
	S	27.94	0.2
SW1	Ca	0	0.09
	Fe	54.1	0.3
	Zn	6.27	0.26
	Total:	100	
	Mg	0.25	0.02
	S	0.41	0.02
	Ca	0.66	0.03
SW2	Cr	49.27	0.23
	Fe	28.54	0.21
	Zn	20.87	0.12
	Total:	100	
	Mg	4.33	0.03
	P	0.94	0.02
	S	0.73	0.01
SW3	Ca	3.3	0.03
	Cr	69.61	0.17
	Fe	21.08	0.17
	Total:	100	
	Mg	14.63	0.11
	Al	4.7	0.07
	P	3.15	0.08
SW4	S	1.94	0.06
	Ca	30.44	0.2
	Fe	45.14	0.3
	Total:	100	

Table S3 The TEM EDS results of four solid wastes.

Sample	Element	Wt%	Wt% Sigma
SW1	Na	6.39	0.16
	Al	2.76	0.08
	S	33.09	0.18
	Ca	0.04	0.04
	Fe	57.26	0.2
	Zn	0.46	0.07
	Total：	100	
SW2	Mg	0.31	0.12
	S	0	0
	Ca	0.2	0.22
	Cr	1.21	0.35
	Fe	29.17	0.74
	Zn	69.11	0.79
	Total：	100	
SW3	Mg	15.54	0.34
	P	8.12	0.28
	S	4.75	0.23
	Ca	13.75	0.29
	Cr	17.47	0.34
	Fe	40.38	0.44
	Total：	100	
SW4	Mg	7.62	0.13
	Al	3.36	0.1
	P	1.09	0.08
	S	1.64	0.08
	Ca	29.49	0.19
	Fe	56.79	0.21
	Total：	100	

Table S4 Mass fraction of heavy metals and Cl, S in four solid wastes (ω mg·kg^{-1}).

Solid waste	Cd	Cu	Ni	Pb	Ce	As	Hg	Cl	S
SW1	75.8	1808	3.18	2977	1.91	2136	0.093	55.9	111000
SW2	78.8	301	67.1	3427	4.07	33.6	0.796	20448.4	3600
SW3	0.243	21.2	45.0	19.0	7.19	6.56	0.547	4676.9	10200
SW4	0.504	64.0	110	29.7	13.4	29.5	0.124	11863.6	6100

Table S5 The results of the BCR sequential extraction procedure (mg/kg).

Sample	Element	weak acid extractable state (mg/kg)	residual state (mg/kg)	Oxidizable state (mg/kg)	Reducible state (mg/kg)
SW1	Cd	19.500	56.900	0.567	0.662
	Cu	155.000	1550.000	100.000	31.800
	Ni	0.307	2.150	–	0.0531
	Pb	–	2169.000	432.000	243.000
	Ce	0.259	0.960	0.100	0.0851
SW2	Cd	44.400	16.100	1.780	21.900
	Cu	4.310	200.00	23.100	45.100
	Ni	0.297	52.800	1.410	2.320
	Pb	82.800	348.000	2453.000	565.000
	Ce	0.0238	0.563	1.760	0.400
SW3	Cd	0.134	0.0557	0.0849	0.0236
	Cu	0.393	4.580	8.760	0.438
	Ni	0.647	8.090	14.700	11.900
	Pb	–	6.660	4.000	0.320
	Ce	–	1.830	3.490	–
SW4	Cd	0.00554	0.0738	0.1250	0.162
	Cu	0.184	13.300	33.600	0.594
	Ni	2.020	22.700	45.300	24.300
	Pb	–	8.590	10.500	–
	Ce	–	2.180	7.850	0.00724

"–" Indicates the presence of the detected element.

Table S6 The Chinese National Standard for Surface Water Quality (GB 3838-2002) regarding selected heavy metals

**Heavy metals**	**Class I standard limit values**	**Class II standard limit values**	**Class III standard limit values**
Pb	≤ 0.01	≤ 0.01	≤ 0.05
Cd	≤ 0.001	≤ 0.005	≤ 0.005
As	≤ 0.05	≤ 0.05	≤ 0.05
Hg	≤ 0.00005	≤ 0.00005	≤ 0.0001
Cr^{6+}	≤ 0.01	≤ 0.05	≤ 0.05
Fe	≤ 0.1	≤ 0.1	≤ 0.3
Mn	≤ 0.1	≤ 0.1	≤ 0.1
Cu	≤ 0.01	≤ 1.0	≤ 1.0
Zn	≤ 0.05	≤ 1.0	≤ 1.0
Se	≤ 0.01	≤ 0.01	≤ 0.01
F^-	≤ 1.0	≤ 1.0	≤ 1.0

Table S7 the Chinese National Standard for Groundwater Quality (GB/T14848-2017) regarding specific heavy metals

Heavy metals	Class I standard limit values	Class II standard limit values	Class III standard limit values
**Pb**	≤ 0.005	≤ 0.01	≤ 0.01
**Cd**	≤ 0.0001	≤ 0.001	≤ 0.005
**As**	≤ 0.001	≤ 0.001	≤ 0.01
**Hg**	≤ 0.0001	≤ 0.0001	≤ 0.001
**Cr^{6+}**	≤ 0.005	≤ 0.01	≤ 0.05
**Fe**	≤ 0.1	≤ 0.2	≤ 0.3
**Mn**	≤ 0.05	≤ 0.05	≤ 0.10
**Cu**	≤ 0.01	≤ 0.05	≤ 1.00
**Zn**	≤ 0.05	≤ 0.5	≤ 1.00
**Se**	≤ 0.001	≤ 0.01	≤ 0.01
**F^-**	≤ 1.0	≤ 1.0	≤ 1.0

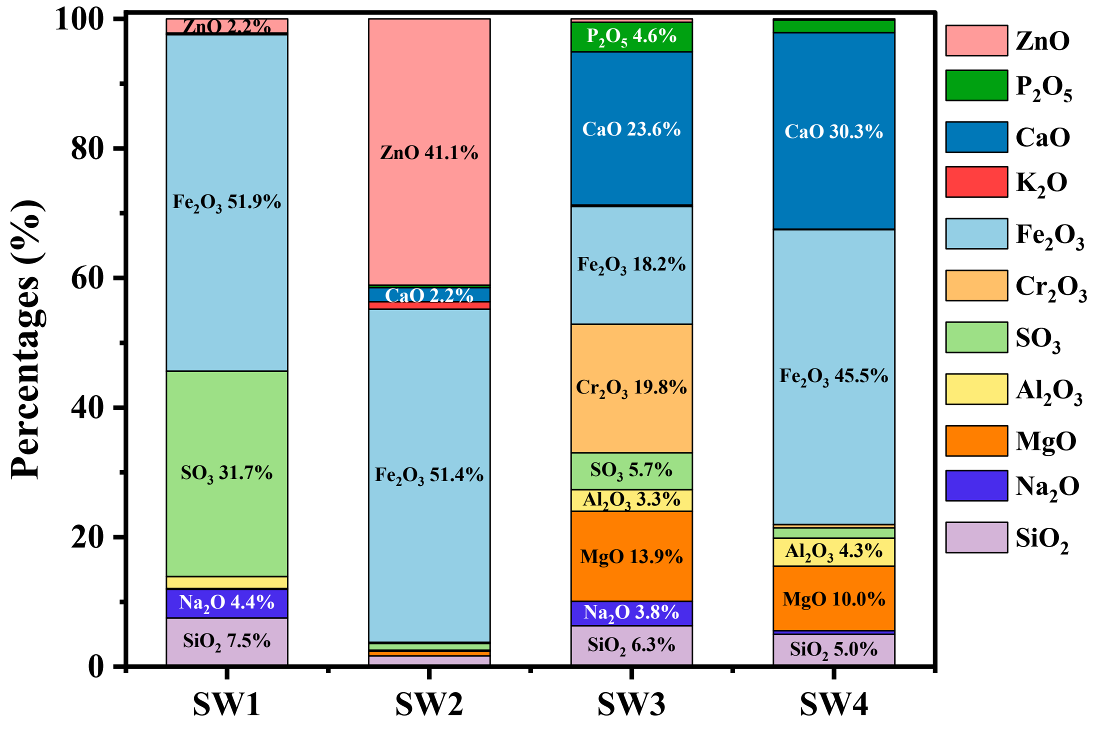

Figure S1. The chemical composition of four solid wastes.

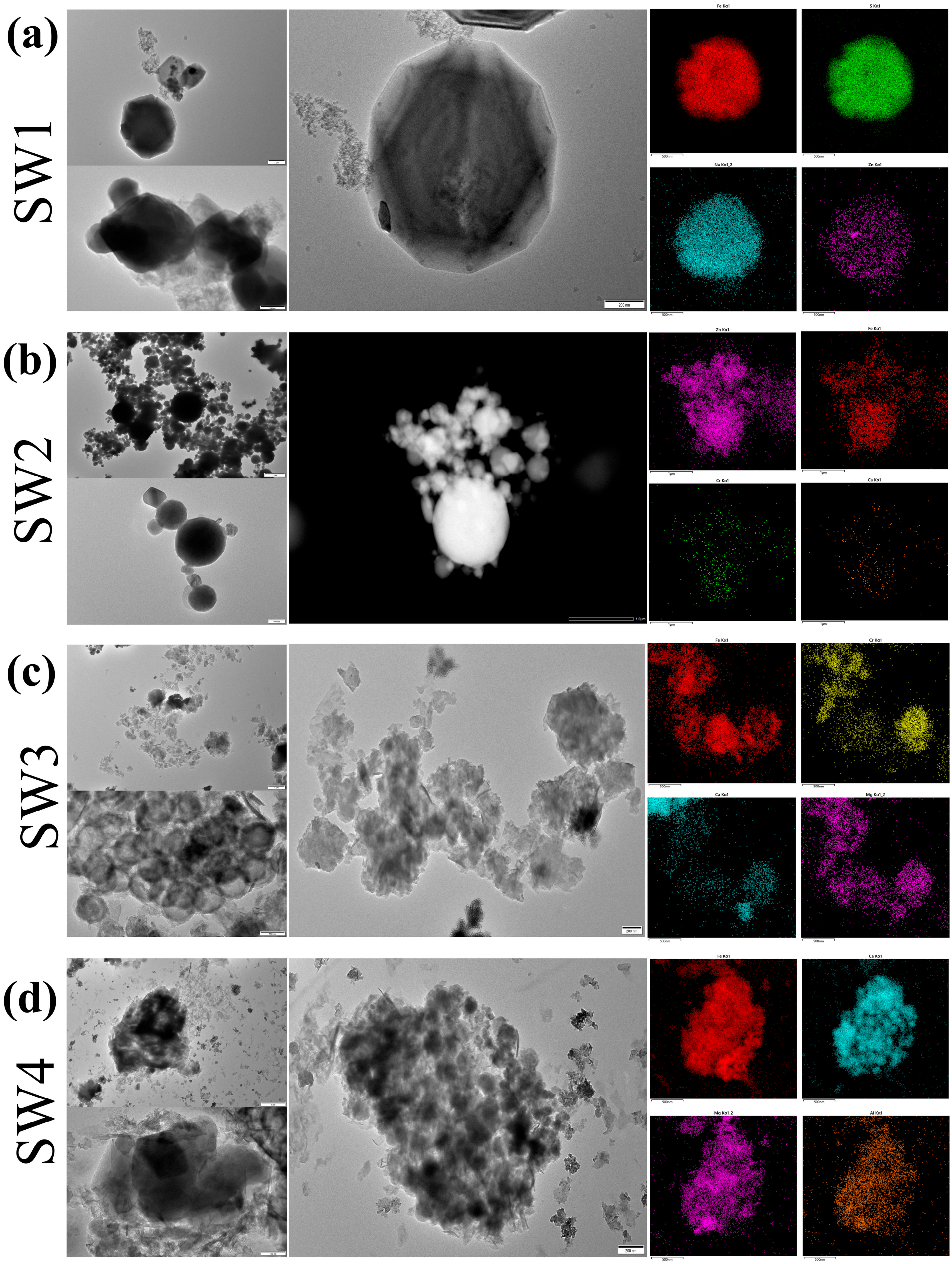

Figure S2. The TEM of four solid wastes.

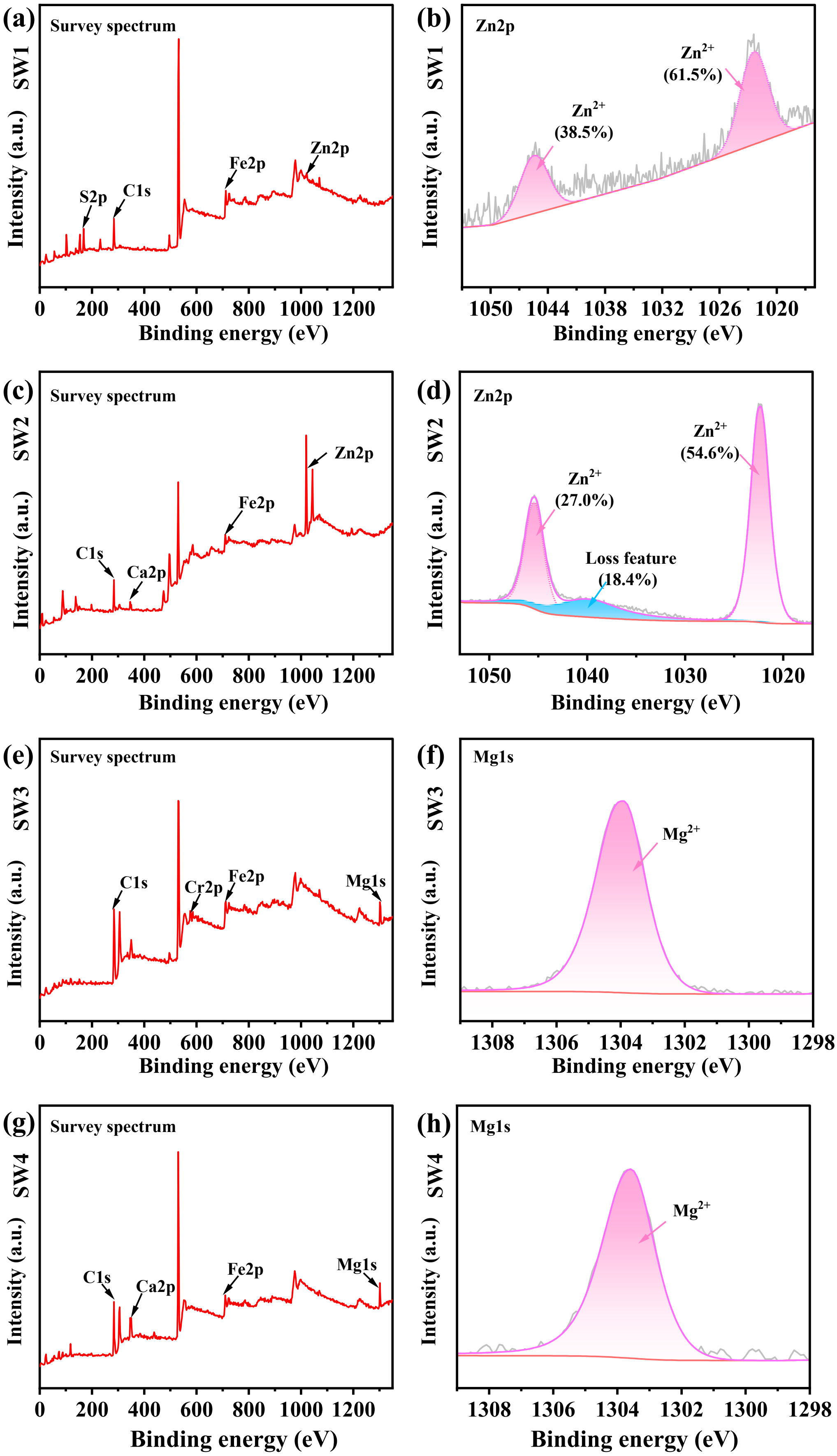

Figure S3. The XPS spectrum of four solid wastes.

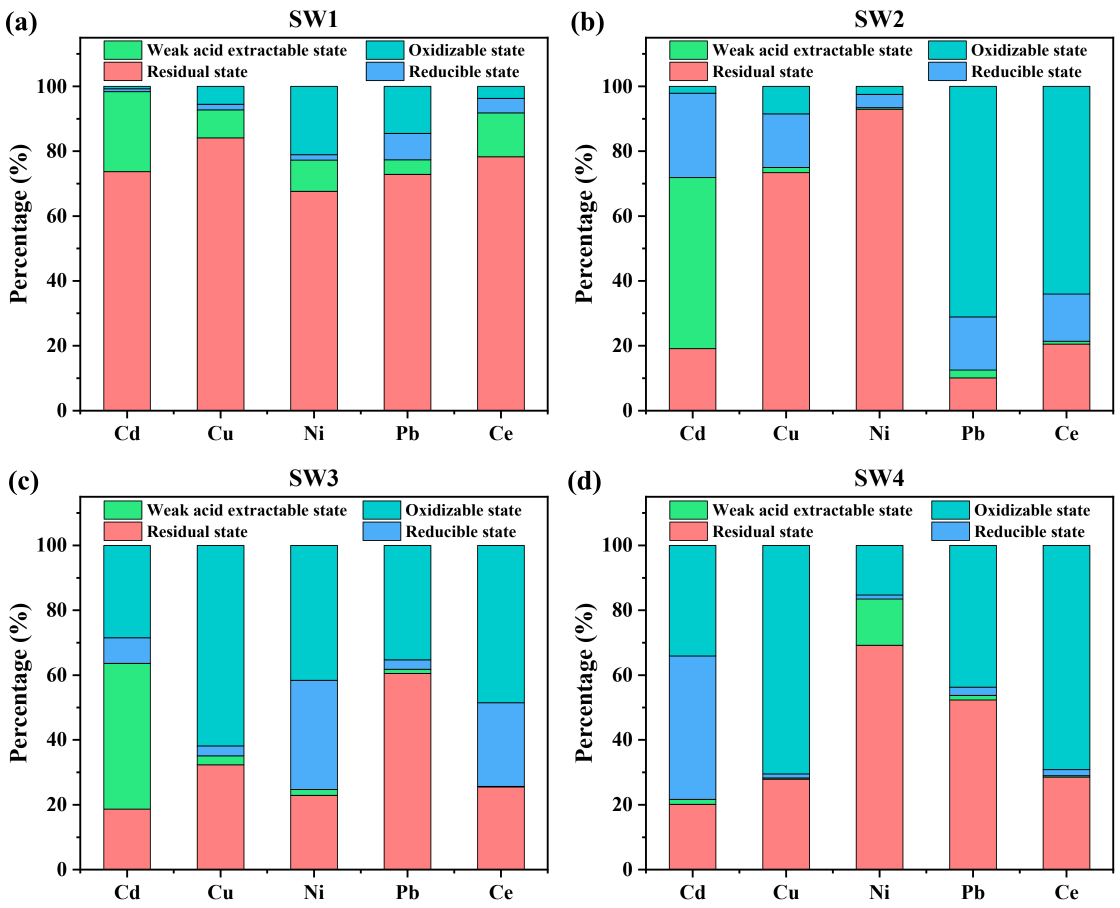

Figure S4. The occurrence state of metal elements in four solid wastes.
