# Supplementary figures and images for: Heavy Metals Environmental Fate in Metallurgical Solid Wastes: Occurrence, Leaching, and Ecological Risk Assessment
Source: J Xenobiot. 2025 Dec 15;15(6):211. doi: 10.3390/jox15060211 (PMC12733436; doi:10.3390/jox15060211)

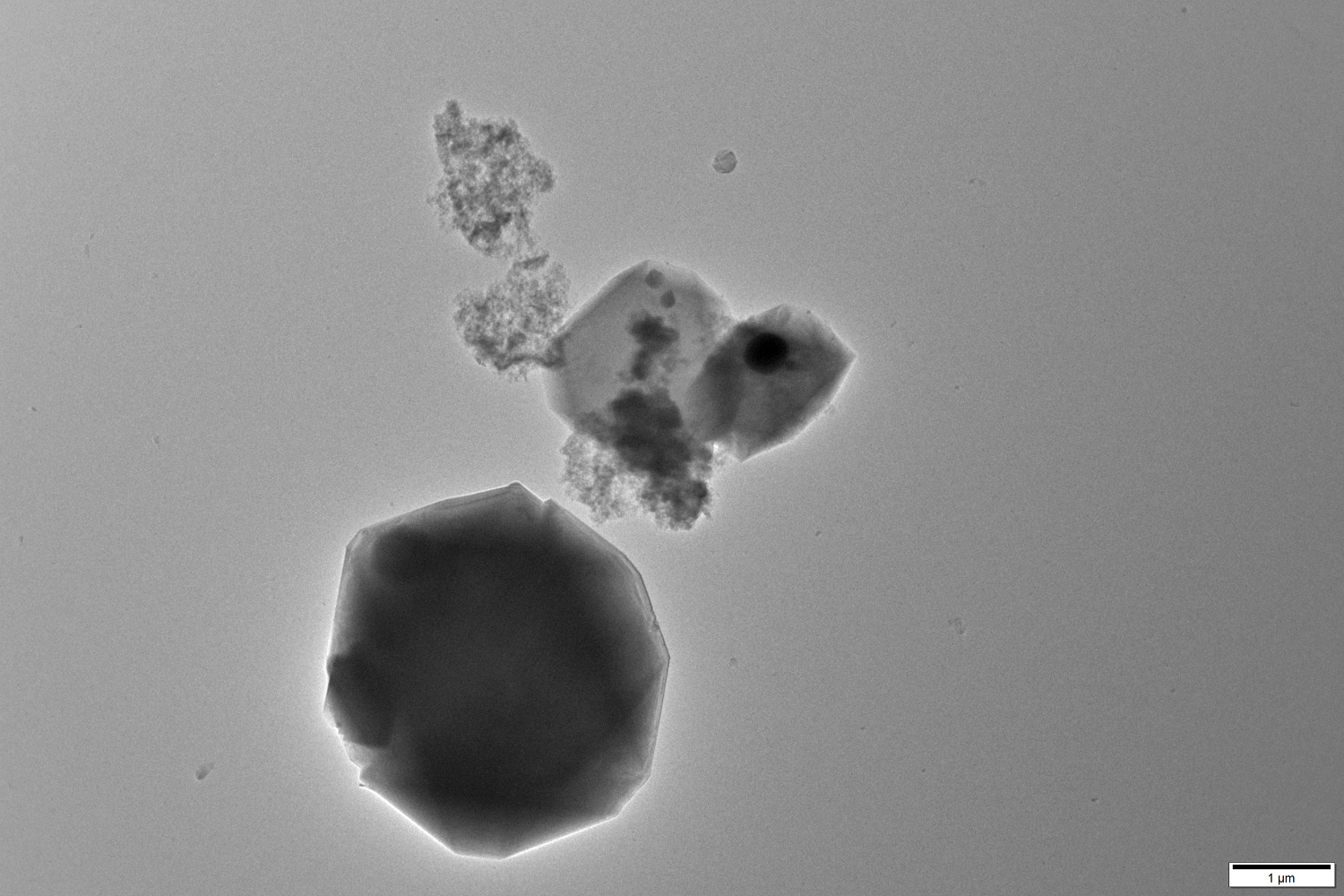

Supplement: Supplementary file 1 [file jox-15-00211-s001.zip › FileS1-Original images of Figures 3 and S2/FigureS2/FigureS2a SW1/1-1.tif]

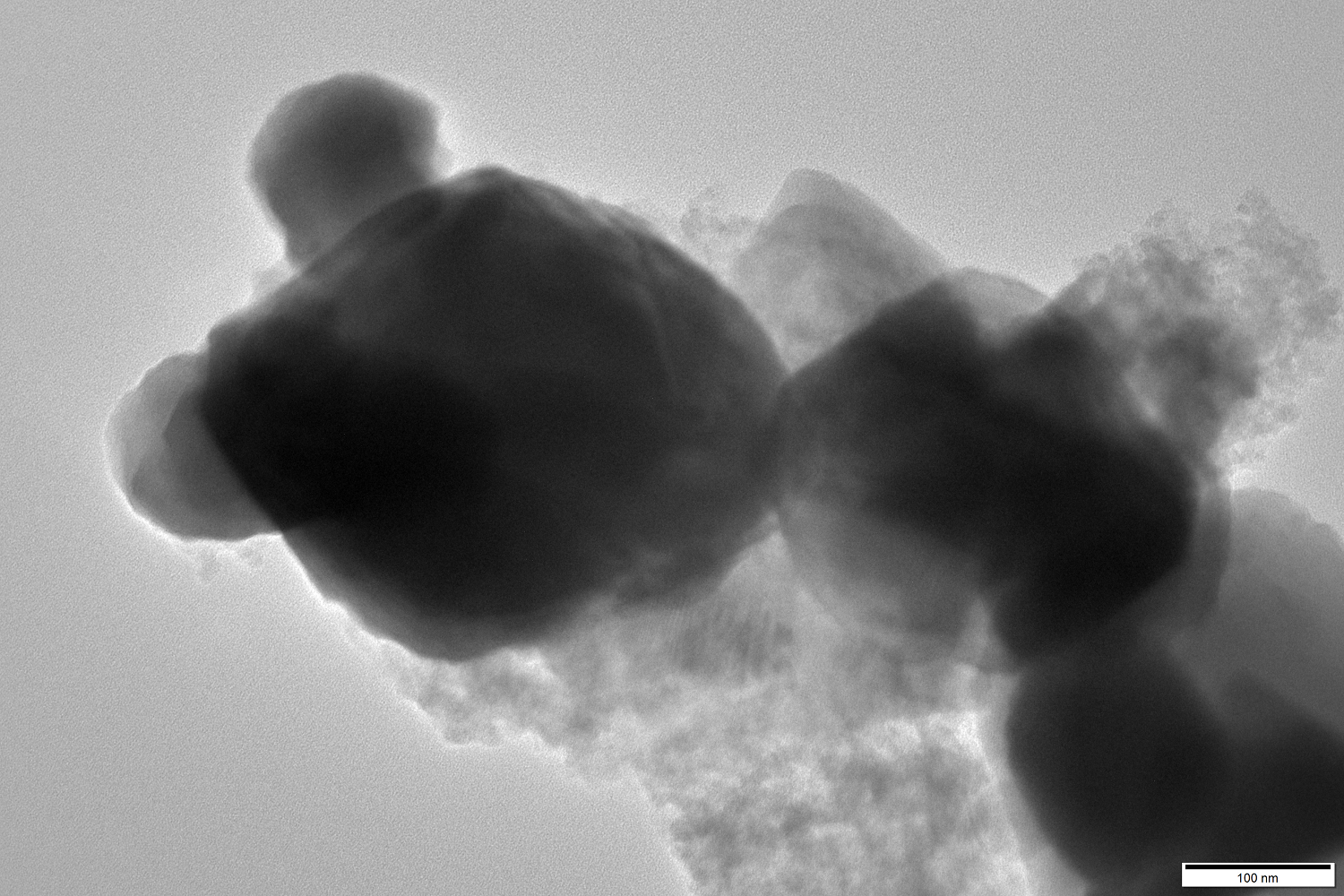

Supplement: Supplementary file 1 [file jox-15-00211-s001.zip › FileS1-Original images of Figures 3 and S2/FigureS2/FigureS2a SW1/1-2.tif]

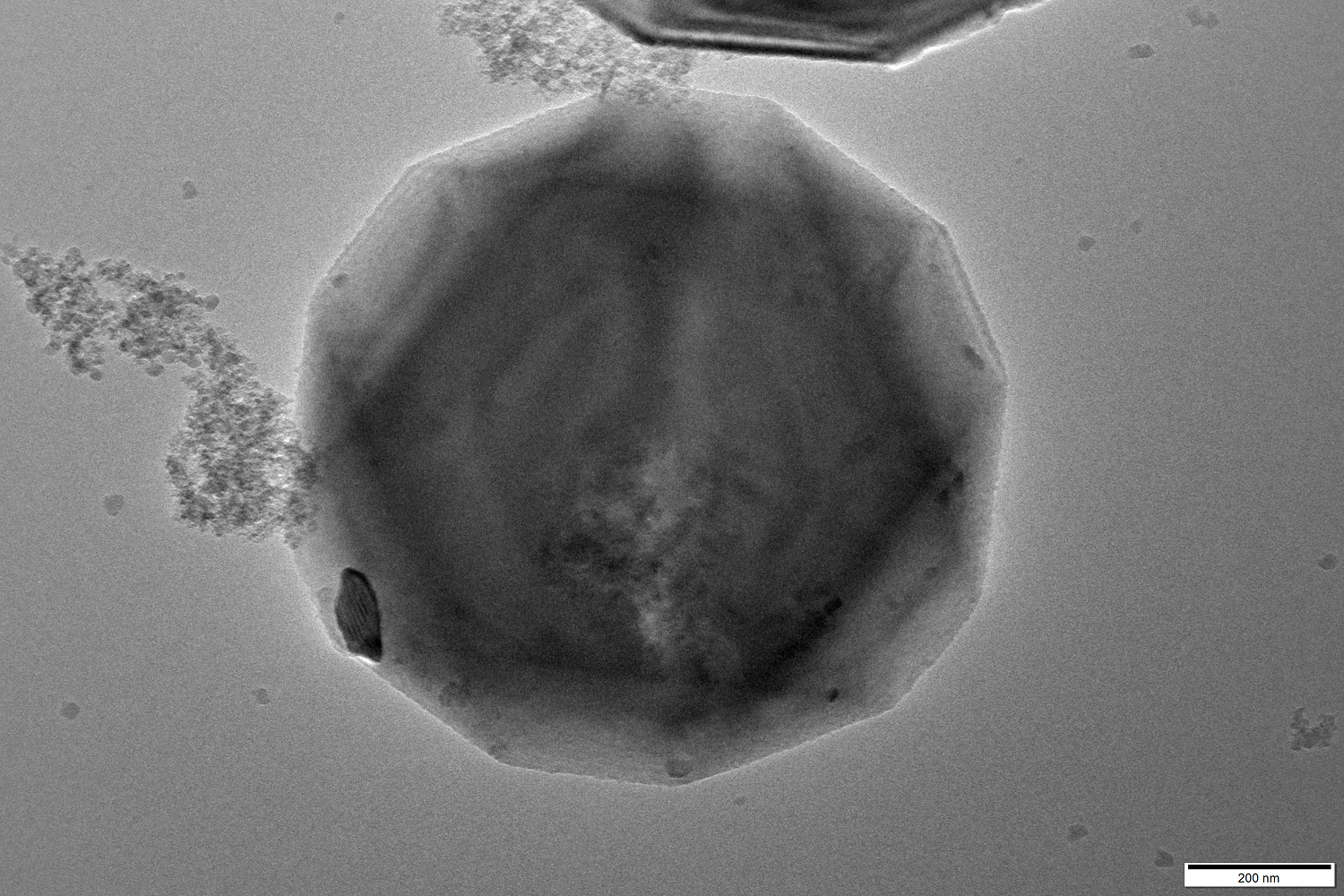

Supplement: Supplementary file 1 [file jox-15-00211-s001.zip › FileS1-Original images of Figures 3 and S2/FigureS2/FigureS2a SW1/1-3 mapping.tif]

EDS 分层图像 1

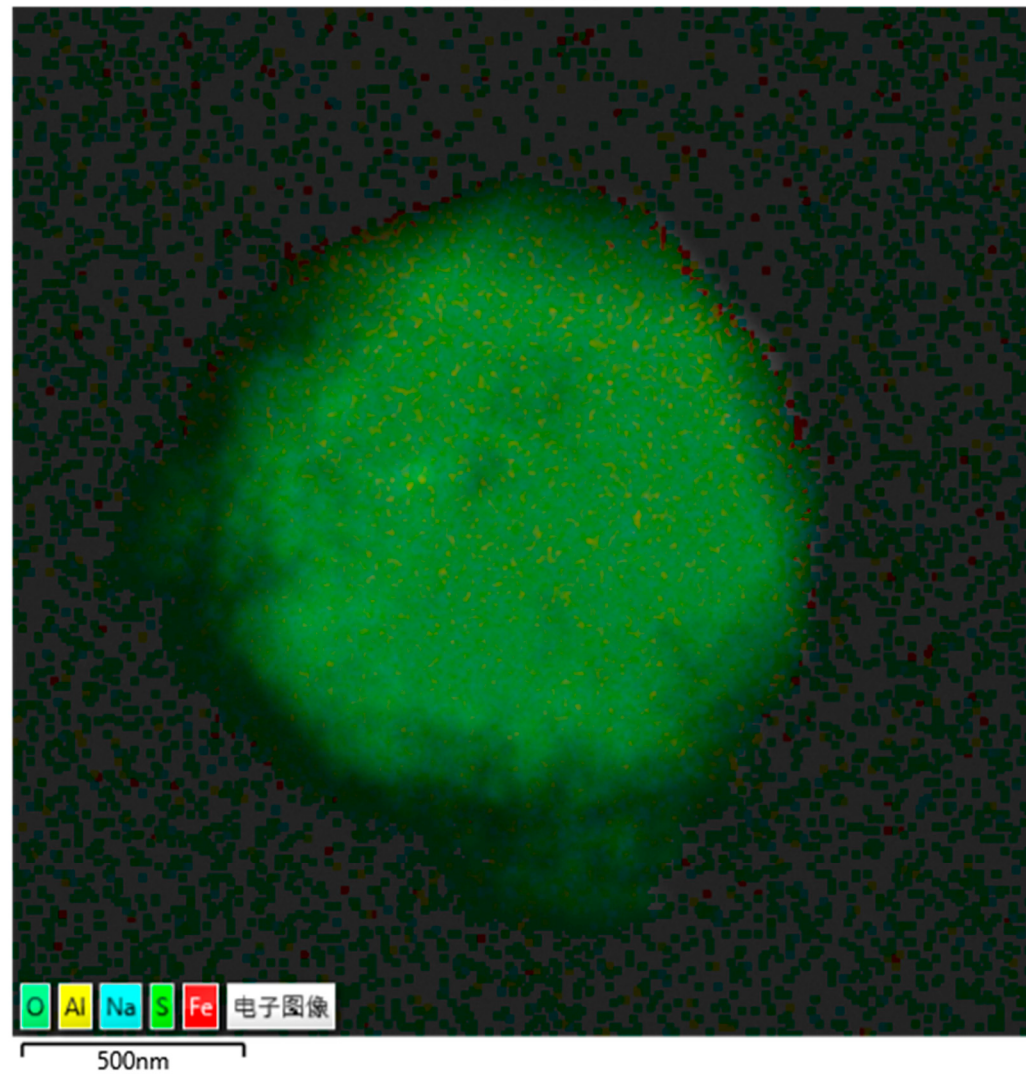

电子图像 1

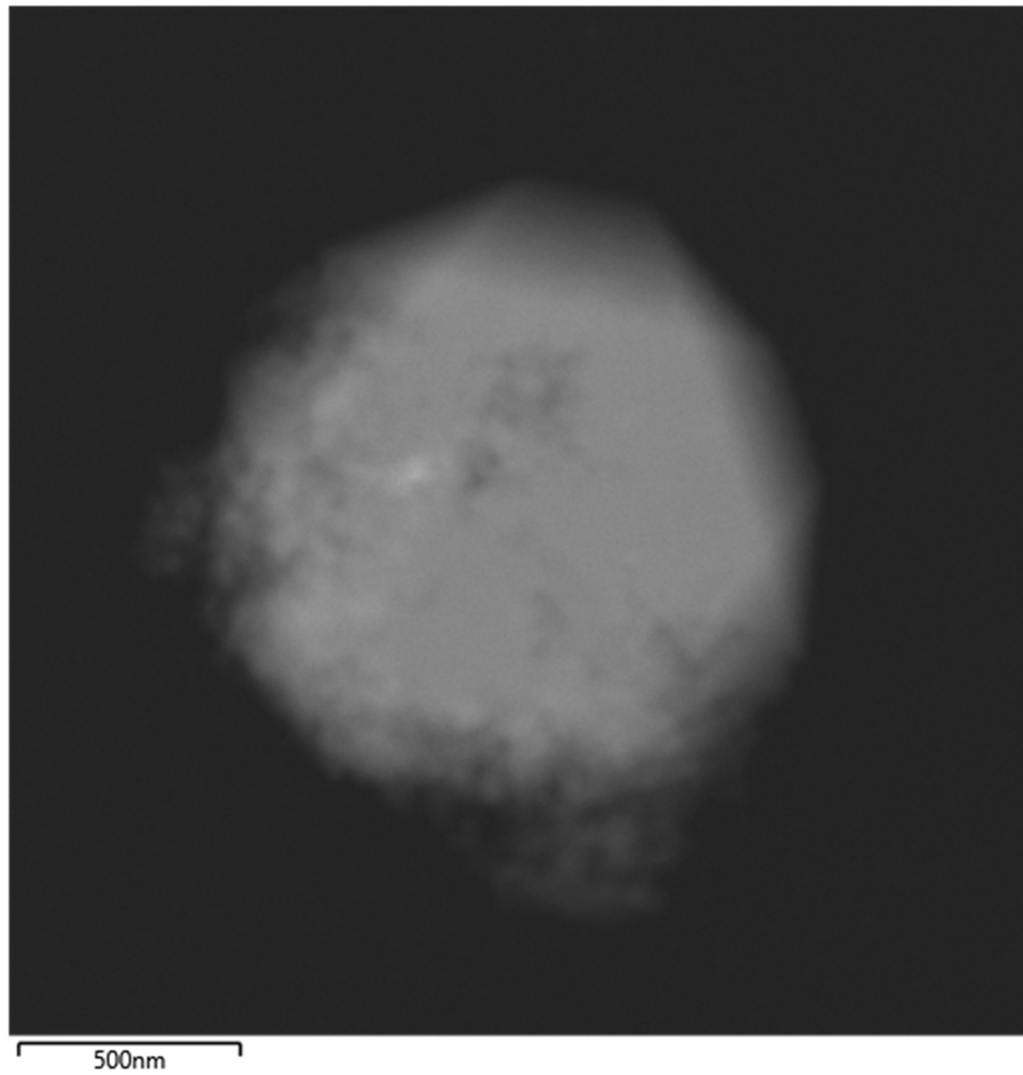

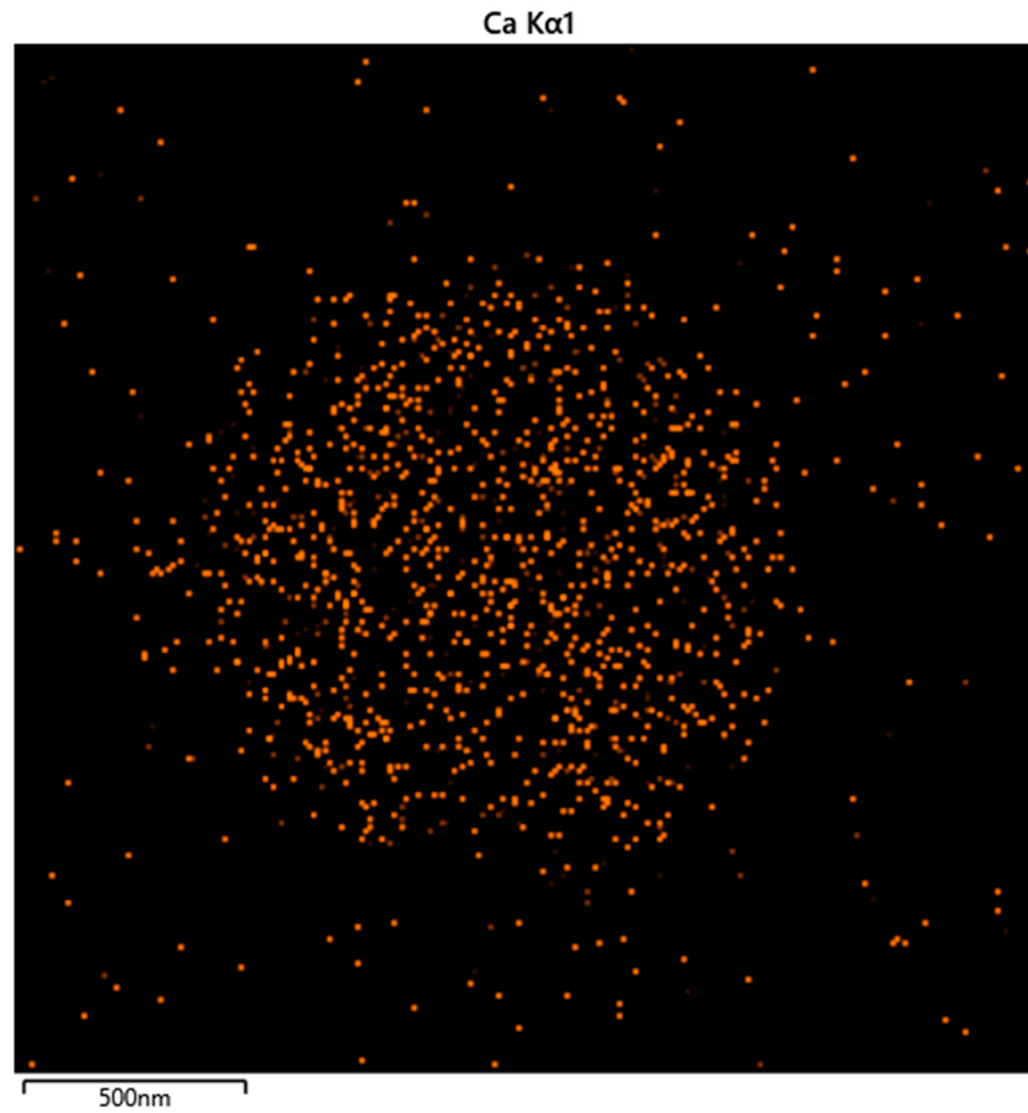

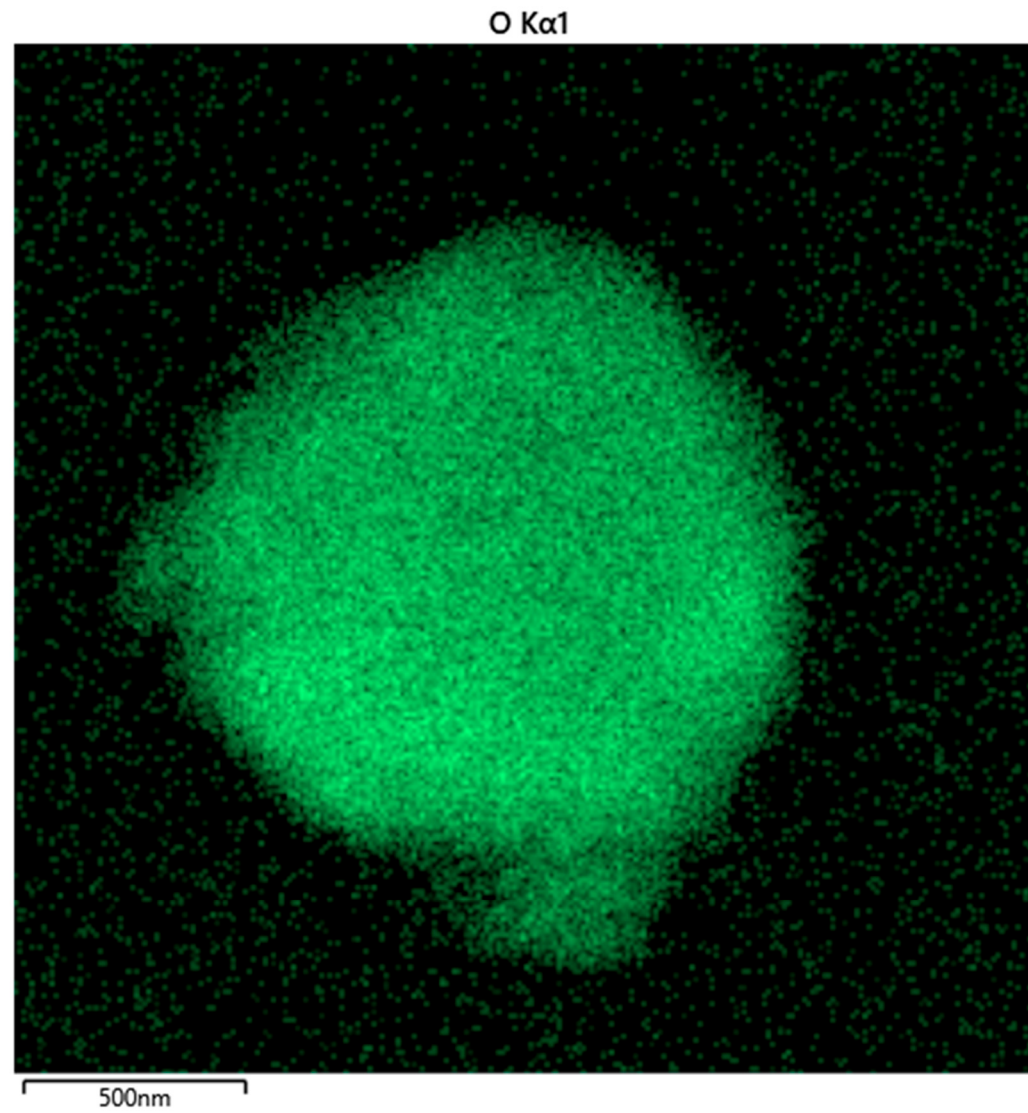

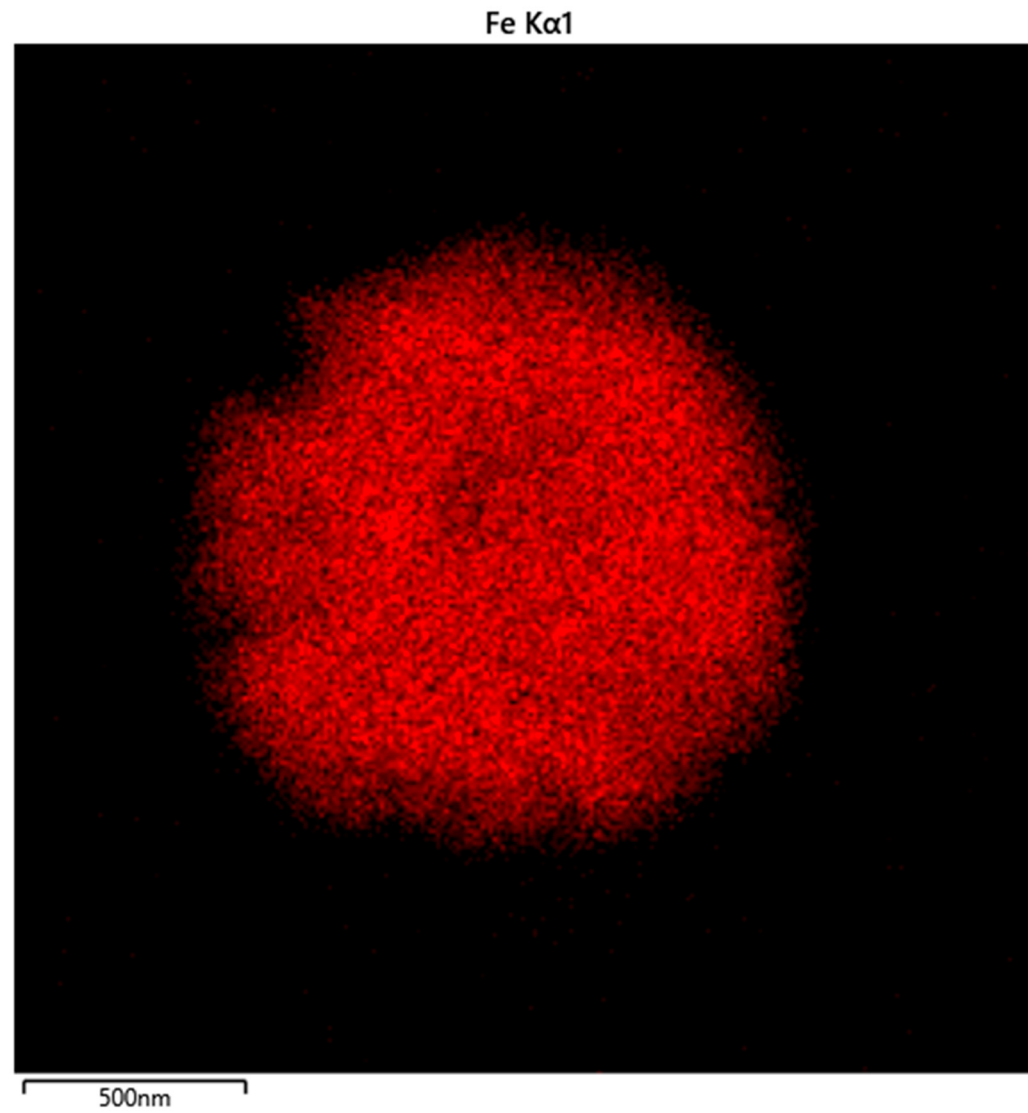

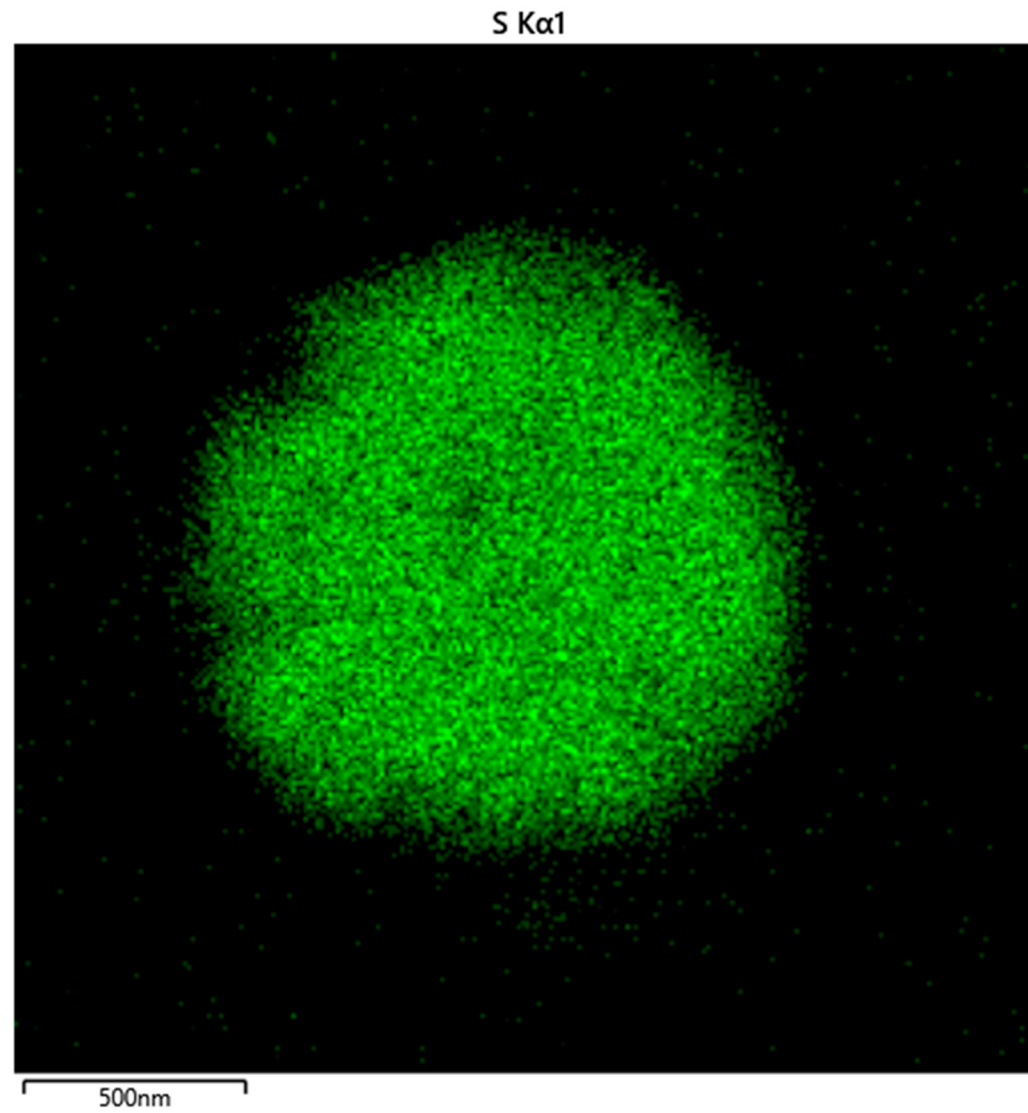

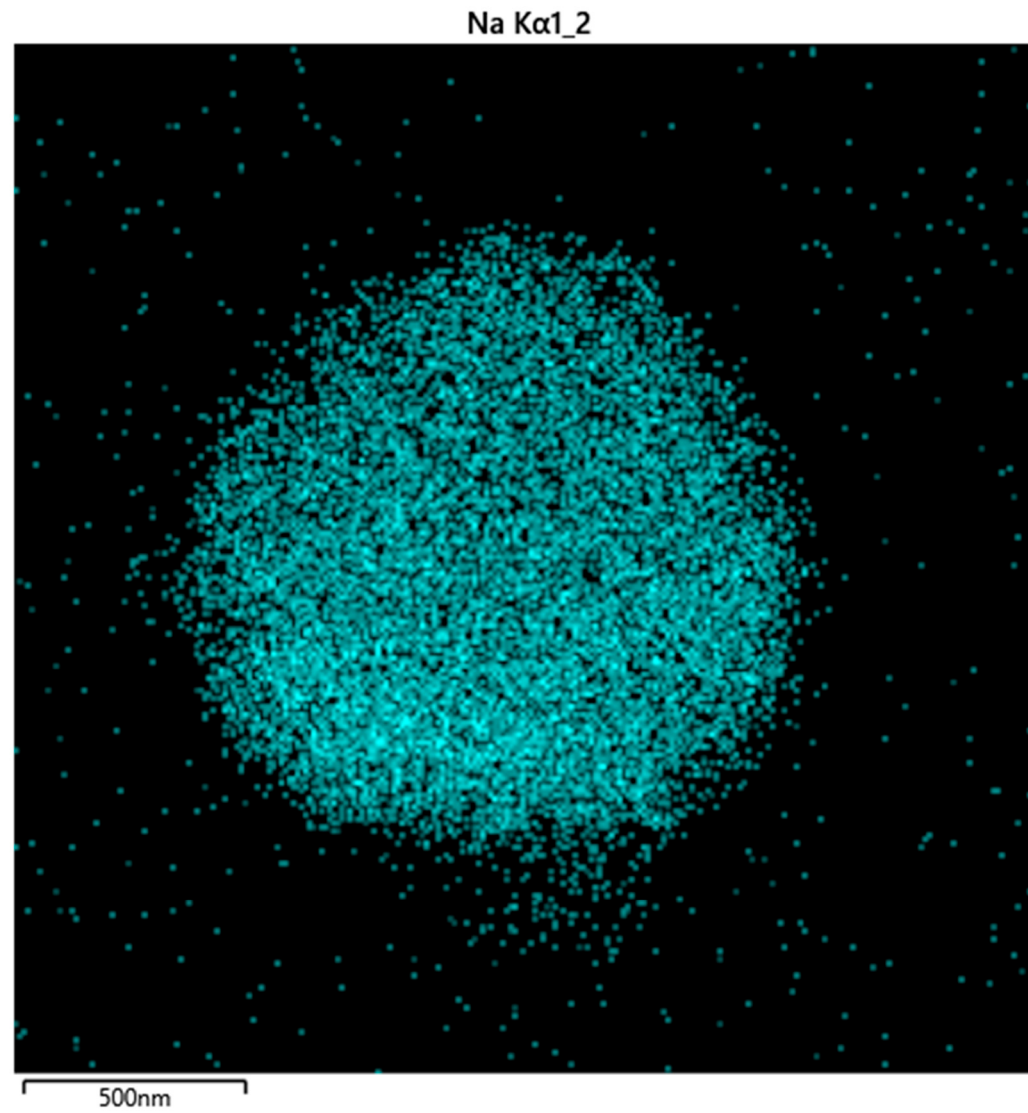

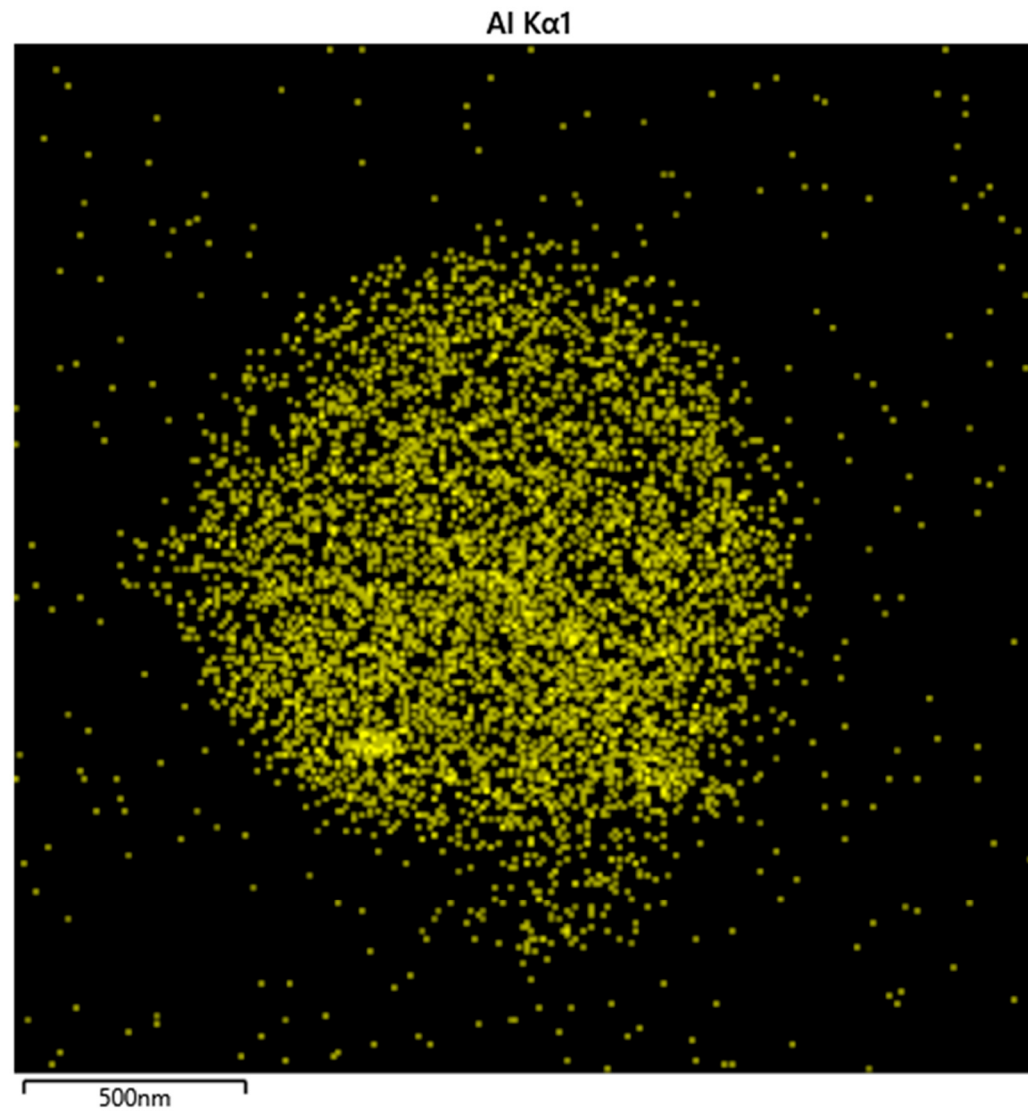

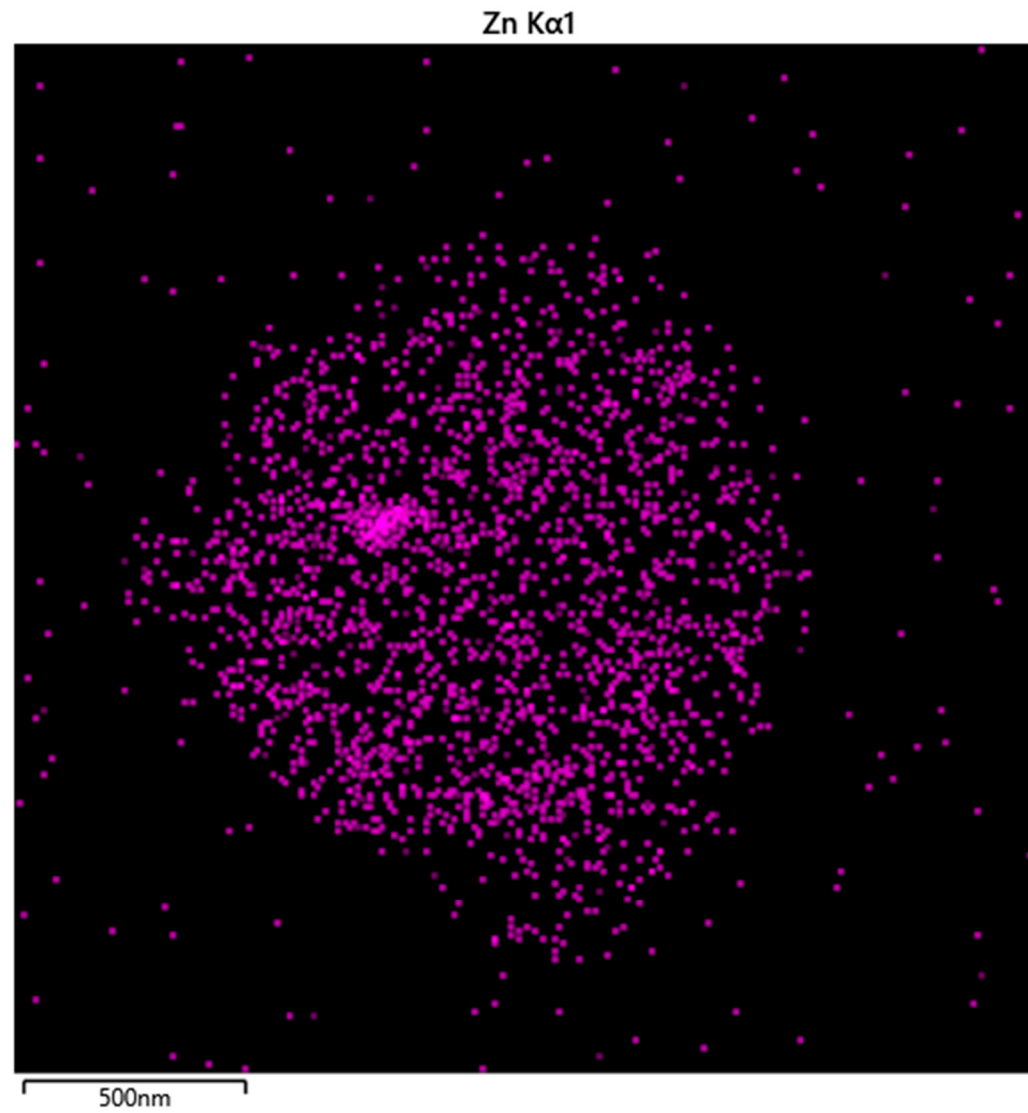

Supplement: Supplementary file 1 [file jox-15-00211-s001.zip › FileS1-Original images of Figures 3 and S2/FigureS2/FigureS2a SW1/1-mapping.pdf]

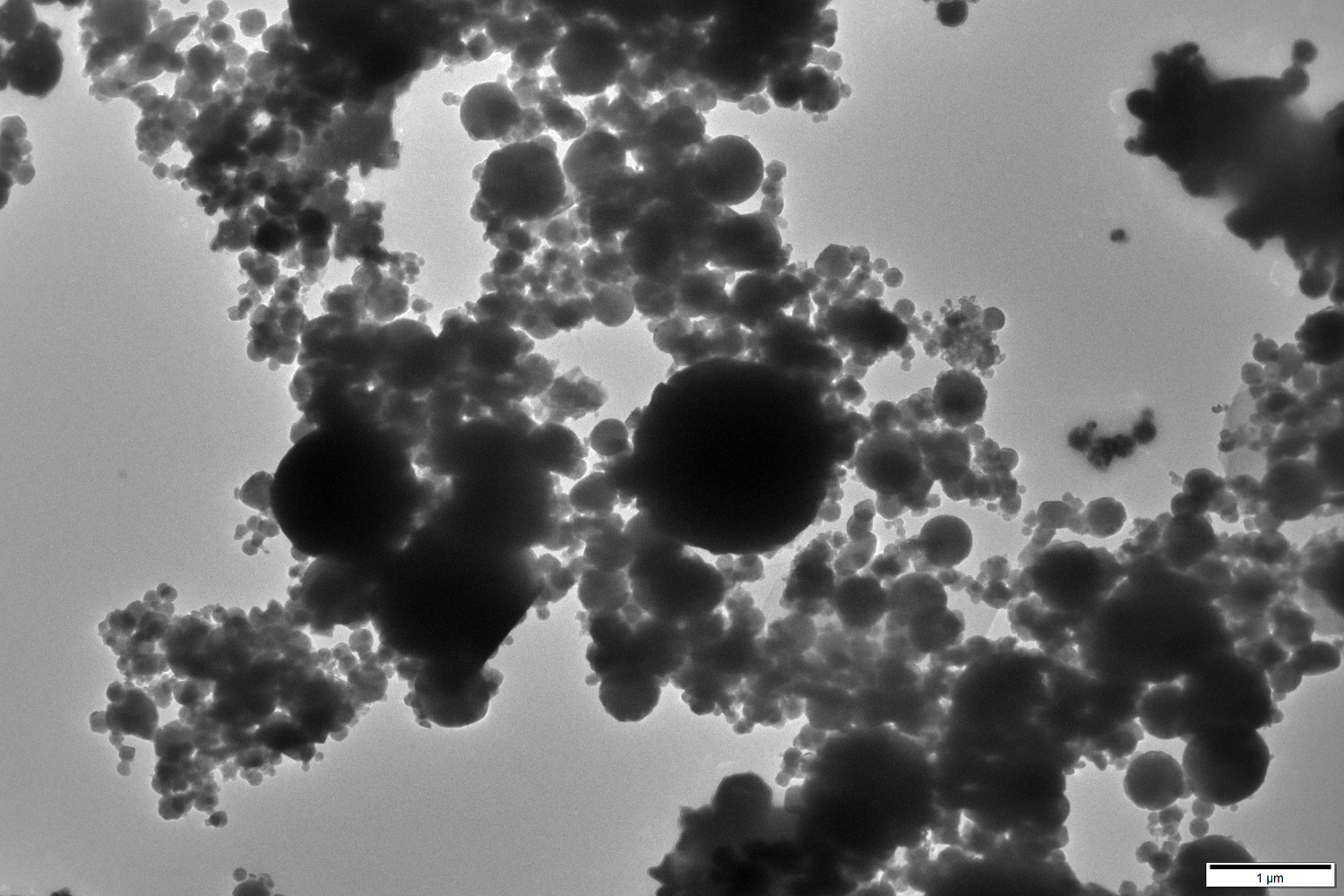

Supplement: Supplementary file 1 [file jox-15-00211-s001.zip › FileS1-Original images of Figures 3 and S2/FigureS2/FigureS2b SW2/2-1.tif]

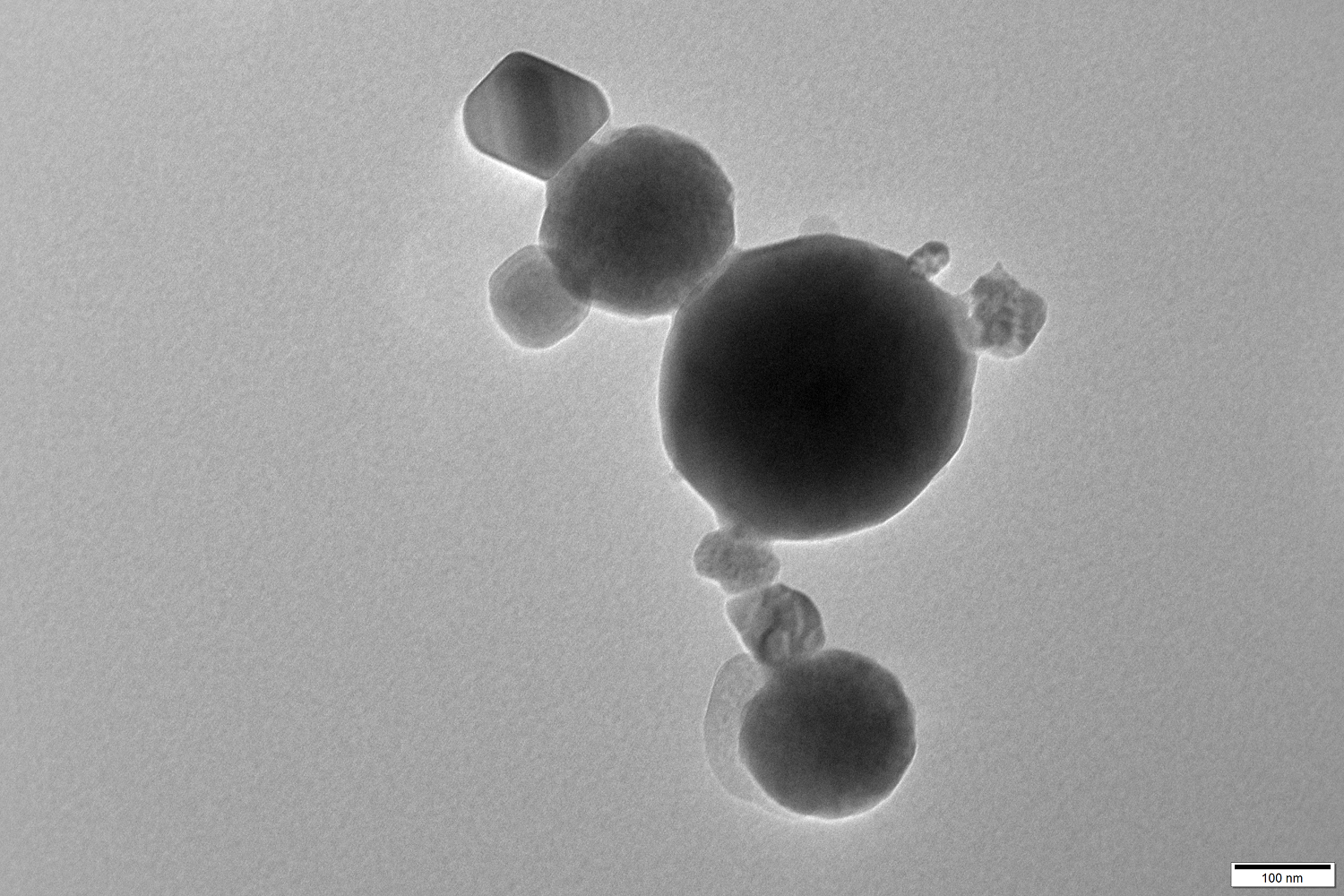

Supplement: Supplementary file 1 [file jox-15-00211-s001.zip › FileS1-Original images of Figures 3 and S2/FigureS2/FigureS2b SW2/2-2.tif]

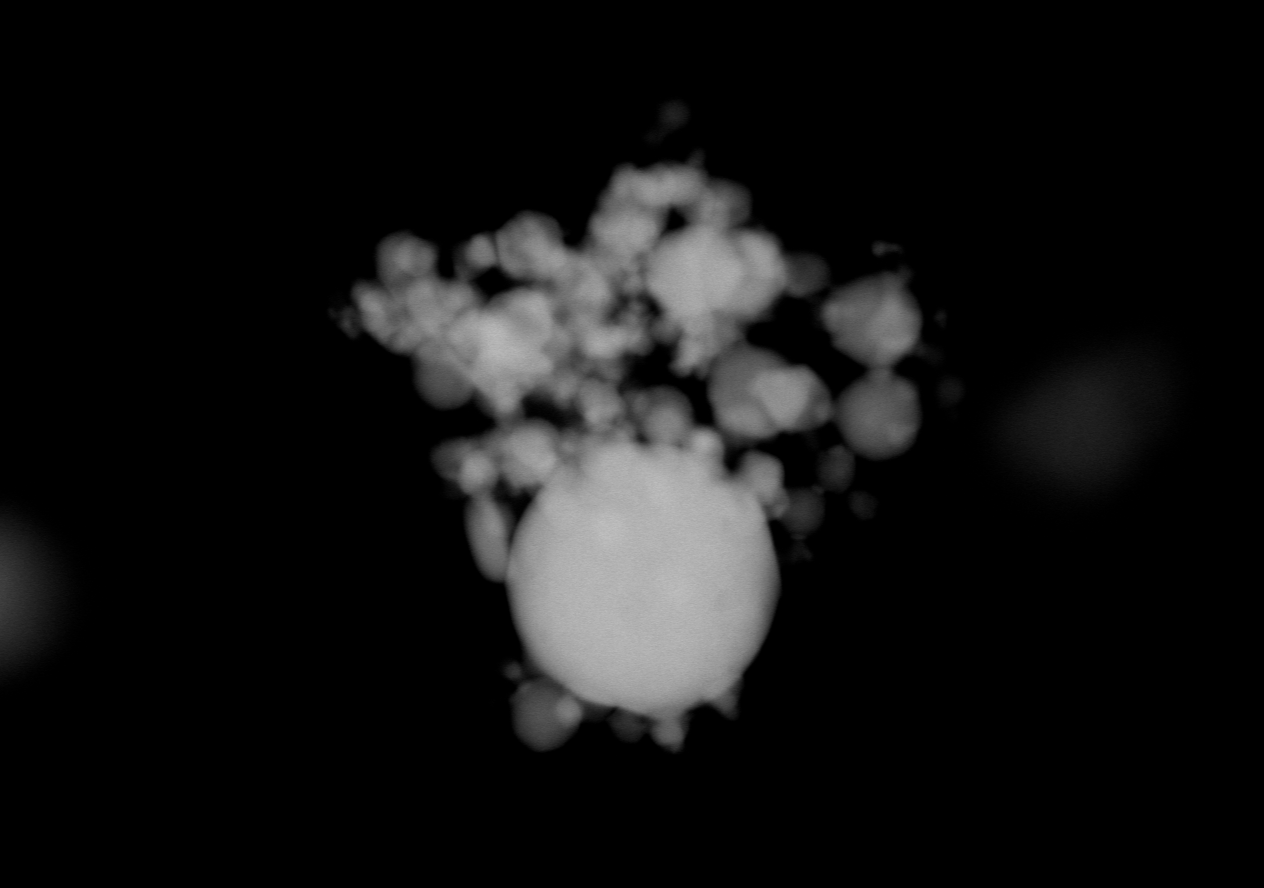

Supplement: Supplementary file 1 [file jox-15-00211-s001.zip › FileS1-Original images of Figures 3 and S2/FigureS2/FigureS2b SW2/2-3 mapping.tif]

EDS 分层图像 2

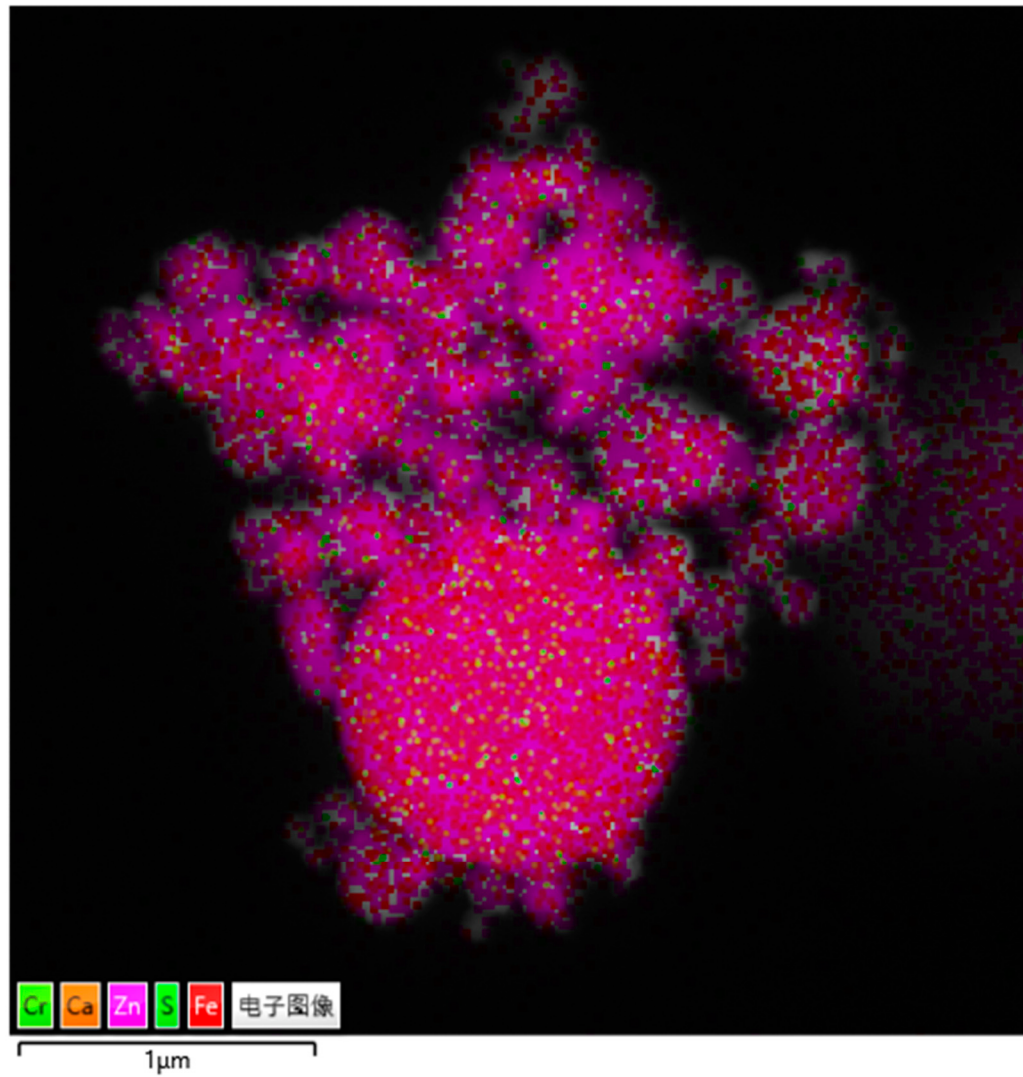

电子图像 2

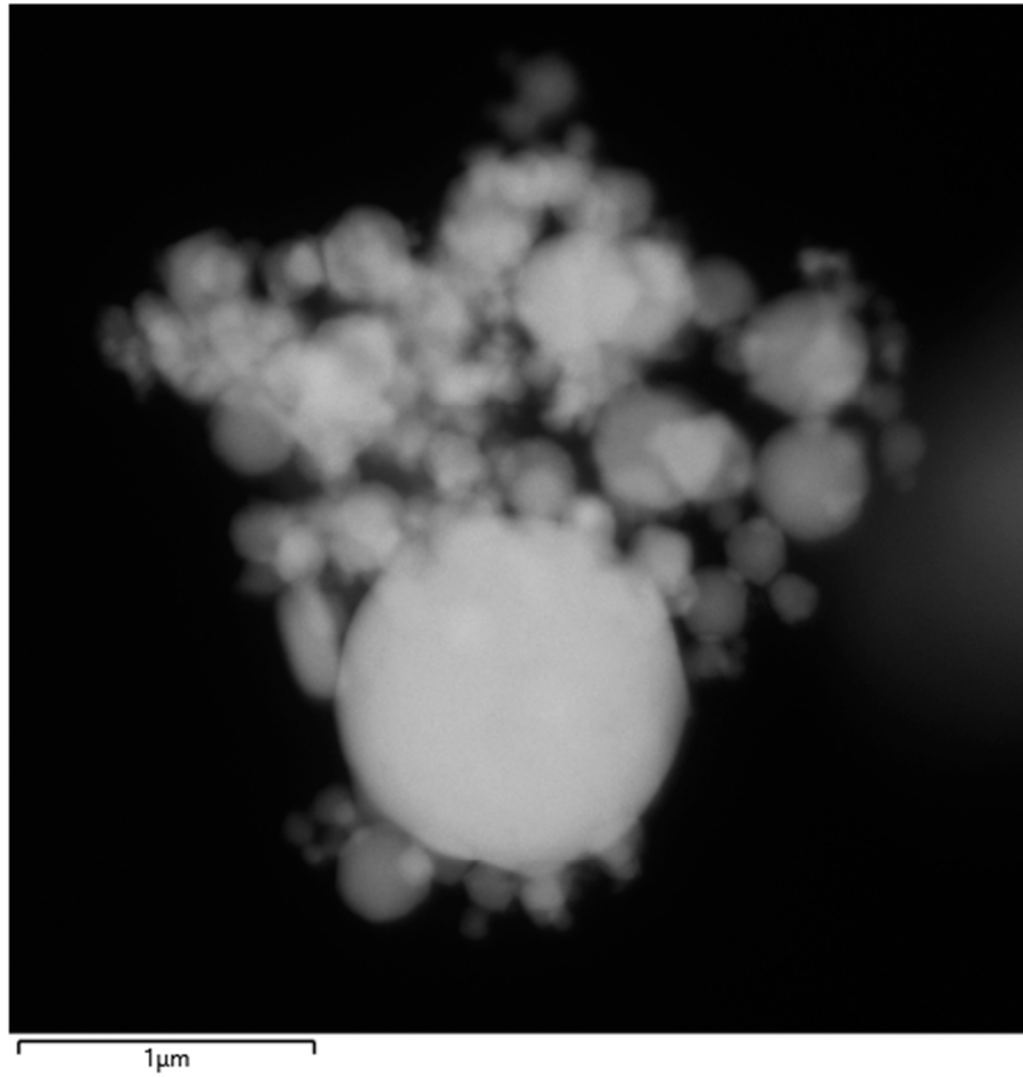

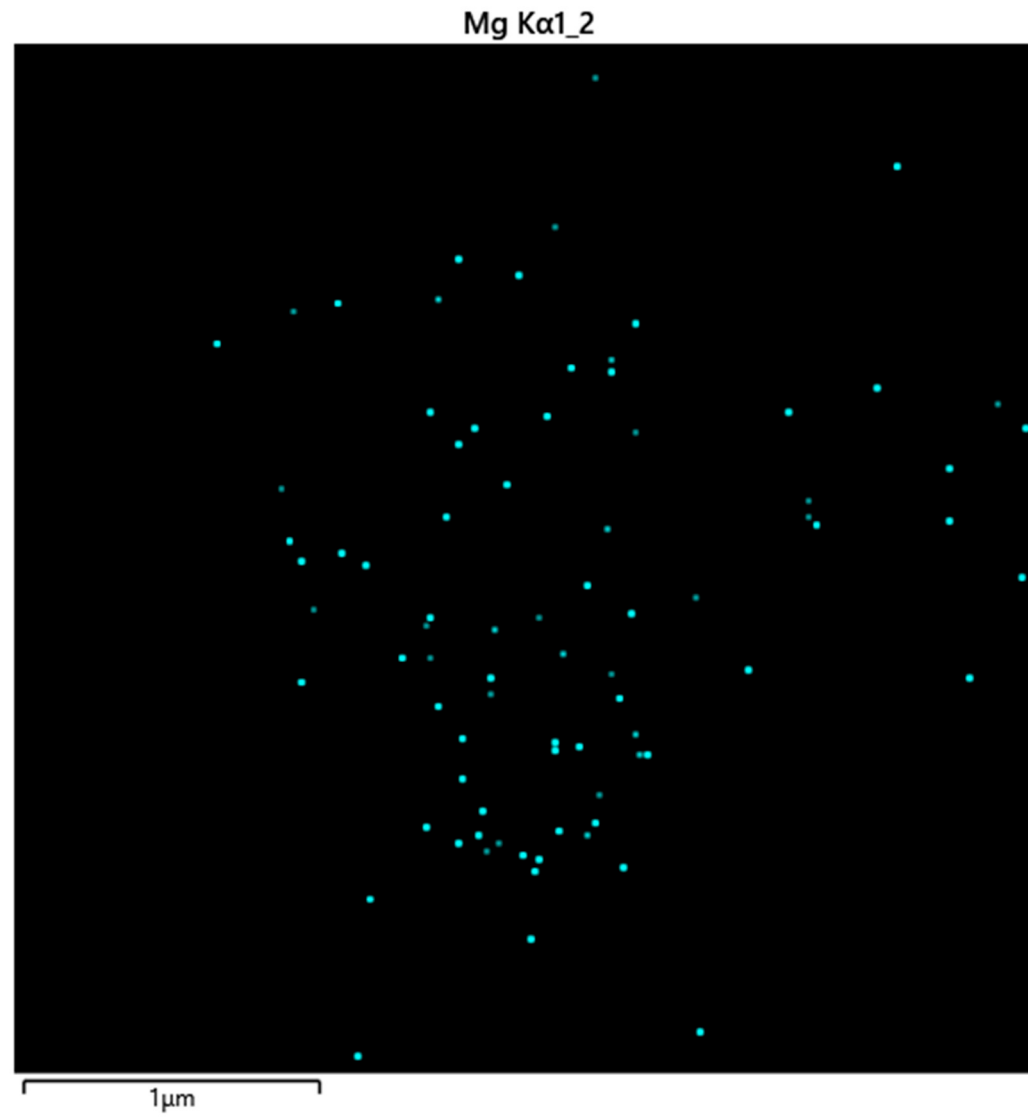

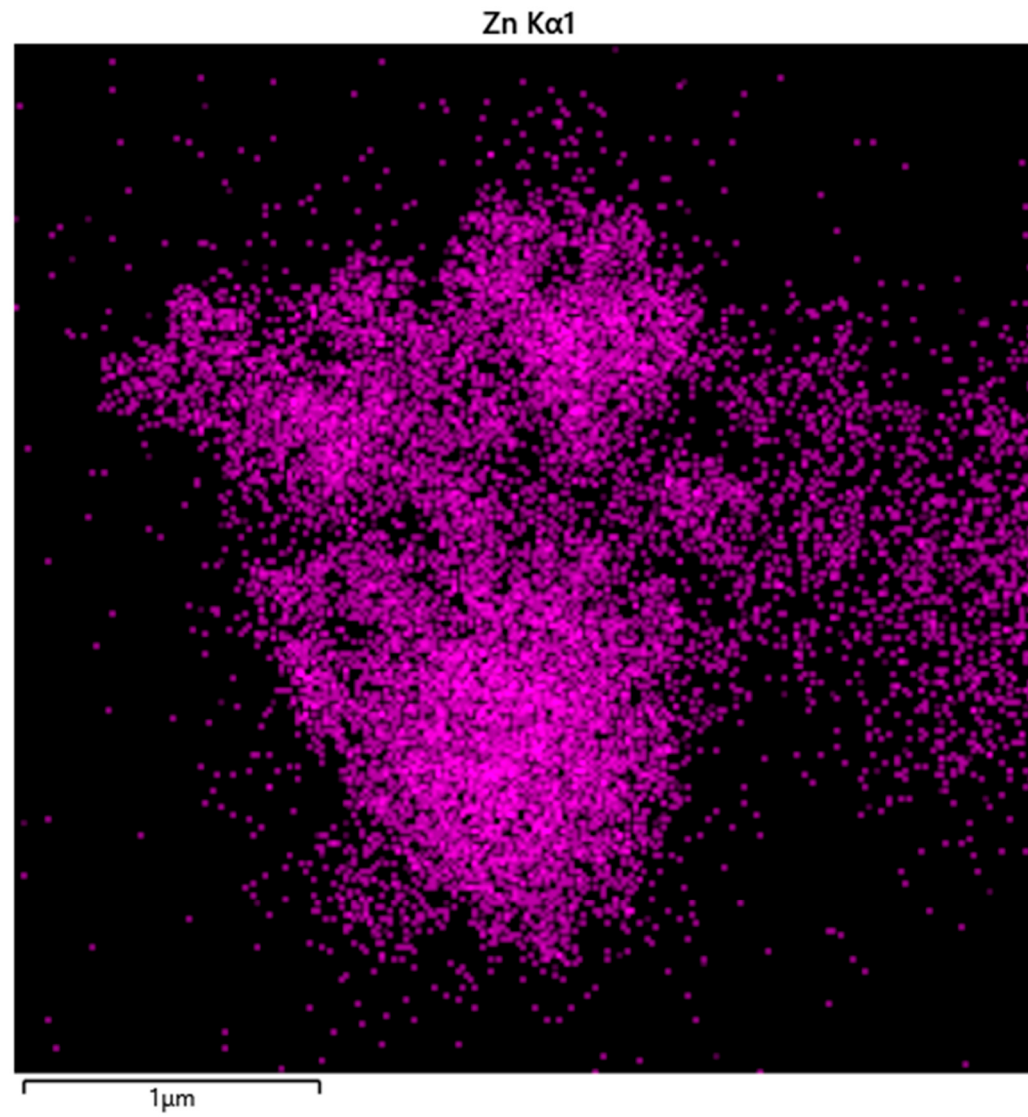

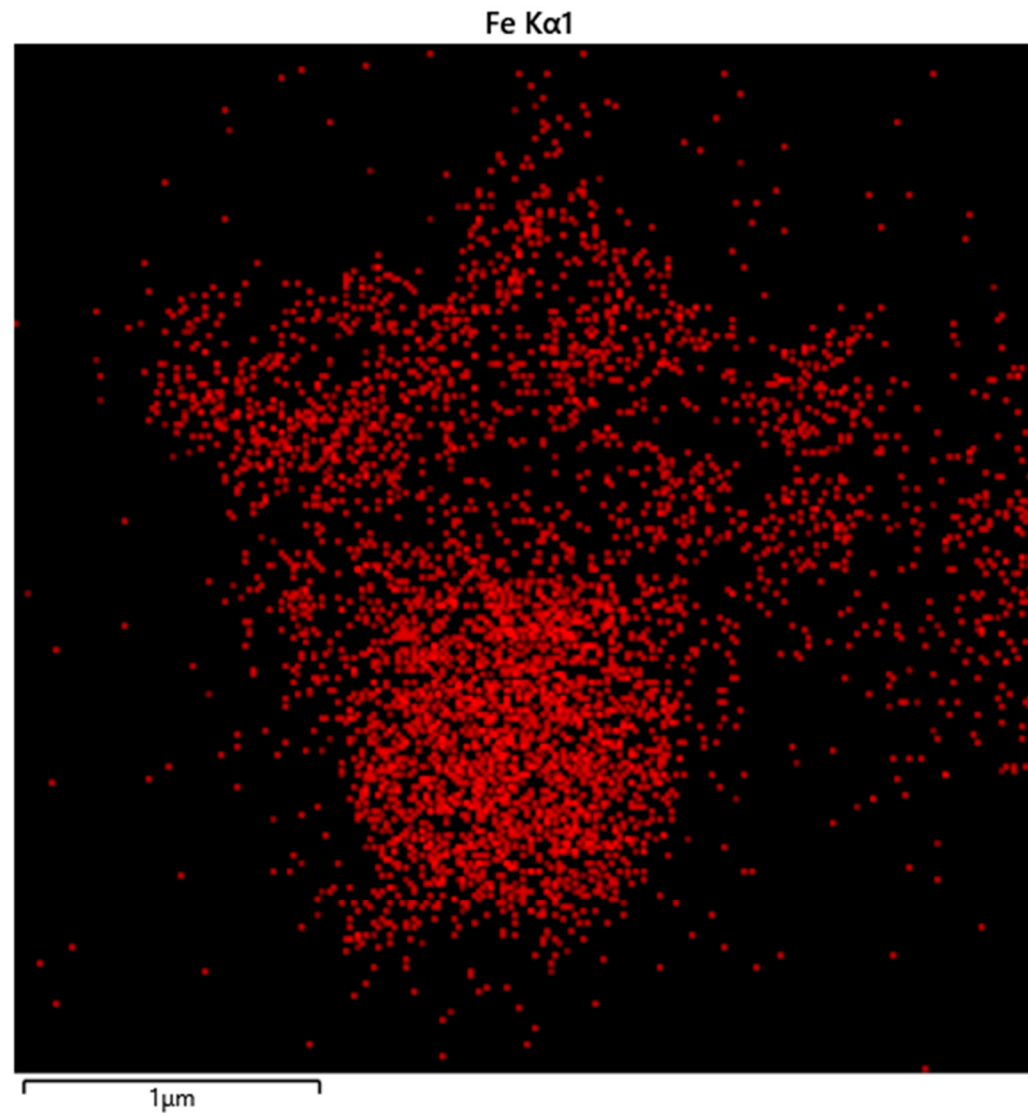

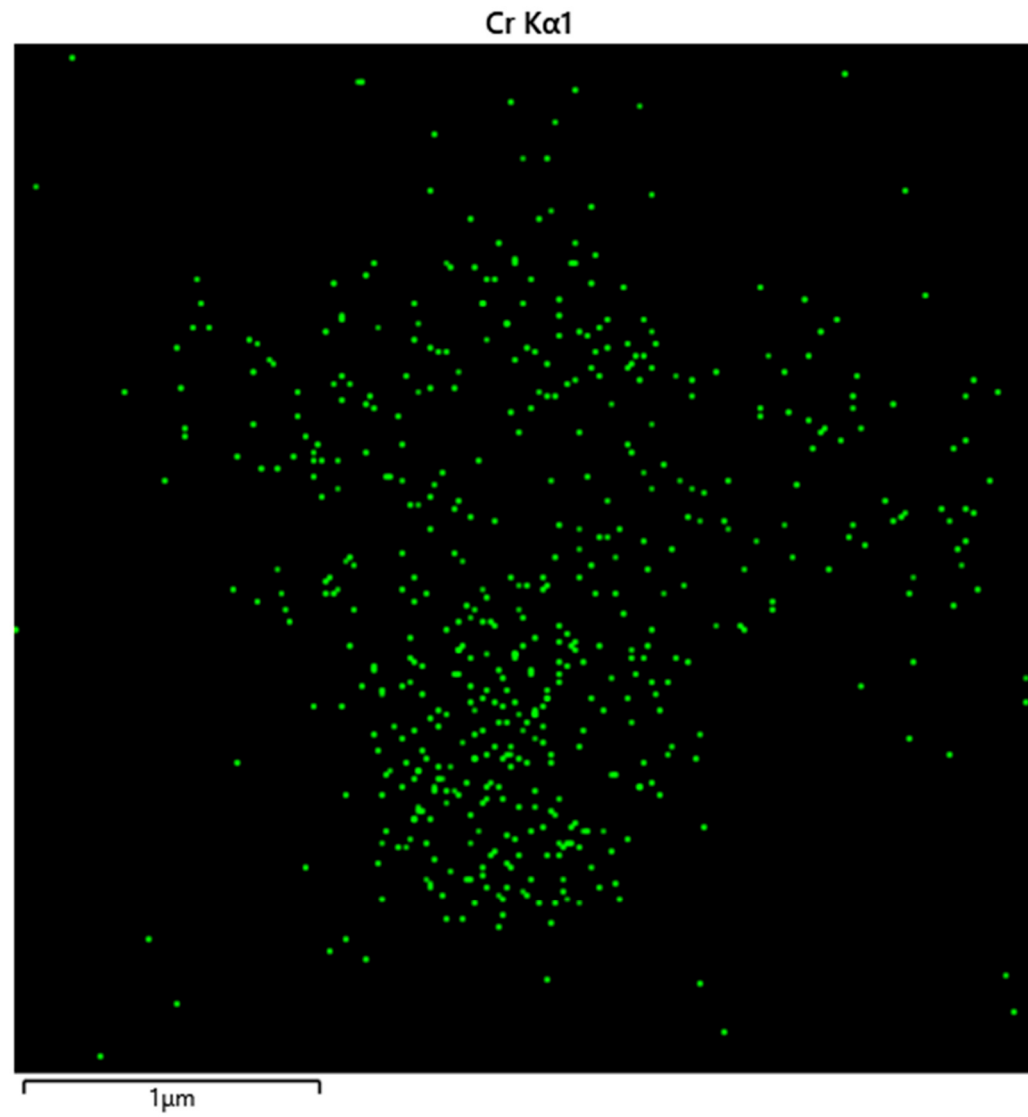

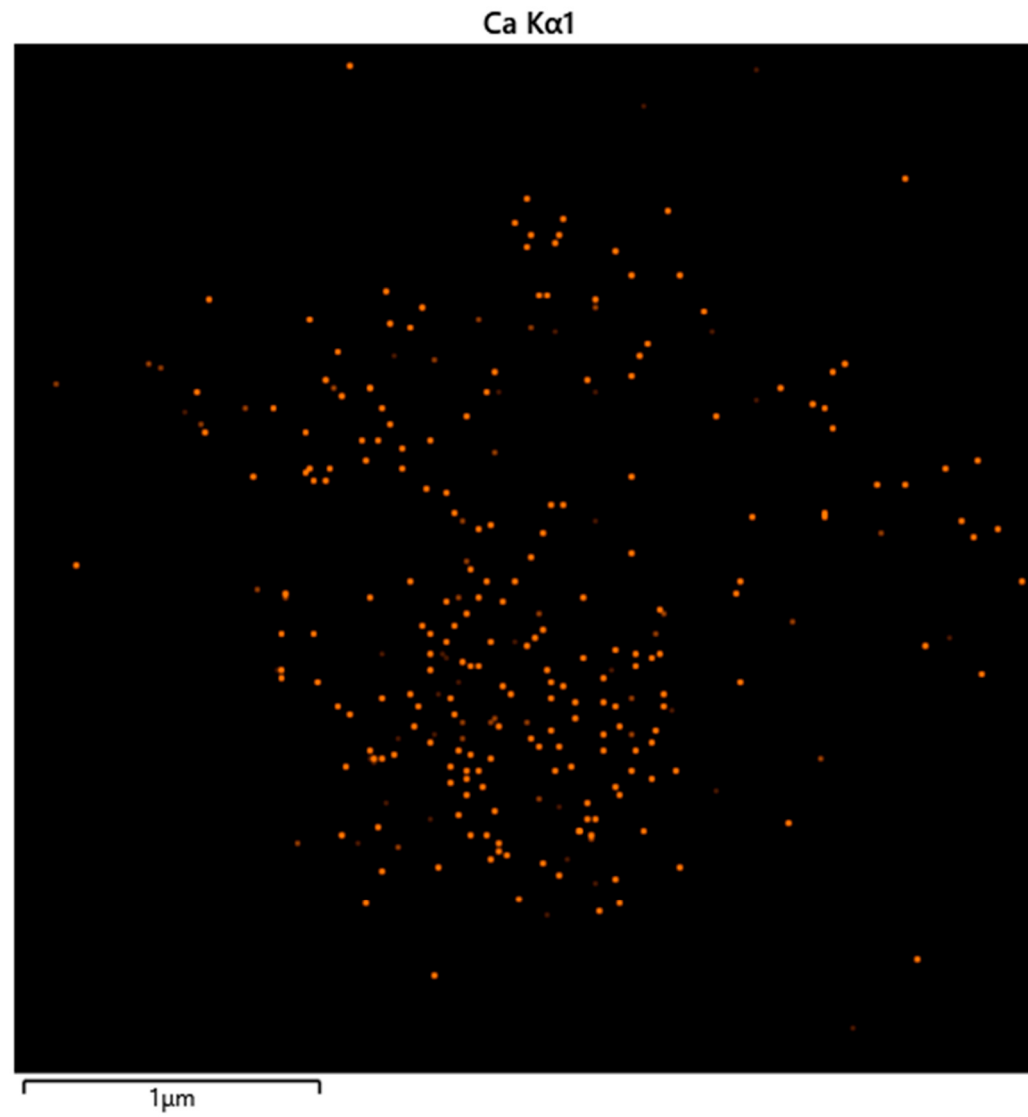

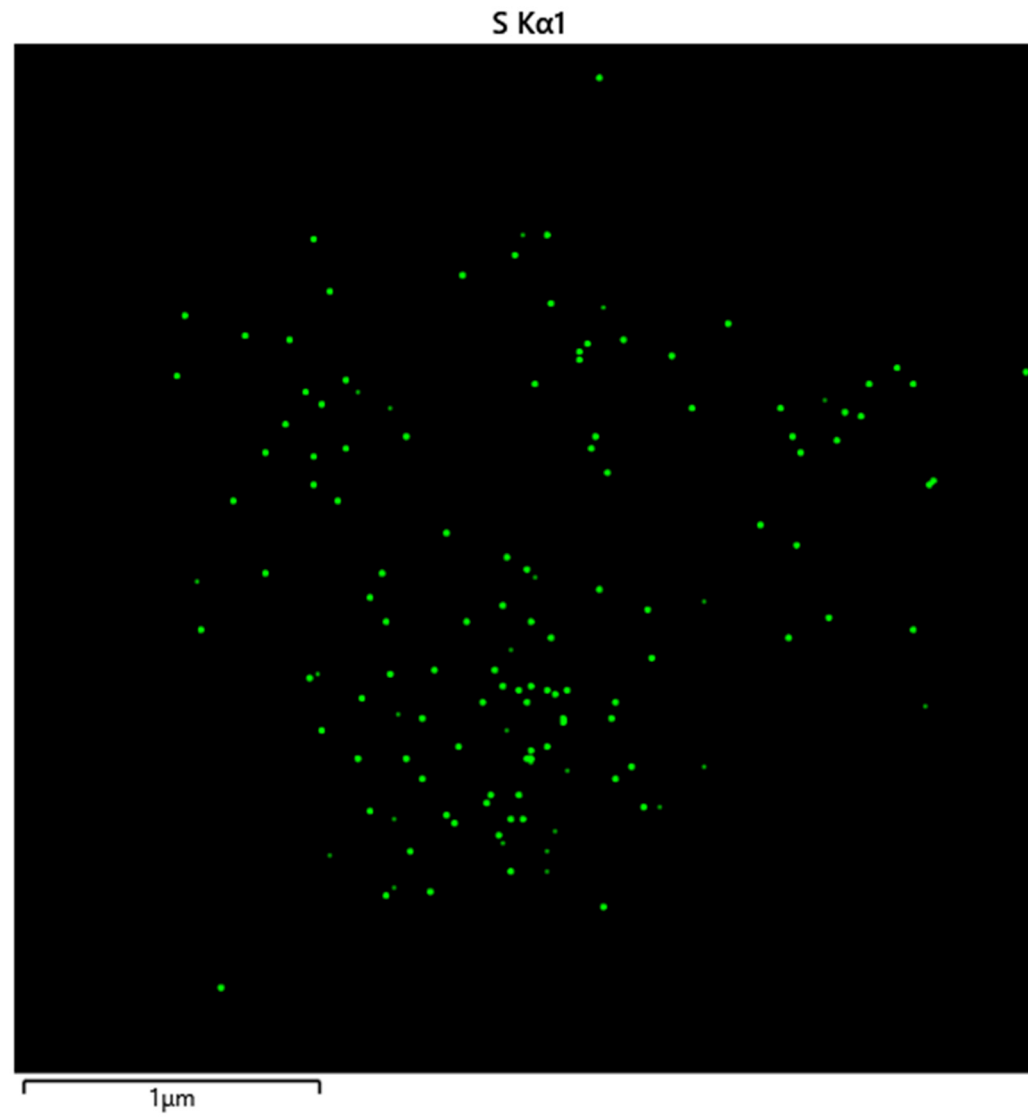

Supplement: Supplementary file 1 [file jox-15-00211-s001.zip › FileS1-Original images of Figures 3 and S2/FigureS2/FigureS2b SW2/2-mapping.pdf]

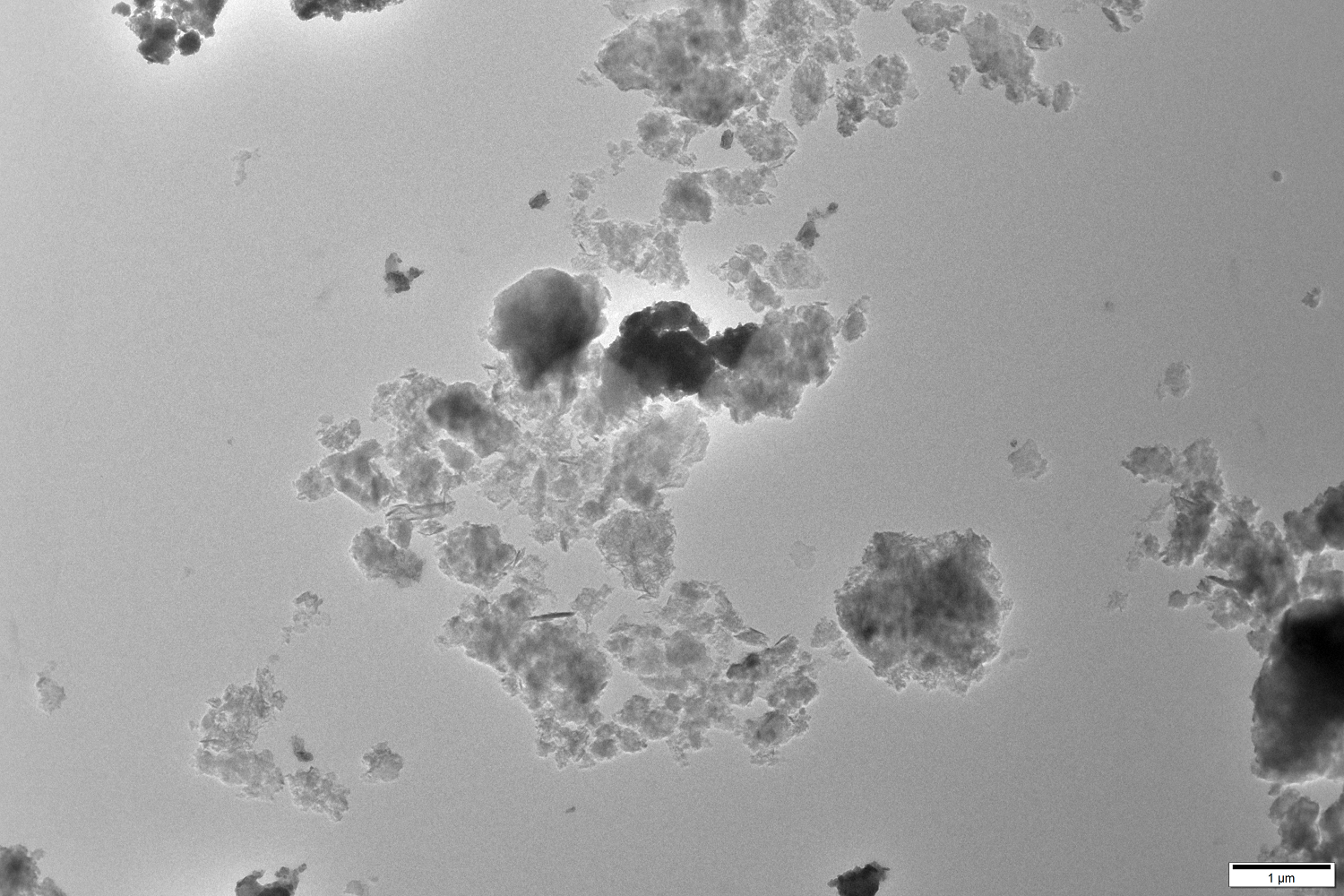

Supplement: Supplementary file 1 [file jox-15-00211-s001.zip › FileS1-Original images of Figures 3 and S2/FigureS2/FigureS2c SW3/3-1.tif]

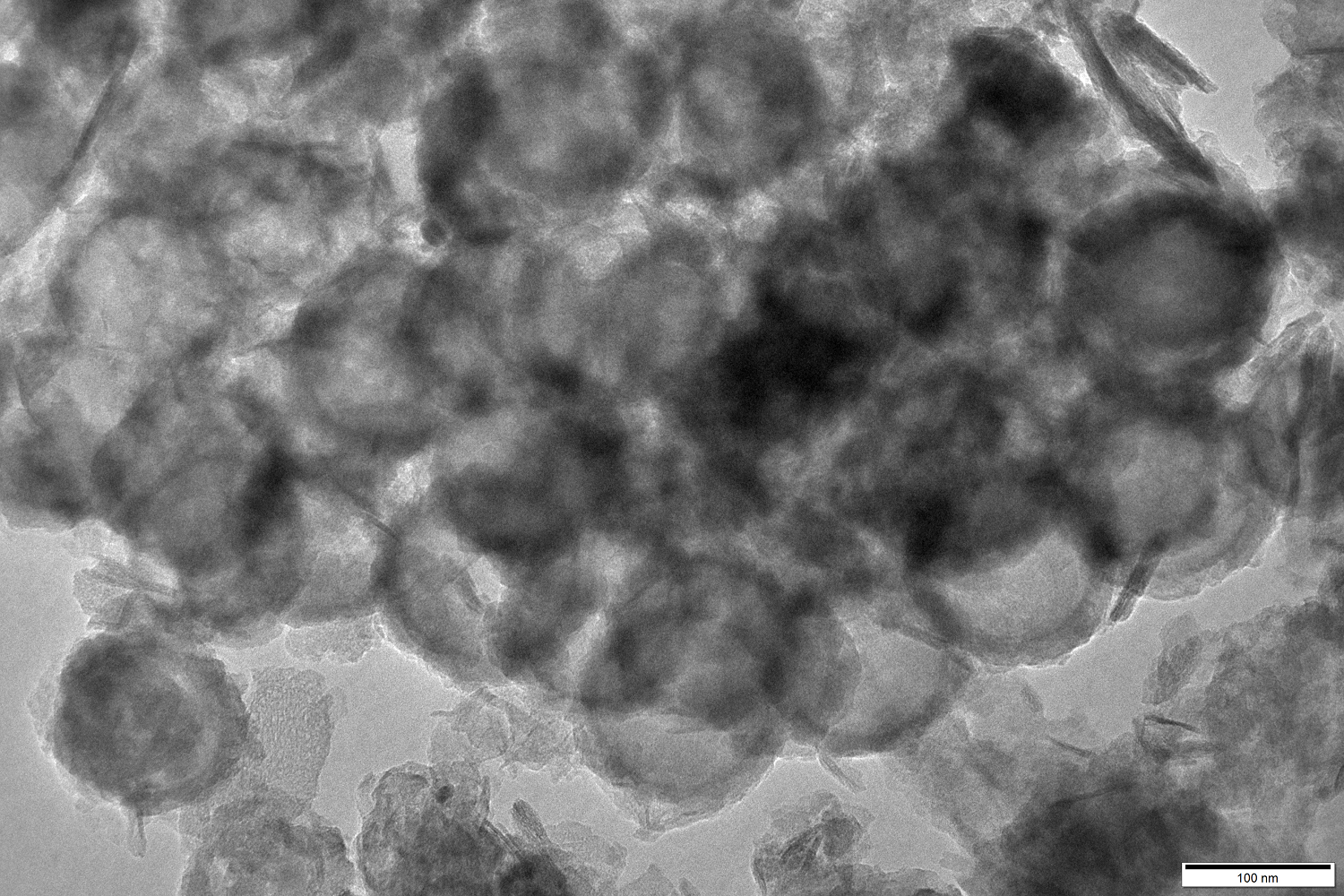

Supplement: Supplementary file 1 [file jox-15-00211-s001.zip › FileS1-Original images of Figures 3 and S2/FigureS2/FigureS2c SW3/3-2.tif]

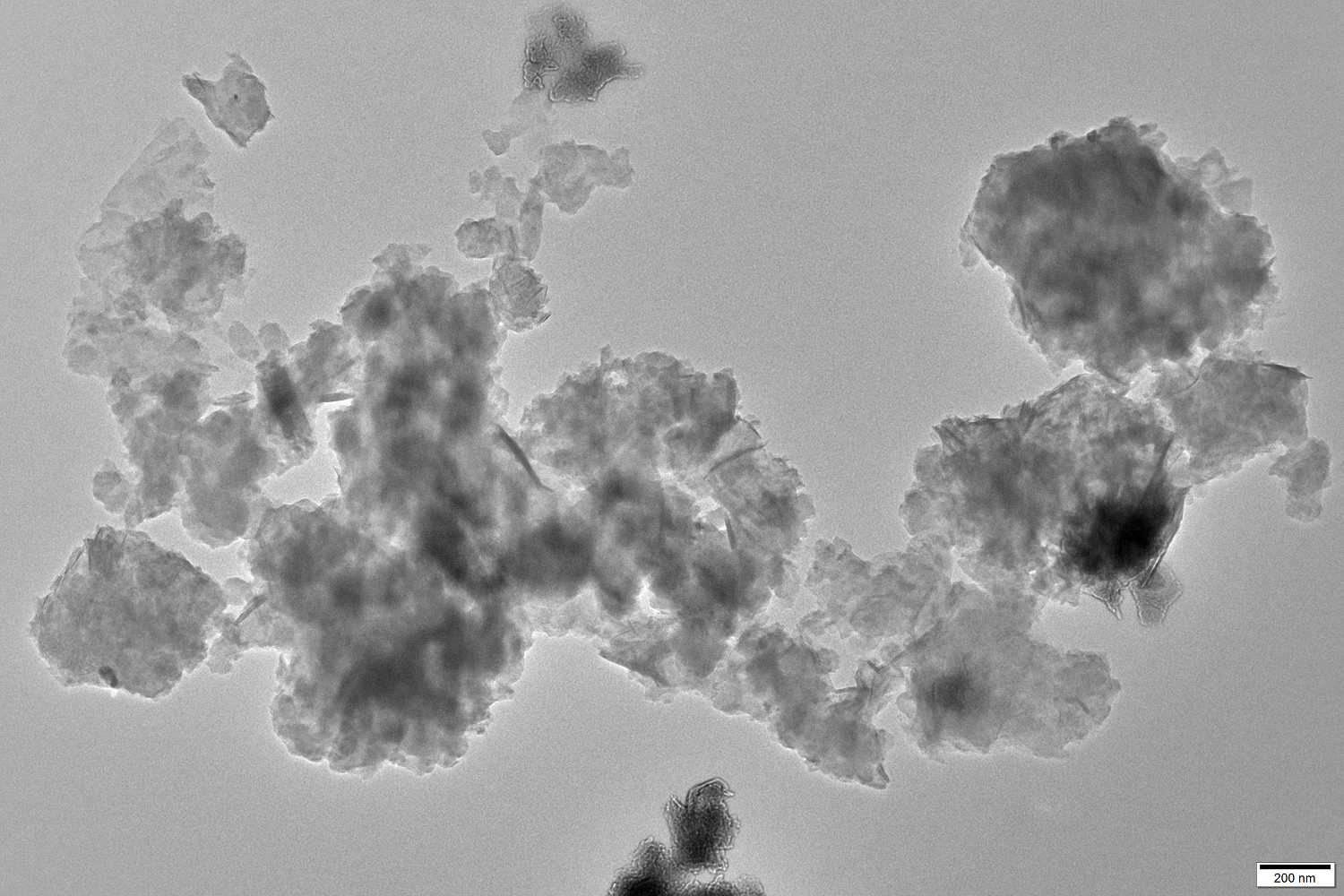

Supplement: Supplementary file 1 [file jox-15-00211-s001.zip › FileS1-Original images of Figures 3 and S2/FigureS2/FigureS2c SW3/3-3mapping.tif]

EDS 分层图像 4

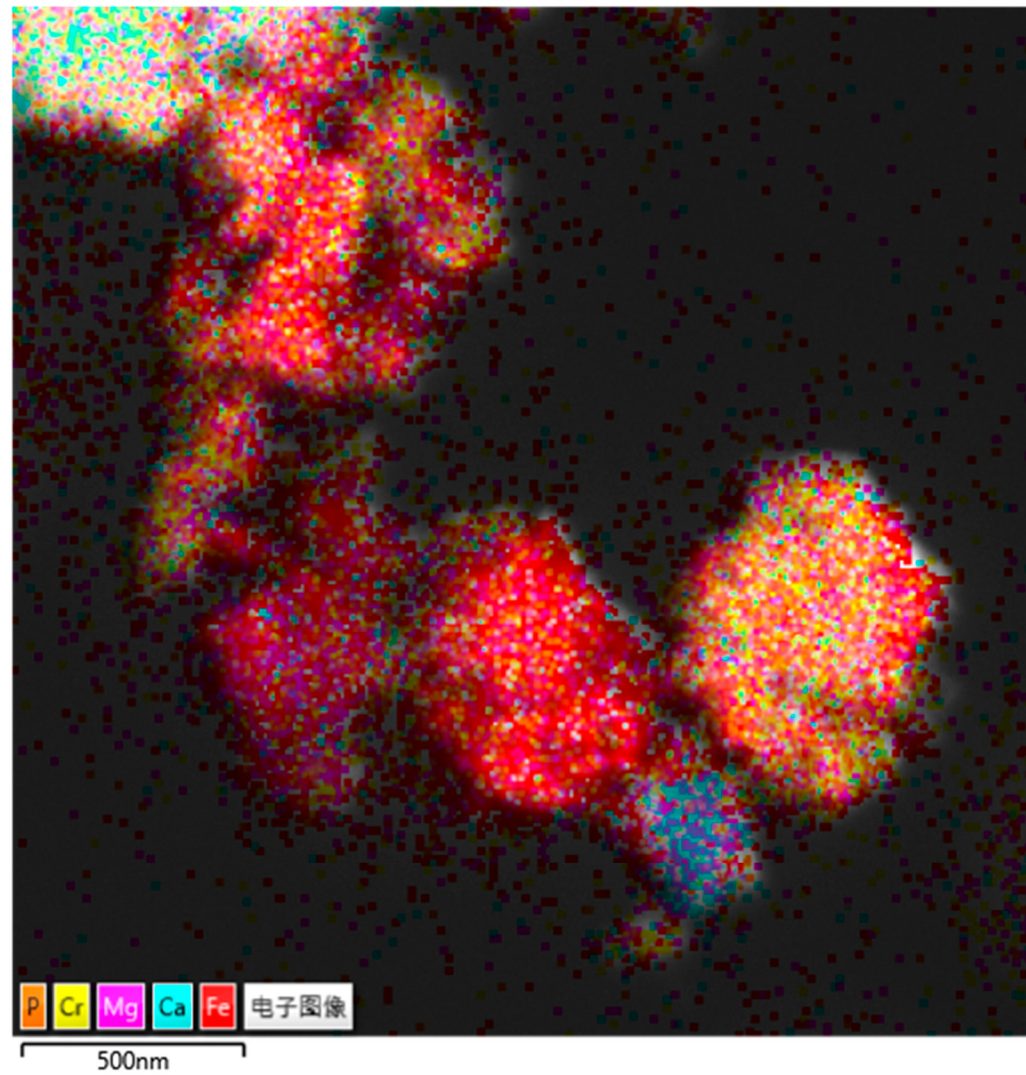

电子图像 4

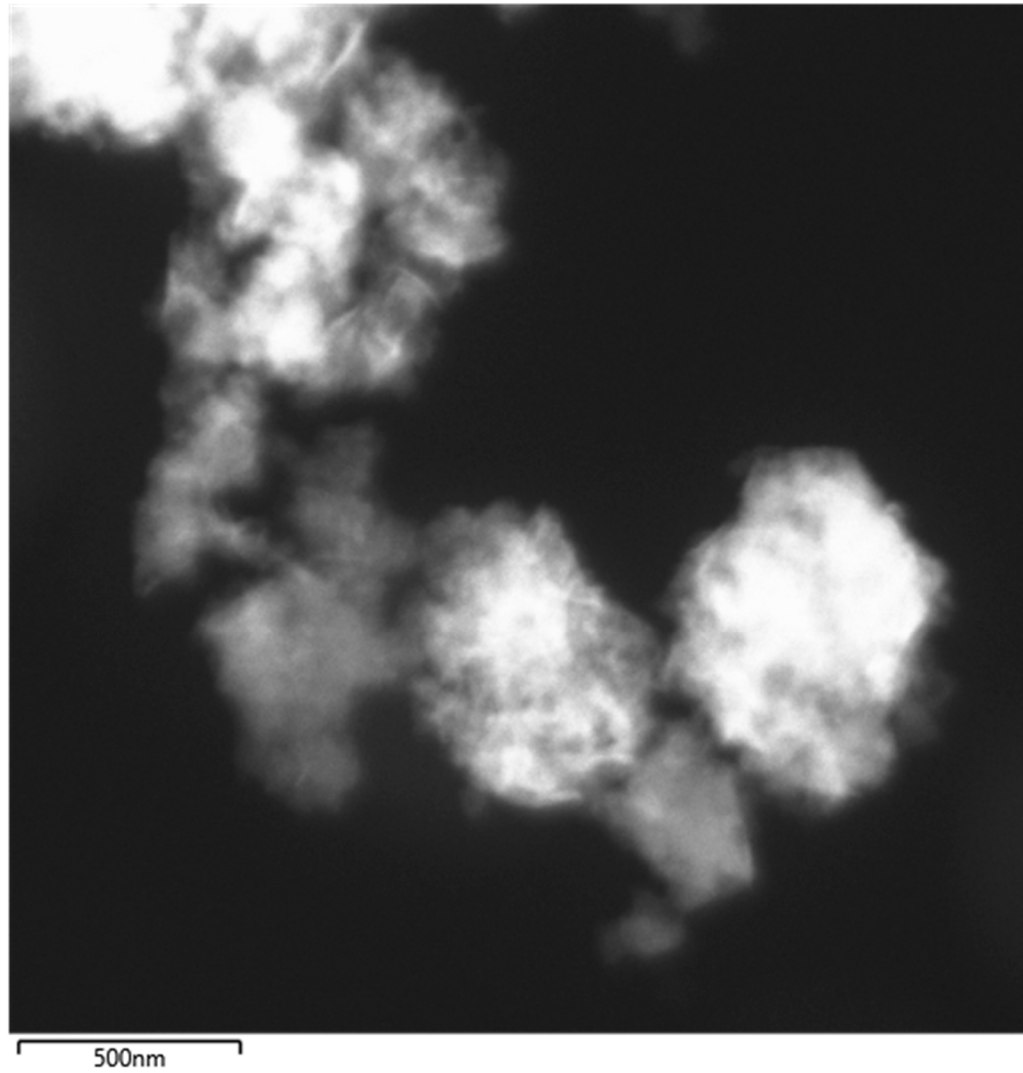

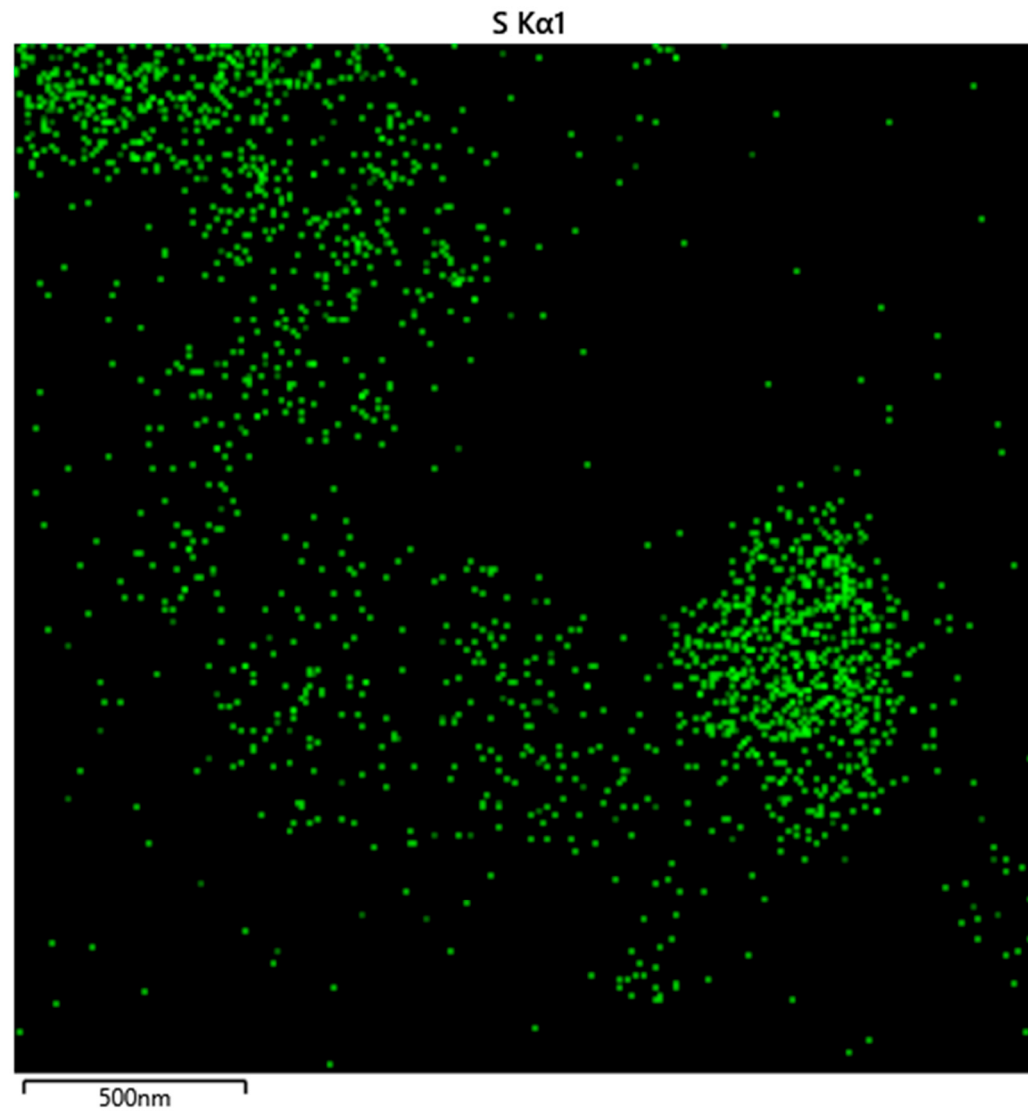

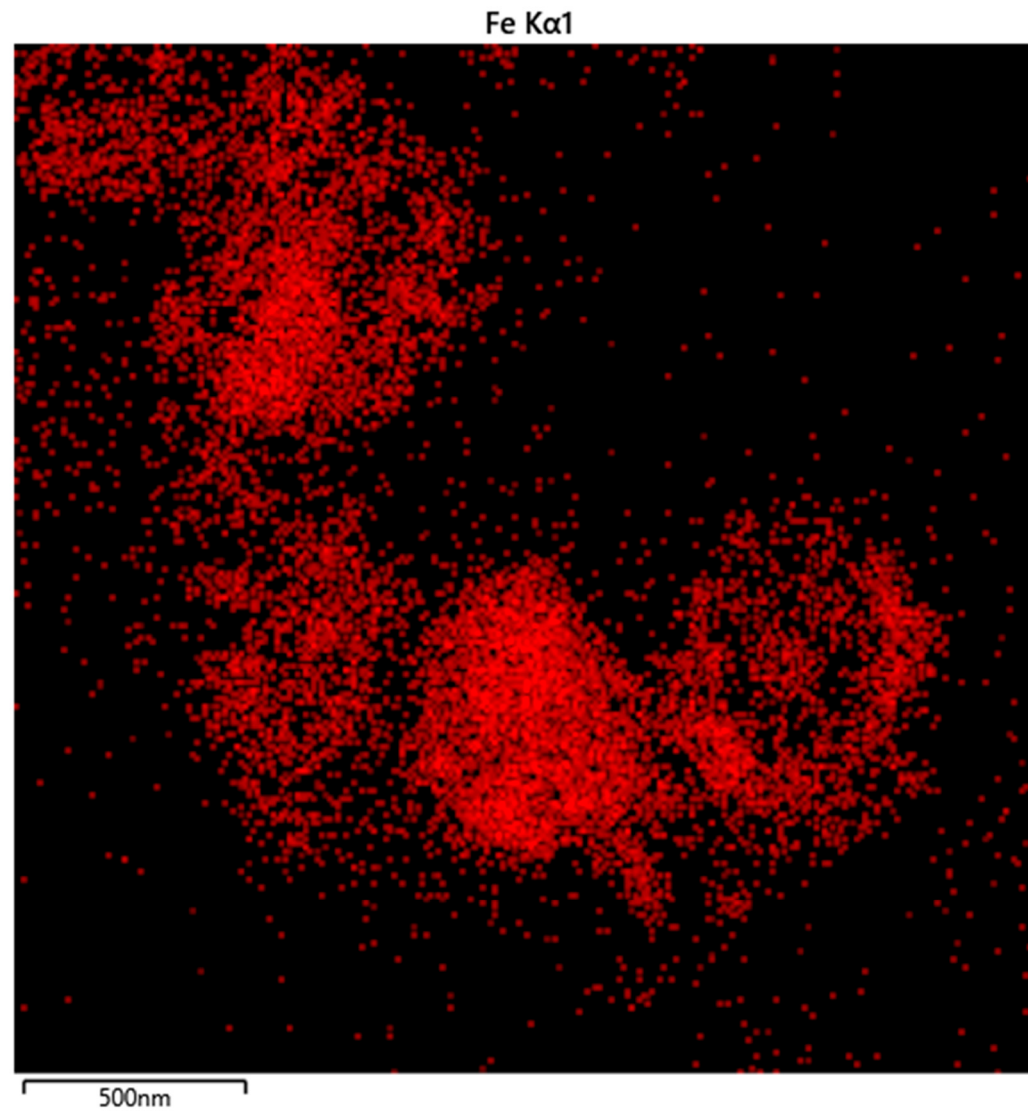

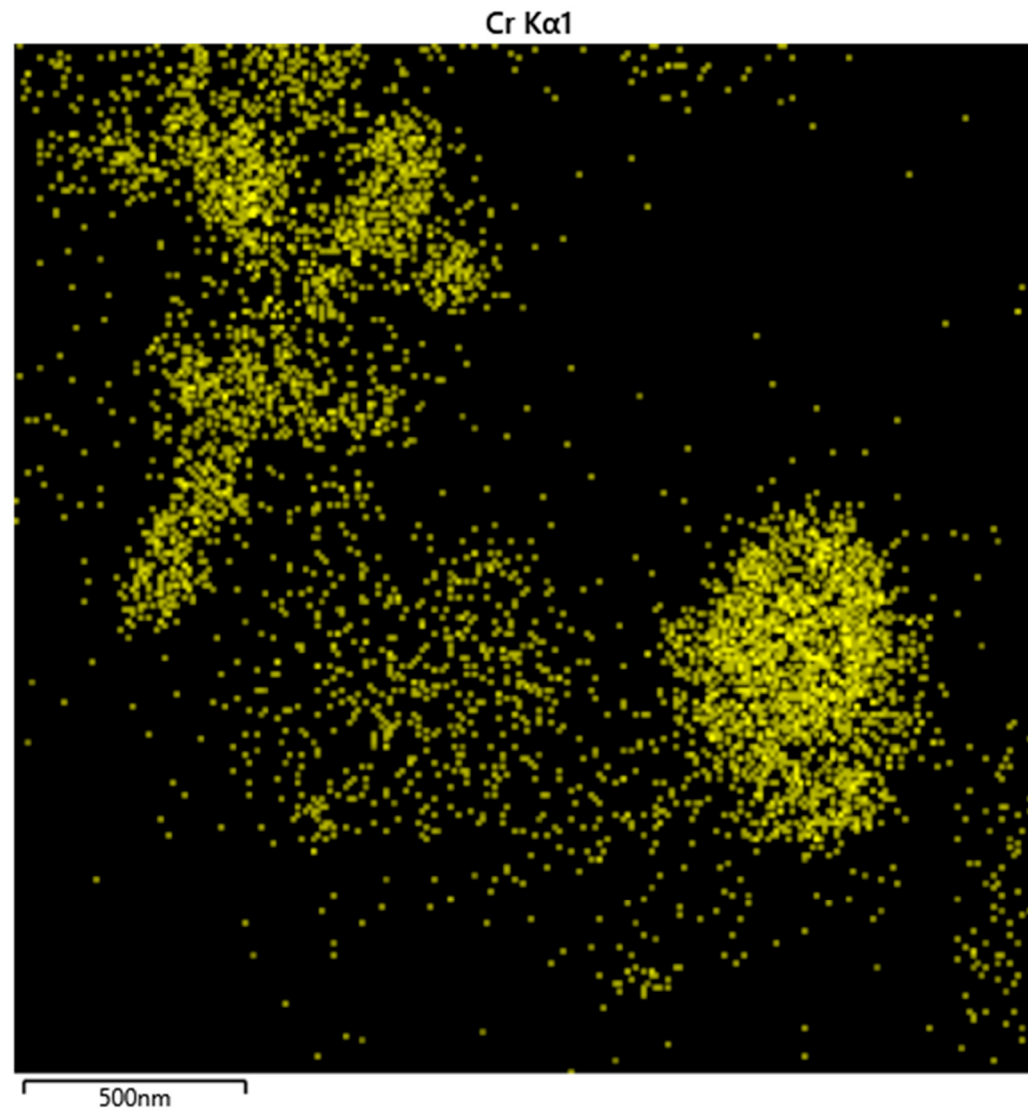

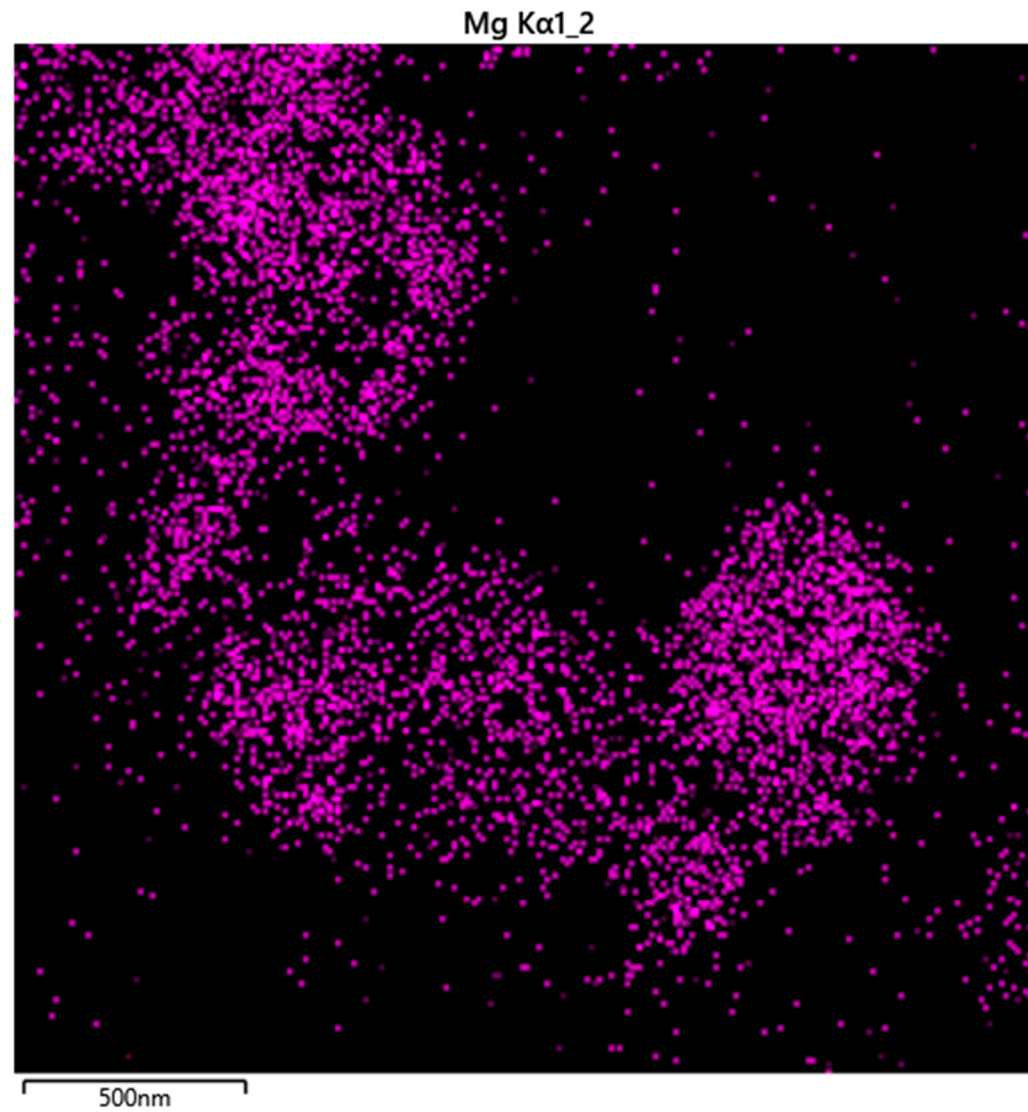

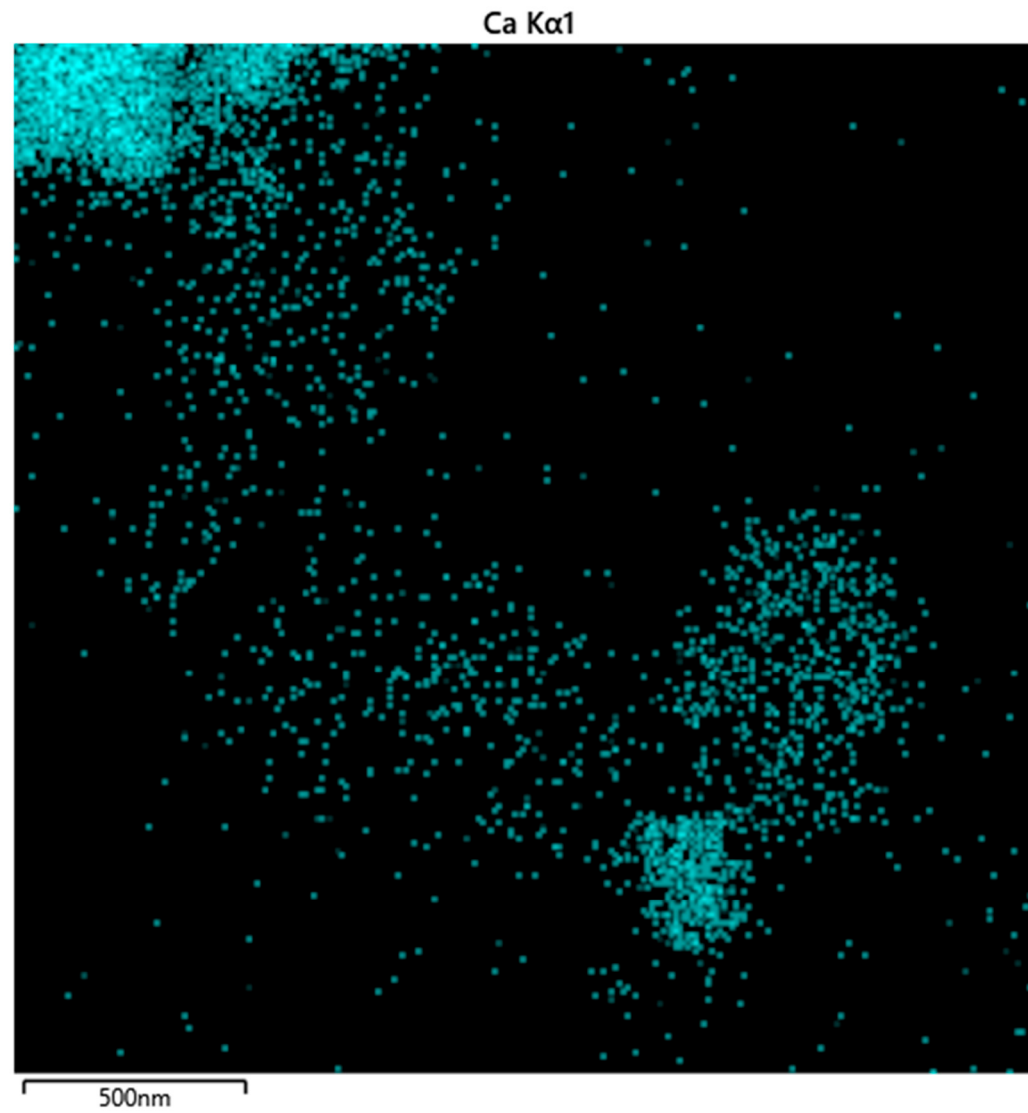

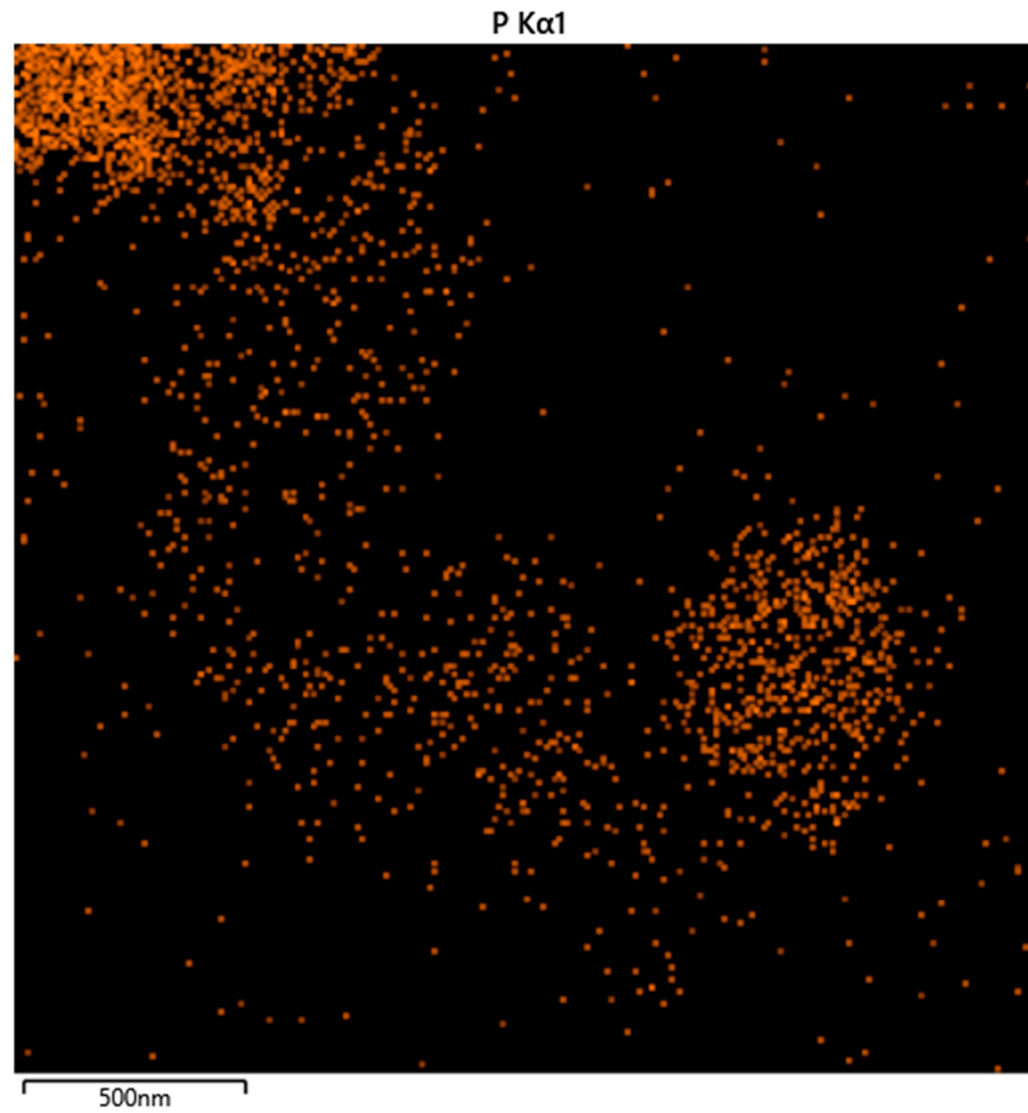

Supplement: Supplementary file 1 [file jox-15-00211-s001.zip › FileS1-Original images of Figures 3 and S2/FigureS2/FigureS2c SW3/3-mapping.pdf]

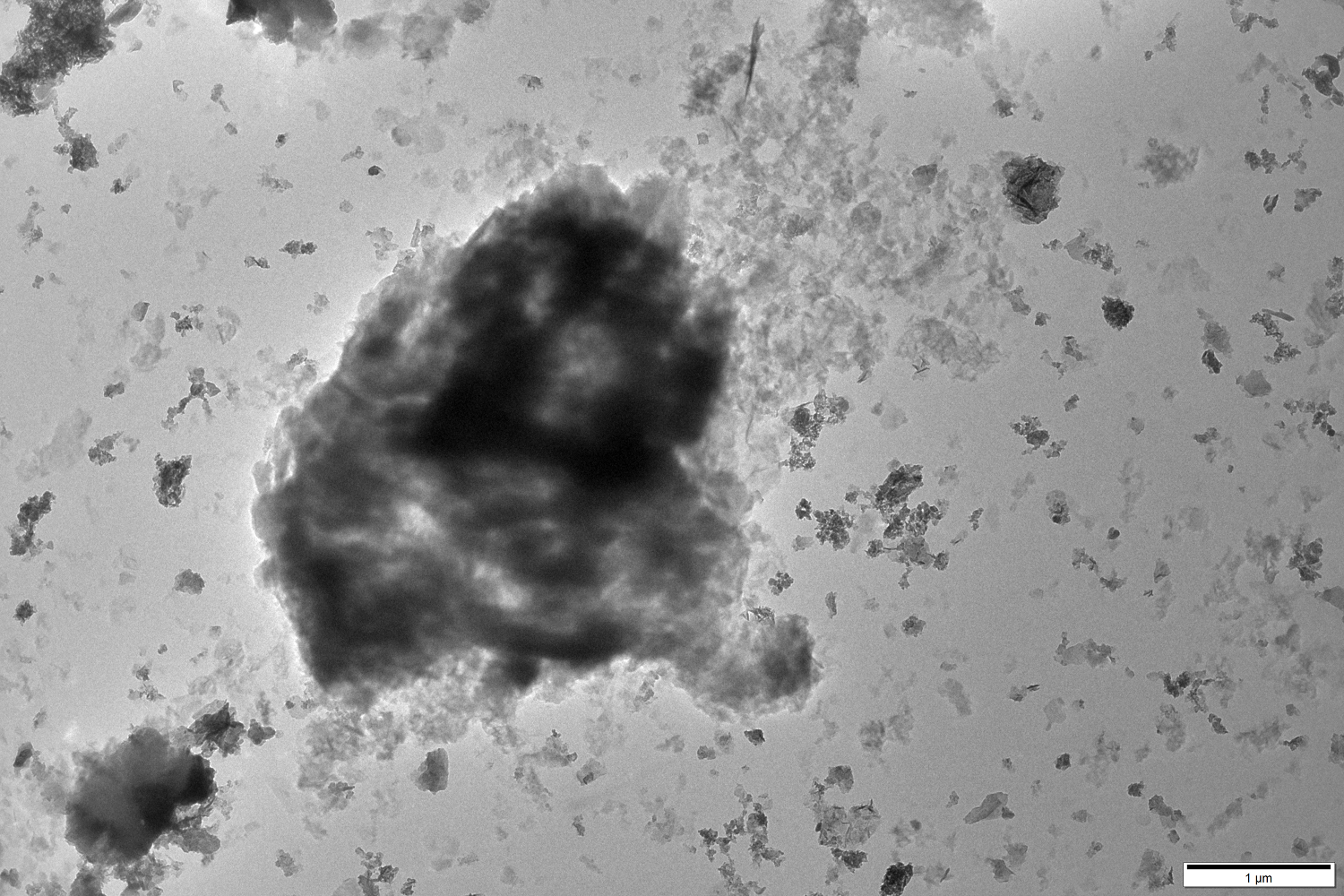

Supplement: Supplementary file 1 [file jox-15-00211-s001.zip › FileS1-Original images of Figures 3 and S2/FigureS2/FigureS2d SW4/4-1.tif]

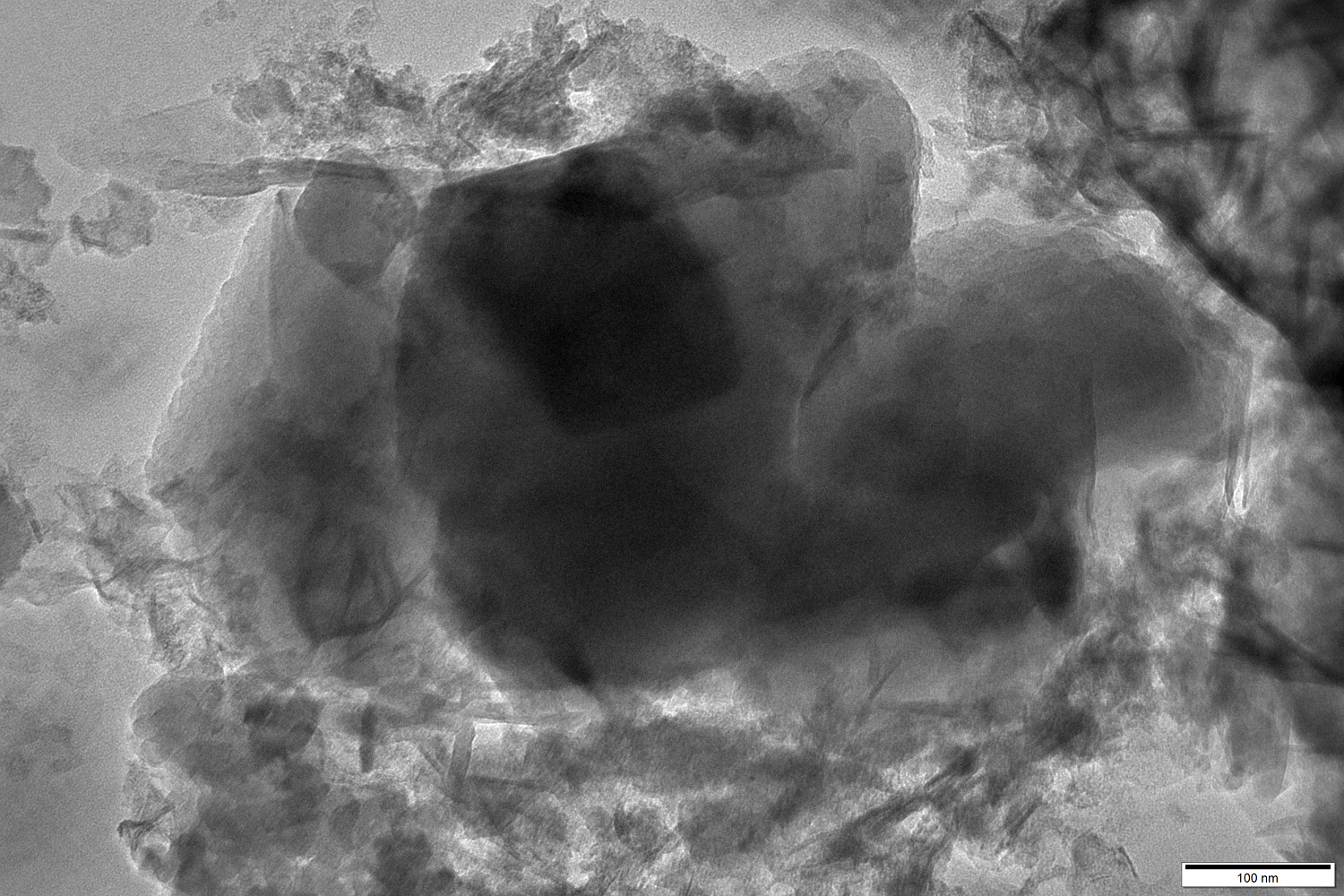

Supplement: Supplementary file 1 [file jox-15-00211-s001.zip › FileS1-Original images of Figures 3 and S2/FigureS2/FigureS2d SW4/4-2.tif]

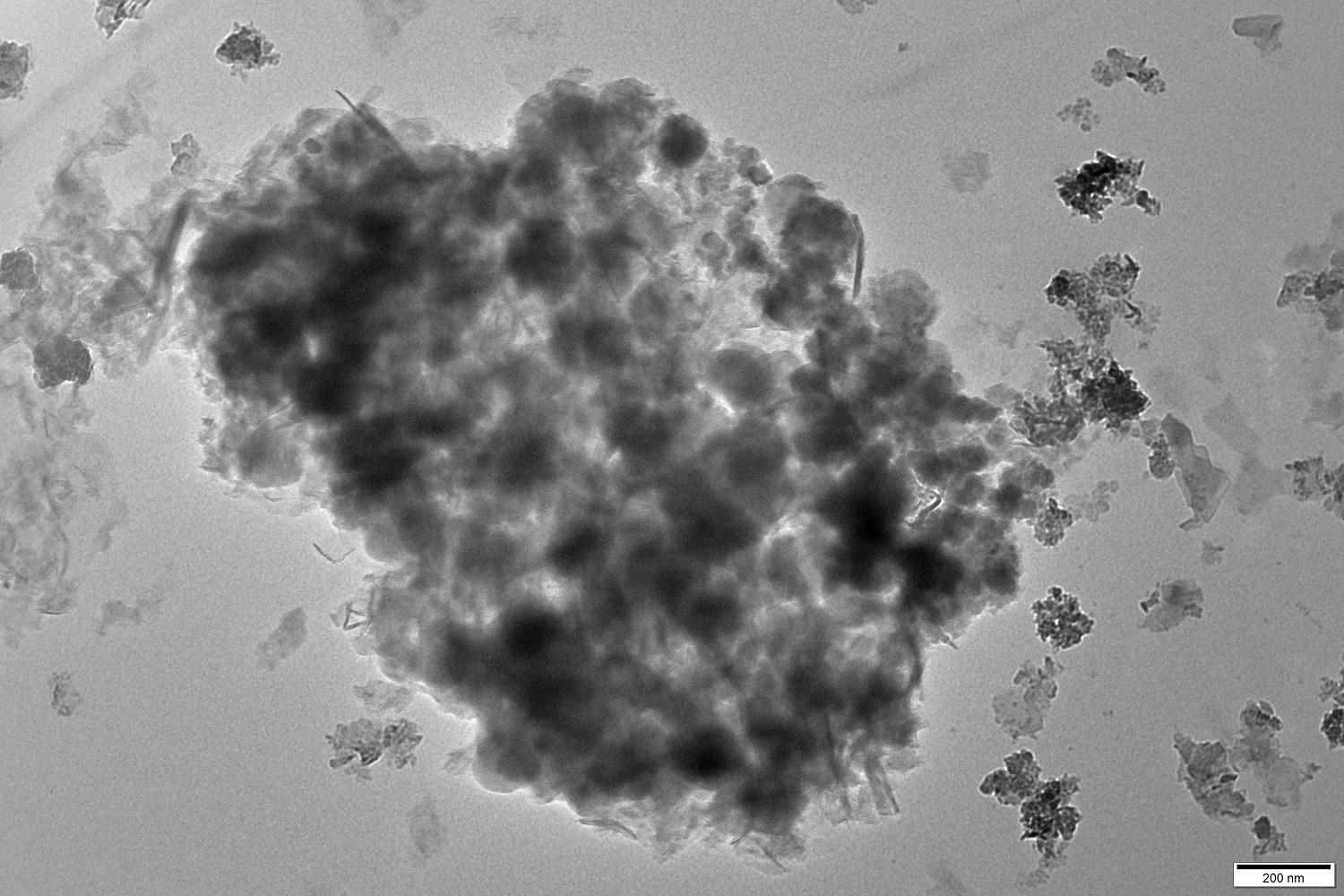

Supplement: Supplementary file 1 [file jox-15-00211-s001.zip › FileS1-Original images of Figures 3 and S2/FigureS2/FigureS2d SW4/4-3 mapping.tif]

EDS 分层图像 6

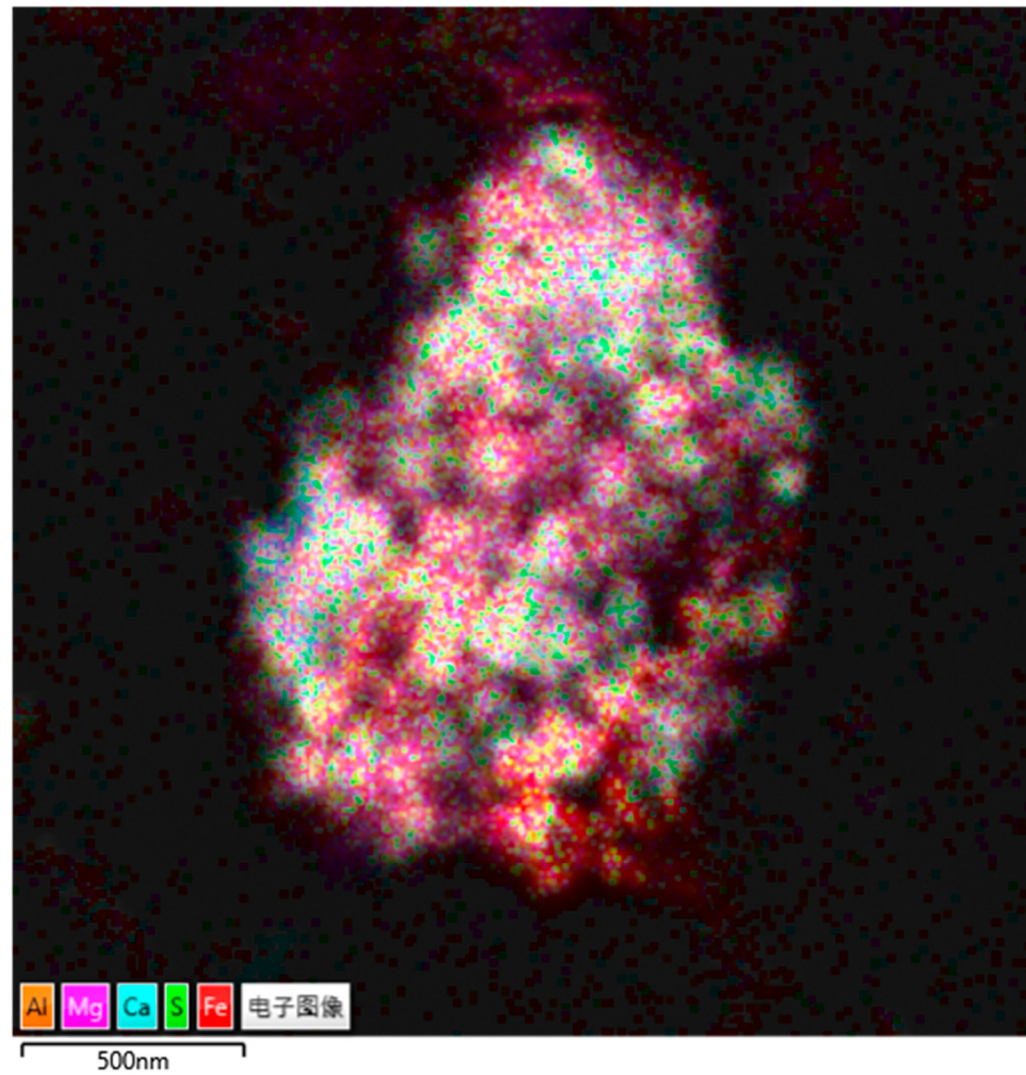

电子图像 6

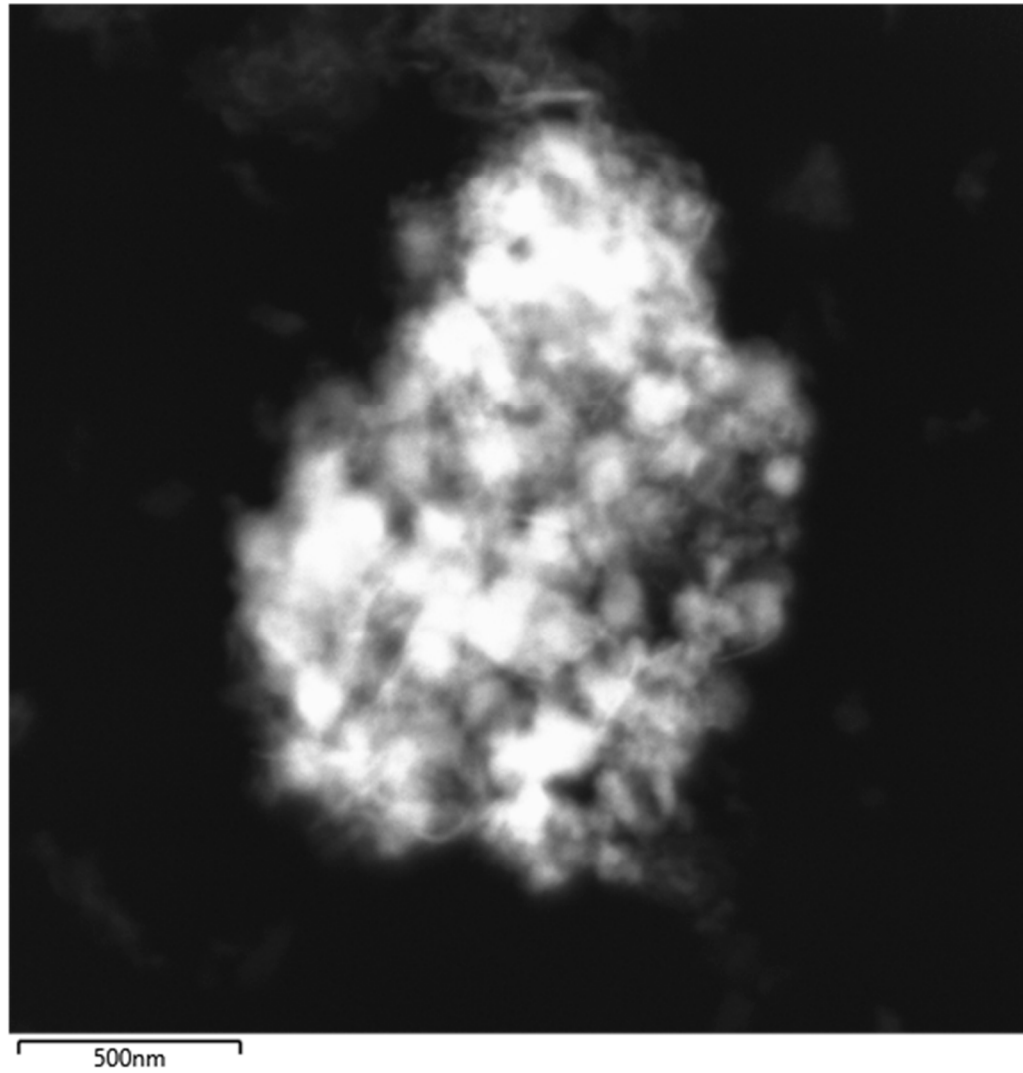

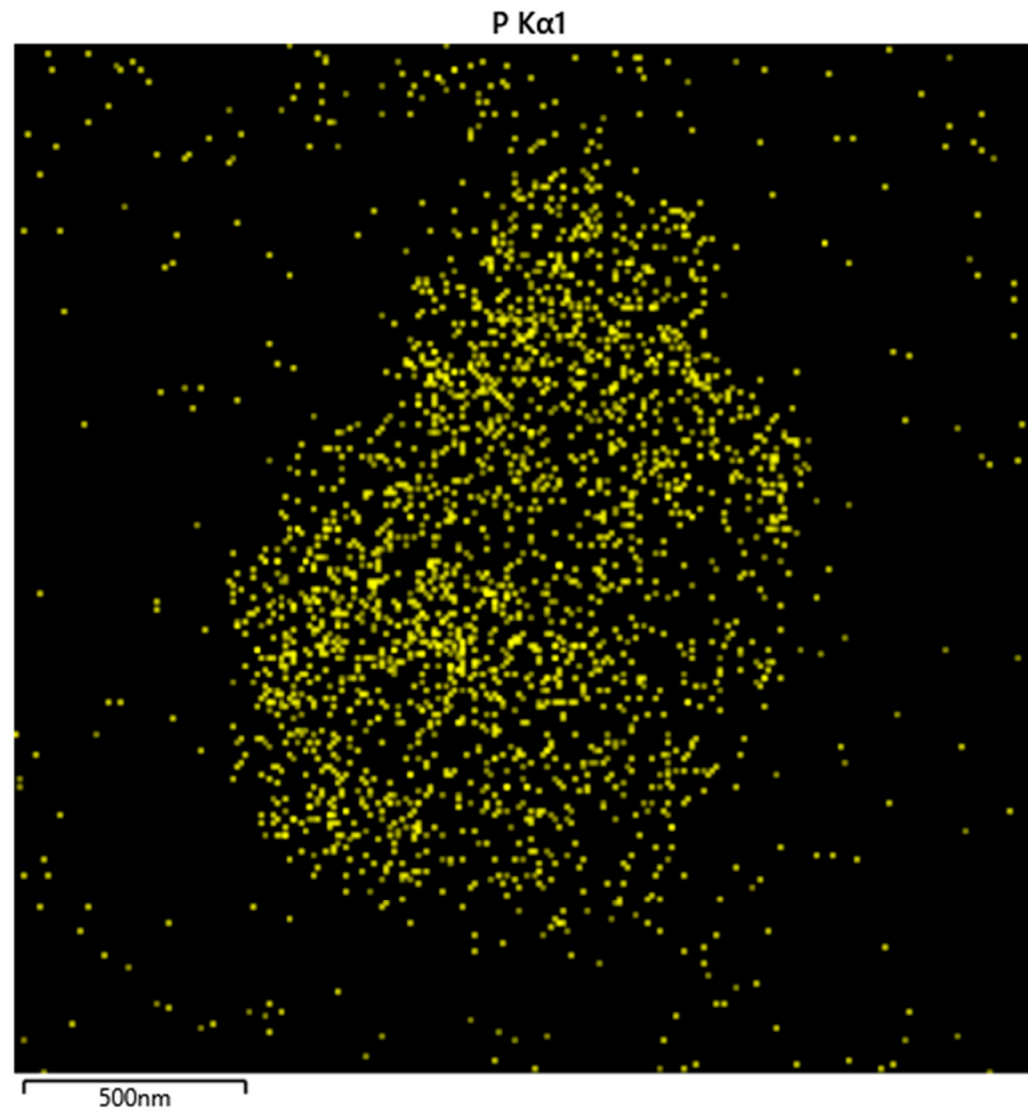

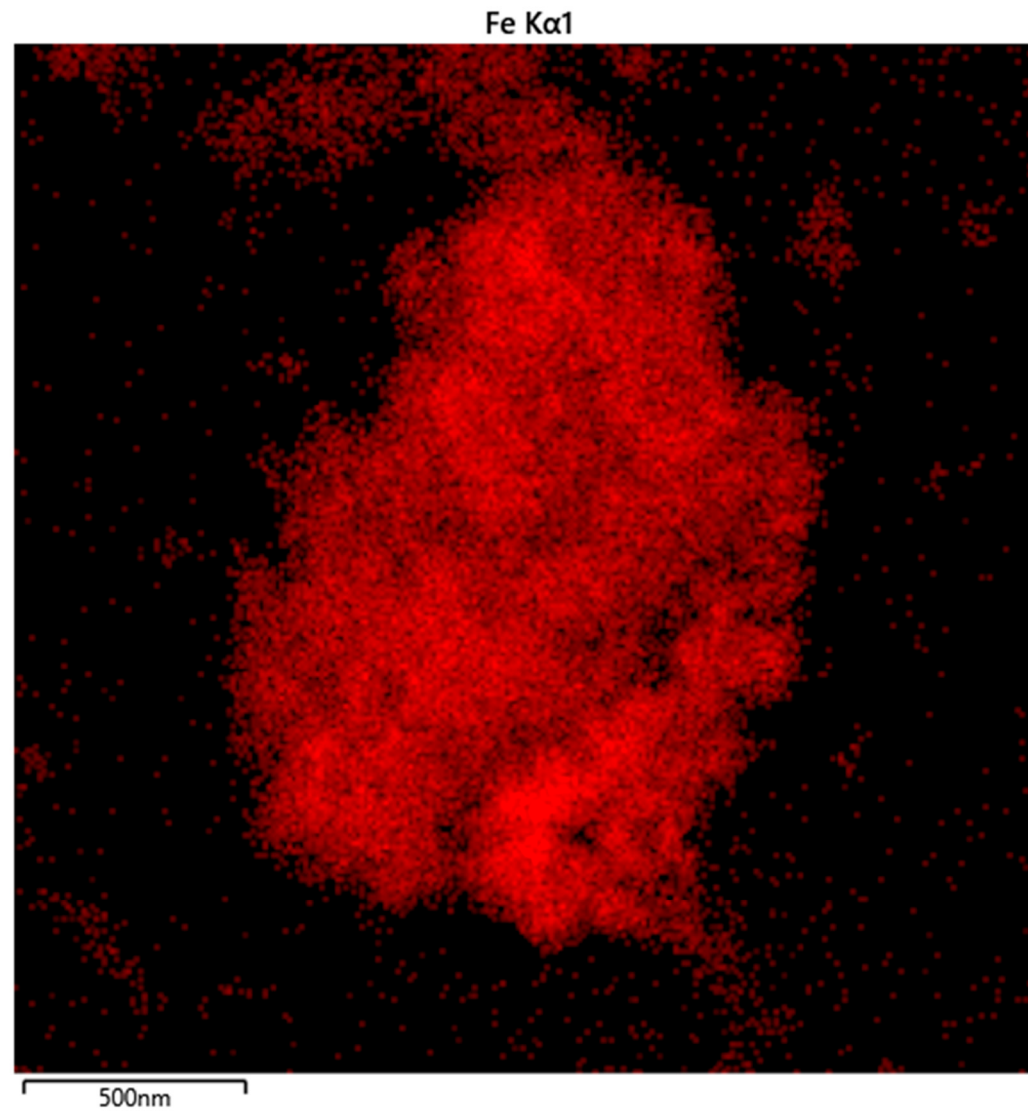

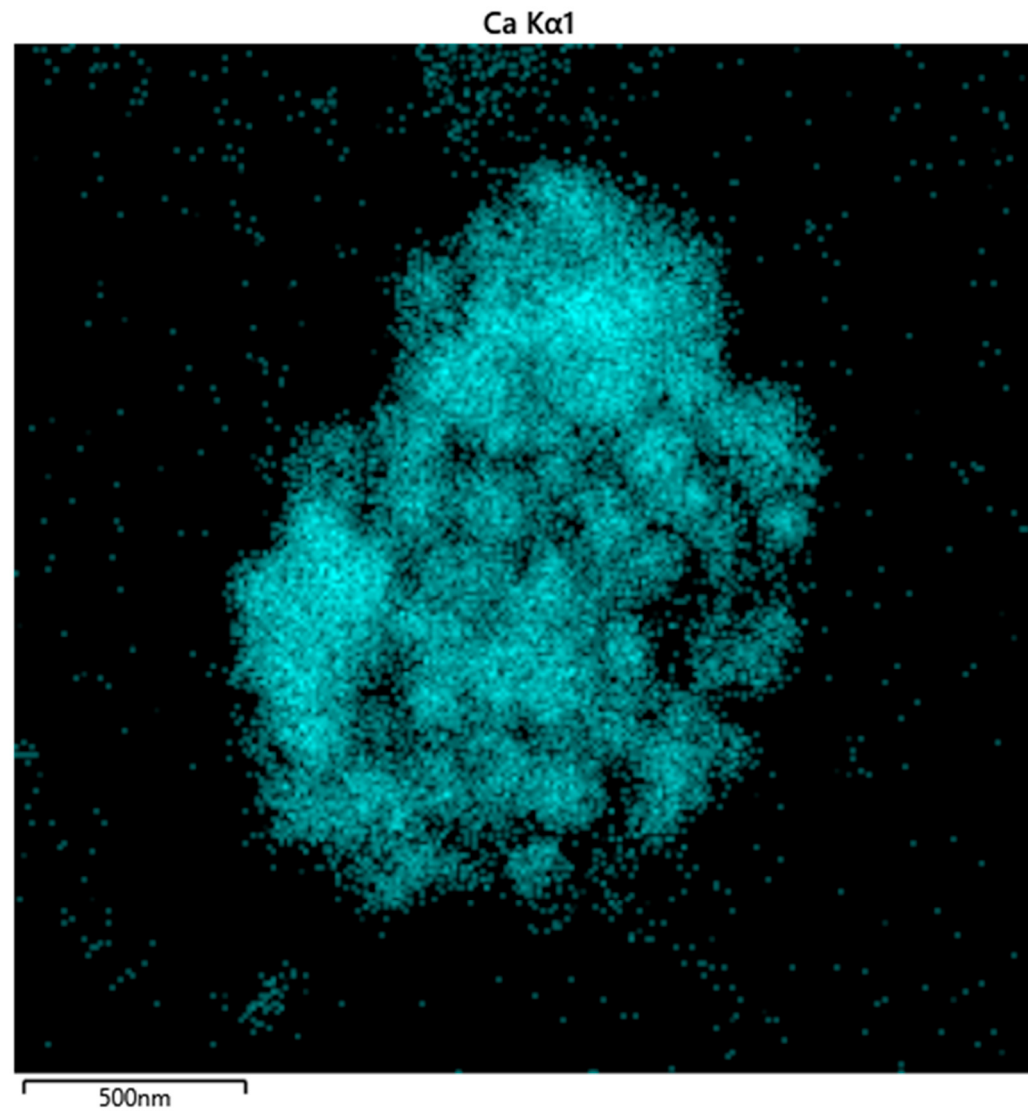

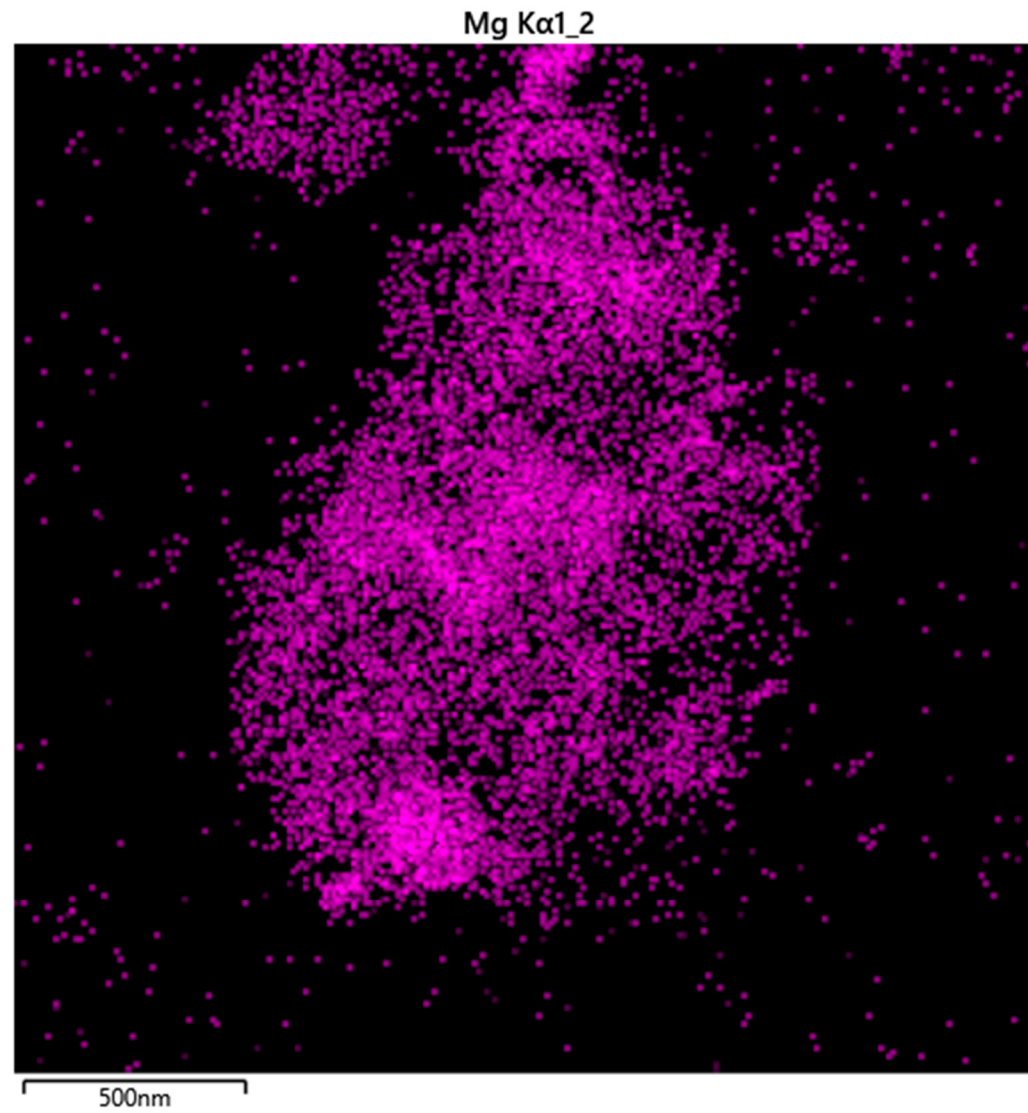

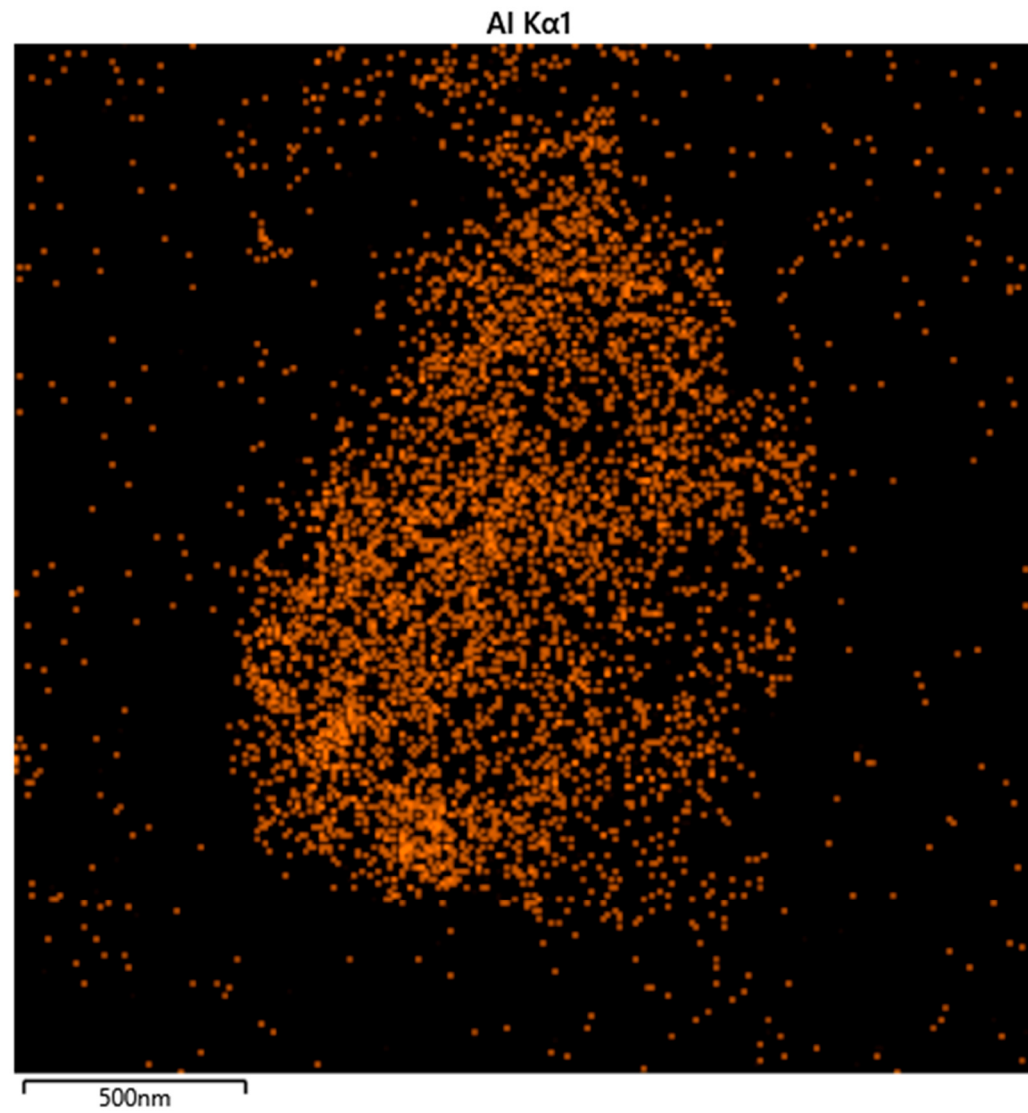

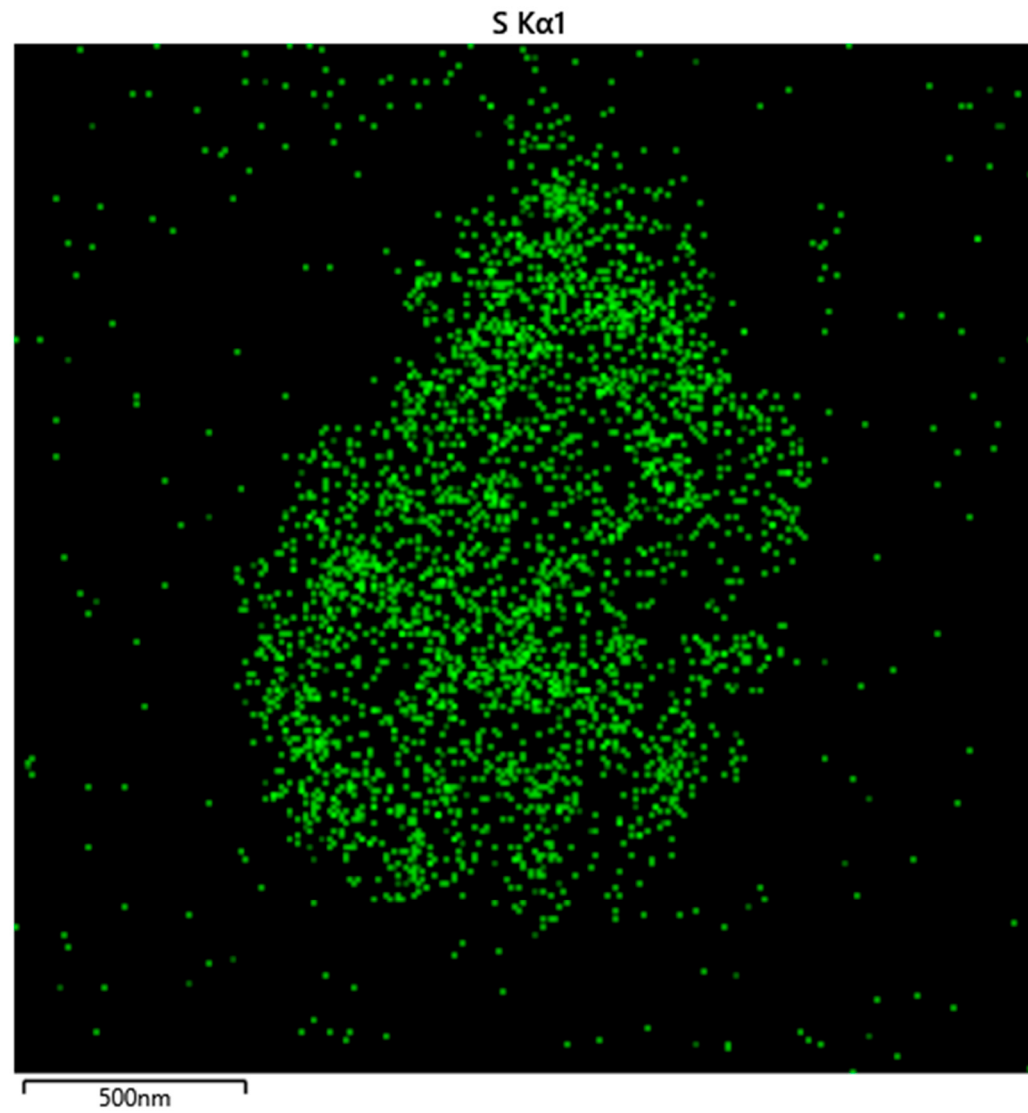

Supplement: Supplementary file 1 [file jox-15-00211-s001.zip › FileS1-Original images of Figures 3 and S2/FigureS2/FigureS2d SW4/4-mapping.pdf]
